# Computational prediction of cAMP receptor protein (CRP) binding sites in cyanobacterial genomes

**DOI:** 10.1186/1471-2164-10-23

**Published:** 2009-01-15

**Authors:** Minli Xu, Zhengchang Su

**Affiliations:** 1 Department of Bioinformatics and Genomics, Bioinformatics Research Center, the University of North Carolina at Charlotte, Charlotte, NC 28233, USA

## Abstract

**Background:**

Cyclic AMP receptor protein (CRP), also known as catabolite gene activator protein (CAP), is an important transcriptional regulator widely distributed in many bacteria. The biological processes under the regulation of CRP are highly diverse among different groups of bacterial species. Elucidation of CRP regulons in cyanobacteria will further our understanding of the physiology and ecology of this important group of microorganisms. Previously, CRP has been experimentally studied in only two cyanobacterial strains: *Synechocystis sp*. PCC 6803 and *Anabaena sp*. PCC 7120; therefore, a systematic genome-scale study of the potential CRP target genes and binding sites in cyanobacterial genomes is urgently needed.

**Results:**

We have predicted and analyzed the CRP binding sites and regulons in 12 sequenced cyanobacterial genomes using a highly effective *cis*-regulatory binding site scanning algorithm. Our results show that cyanobacterial CRP binding sites are very similar to those in *E. coli*; however, the regulons are very different from that of *E. coli*. Furthermore, CRP regulons in different cyanobacterial species/ecotypes are also highly diversified, ranging from photosynthesis, carbon fixation and nitrogen assimilation, to chemotaxis and signal transduction. In addition, our prediction indicates that *crp *genes in modern cyanobacteria are likely inherited from a common ancestral gene in their last common ancestor, and have adapted various cellular functions in different environments, while some cyanobacteria lost their *crp *genes as well as CRP binding sites during the course of evolution.

**Conclusion:**

The CRP regulons in cyanobacteria are highly diversified, probably as a result of divergent evolution to adapt to various ecological niches. Cyanobacterial CRPs may function as lineage-specific regulators participating in various cellular processes, and are important in some lineages. However, they are dispensable in some other lineages. The loss of CRPs in these species leads to the rapid loss of their binding sites in the genomes.

## Background

Cyclic AMP receptor protein (CRP), also known as catabolite gene activator protein (CAP), is an important transcriptional regulator widely distributed in a variety of bacterial groups [1,2]. The biological processes under the regulation of CRP are highly diverse, including energy metabolism [3,4], cell division and development [5], toxin production [1], competence development [6], quorum sensing [7] and cellular motility [8,9]. CRP belongs to the CRP/FNR transcription factor (TF) superfamily [10], which are generally believed to function as global regulators throughout the eubacteria [11]. Each member of the CRP/FNR superfamily contains an N-terminal effector binding domain and a C-terminal helix-turn-helix (HTH) DNA binding domain (DBD) [12]. The TFs of this superfamily form a homodimer *in vivo*, and are activated by the binding of specific small effector molecules to their effector binding domains [12]. The CRP dimer is activated by the binding of two cAMP molecules to the effector binding domain of each subunit, which causes a conformational change in the DBDs, allowing each to bind to half of a specific pseudo-palindromic DNA sequence in the promoters of the genes that are under CRP regulation [13]. Upon the binding, CRP interacts with the C-terminal domain of the alpha subunit of the RNA polymerase, affecting the RNA polymerase binding to the promoter, and thus leads to the change of the transcription initiation rate of the target gene [14-18].

The functions of CRP as well as its target genes (the CRP regulons) have been well studied in *E. coli *and other heterotrophic bacteria [19], and it seems that all the sequenced *E. coli *genomes encode one copy of the *crp *gene. CRP in *E. coli *is characterized as a global regulator, which controls the expression of more than 200 transcriptional units involved in various important biological processes of this organism [20,21]. Through decades of research, 269 CRP binding sites (RegulonDB release 5.8 [21]) in this species have been experimentally identified, which show a pseudo-palindromic consensus in the form of TGTGAN_6_TCACA. More recently, slightly different CRP binding sites with the consensus TGCGAN_6_TCGCA were also identified in *E. coli *and other γ-proteobacteria [22]. One of the major functions of CRP in *E. coli *involves the transcriptional regulation of genes related to organic carbon assimilation and energy metabolism [3,4].

As the life of *E. coli *and other heterotrophic organisms relies on the assimilation of organic carbon sources from the environment, it is not surprising that CRP works as an important global regulator to coordinate a variety of biological processes in these organisms. Cyanobacteria, on the other hand, are a group of autotrophic organisms capable of oxygenic photosynthesis; therefore, they do not rely on organic carbon source from the environment. Intriguingly, at least half of the sequenced cyanobacterial genomes encode at least one copy of the *crp *gene (see below). CRP proteins have been experimentally studied in two cyanobacterial strains, i.e. *Synechocystis sp*. PCC 6803 (PCC6803) [8,9,23-26] and *Anabaena sp*. PCC 7120 (PCC7120) [27,28]. In the PCC6803 genome, the open reading frame (ORF) *sll1371 *encodes a homologue to the *E. coli crp *gene [23], and has been named *sycrp1*. It has been shown that the product of this gene, SyCRP1 forms a homodimer, which can bind cAMP with high affinity *in vitro *[24]. Furthermore, in the presence of cAMP, SyCRP1 could form a complex with DNA that contains the consensus CRP binding site similar to that in *E. coli *(TGTGAN_6_TCACA) [24]. Further studies have revealed that SyCRP1 was essential for type IV pilus biogenesis and was involved in cell motility in PCC6803 [8,9,25,29,30]. On the other hand, in the PCC7120 genome two ORFs, *alr0295 *and *alr2325*, were found to encode putative CRPs, and were named *ancrpA *and *ancrpB*, respectively [27]. Equilibrium dialysis measurements showed that both AnCRPA and AnCRPB (the gene products of *ancrpA *and *ancrpB *respectively) could bind cAMP. Electrophoresis mobility shift assay (EMSA) further demonstrated that AnCrpA could bind to the consensus CRP binding site in *E. coli *[27]. It has also been reported that both AnCrpA and AnCrpB are functional in PCC7120, the former regulates the expression of several genes involved in nitrogen fixation [28], and the latter controls the genes induced by nitrogen depletion [31]. A few CRP binding sites in these two genomes have also been experimentally determined, which were found to form a palindromic motif with the consensus sequence TGTGAN_6_TCACA similar to that in *E.coli *[25,28]. In addition, the promoter regions of most of these identified CRP-activated genes in these two cyanobacterial genomes also contain an *E. coli *-10 σ^70^-like box (TAN_3_T), located ~22 bp downstream the CRP binding site. These studies also suggested that CRPs in cyanobacteria might regulate a very different set of genes than those in *E. coli*. However, a systematic genome-scale study of the potential CRP target genes as well as CRP binding sites in cyanobacteria is hitherto lacking. In this paper, we have predicted the CRP regulons as well as CRP binding sites in 12 sequenced cyanobacterial genomes that encode at least one copy of the *crp *gene using a highly effective motif scanning algorithm [32,33]. We have also investigated the degradation of the CRP binding sites in the rest of sequenced cyanobacterial genomes in which the *crp *genes were lost during the course of evolution.

## Methods

### 1. Materials

Genome sequences, predicted ORFs and annotation files of the following 29 cyanobacterial genomes were downloaded from the NCBI website at : *Acaryochloris marina *MBIC11017 (MBIC11017), *Anabaena variabilis *ATCC 29413 (ATCC29413), *Anabaena sp*. PCC 7120 (PCC7120), *Gloeobacter violaceus *PCC 7421 (PCC7421), *Prochlorococcus marinus str*. AS9601 (AS9601), *Prochlorococcus marinus str*. MIT 9211 (MIT9211), *Prochlorococcus marinus str*. MIT 9215 (MIT9215), *Prochlorococcus marinus str*. MIT 9301 (MIT9301), *Prochlorococcus marinus *MIT9303 (MIT9303), *Prochlorococcus marinus *MIT9312 (MIT9312), *Prochlorococcus marinus str*. MIT 9313 (MIT9313), *Prochlorococcus marinus str*. MIT 9515 (MIT9515), *Prochlorococcus marinus str*. NATL1A (NATL1A), *Prochlorococcus marinus str*. NATL2A (NATL2A), *Prochlorococcus marinus *CCMP1375 (CCMP1375), *Prochlorococcus marinus *MED4 (MED4), *Synechococcus elongatus *PCC 6301 (PCC6301), *Synechococcus elongatus *PCC 7942 (PCC7942), *Synechococcus sp*. CC9311 (CC9311), *Synechococcus sp*. CC9605 (CC9605), *Synechococcus sp*. JA-2-3B'a(2–13) (B-Prime), *Synechococcus sp*. JA-3-3Ab (A-Prime), *Synechocystis sp*. PCC 6803 (PCC6803), *Synechococcus sp*. CC9902 (CC9902), *Synechococcus sp*. RCC307 (RCC307), *Synechococcus sp*. WH 7803 (WH7803), *Synechococcus sp*. WH8102 (WH8102), *Thermosynechococcus elongatus *BP-1 (BP-1), and *Trichodesmium erythraeum *IMS101 (IMS101).

### 2. Prediction of operons

We predicted operon structures in each cyanobacterial genomes using the Operon Finder Software (OFS) developed by Westover *et al *[34]. OFS predicts operons based on three pieces of information, including the intergenic distance, functional relatedness of gene annotations, and conserved gene neighborhoods. In this study, both a multi-gene operon and a singleton operon containing only one gene are referred as a transcription unit (TU).

### 3. Prediction of orthologues

A simple bidirectional best hit (BDBH) approach using the BLASTP program with an E-value cutoff 10^-10 ^was used for the prediction of orthologous genes between each pair of genomes.

### 4. Phylogenetic analysis

To construct the CRP tree, the full length amino acid sequences of cyanobacterial CRPs were identified by the criteria described above using SyCRP1 (sll1371) in PCC6803 as the query sequence. Multiple sequence alignments of the identified cyanobacterial CRP sequences and that of *E. coli *K12 were performed using ClustalW implemented in MEGA [35] with default settings. A neighbor-joining (NJ) tree with Poisson correction was constructed using the MEGA program with the *E. coli *CRP (GI:16131236) being the outgroup. To construct the tree in Figure S1 (see Additional file 1), we used SyCRP1 as the query sequence to search the RefSeq database using BLASTP with an E-value cutoff 10^-7^. If there were multiple hits from a species, the hit with the smallest E-value was identified as the CRP in that species. The resulting sequences were used to construct an un-rooted tree in the similar way as described above. To construct the species tree, the DNA sequences of the 16S rRNA genes of the sequenced cyanobacteria and that of *E. coli *K12 were aligned using ClustalW with manual refinement. After the indels were discarded, the final alignments contain 1311 positions. A Neighbor-Joining tree of the 16 rRNA gene sequences was constructed with the *E. coli *K12 sequence being the outgroup using Kimura 2-parameter model. Statistical significance at each node in the trees was evaluated using 1000 bootstrap resamplings.

### 5. Phylogenetic footprinting and construction of the profile of CRP binding sites in cyanobacteria

A previous screening of the target genes of SyCRP1 in PCC6803 using microarray showed that 18 genes in 13 putative TUs were down-regulated in a *sycrp1 *disruptant compared with the wild type strain [25]. Our preliminary manual scanning of the upstream promoter regions of these TUs revealed that four of them contain a putative CRP binding site similar to those in *E. coli *(the first four TUs in Table 1). The rest nine TUs that do not contain putative CRP binding sites are likely to be regulated indirectly by CRP through different regulators. In addition, since the *crp *gene is auto-regulated in *E. coli *as well as in many other species [36], we also manually scanned the upstream inter-TU region of *sycrp1 *(*sll1371*) and identified a putative CRP binding site in it (Table 1). We thus used the orthologues (if they exist and were identified by the BDBH method) of these five TUs for the phylogenetic footprinting of CRP binding sites in cyanobacterial genomes.

**Table 1:** Transcriptional units in PCC6803 used for the initial phylogenetic footprinting analysis.

ORF(s) within TU	Putative CRP binding site^1^	Position^2^
slr1667 slr1668 ssr2786	**TGTGA**TCTGGG**TCACA**	-245
slr2015 slr2016 slr2017 slr2018	**GGTGT**TTATTG**TCACA**	-346
sll0443 sll0444 sll0445 sll0446 sll0447 sll0448 sll0449	**GGTGA**TTAAGT**TCCCA**	-371
slr0442	**TGTGA**TCCAGA**TCACA**	-189
sll1371 sll1372 sll1373	**AGTGA**AAAAAC**TCACT**	-143

1. Bold, the highly conserved positions within the palindromic CRP binding sites.

2. Position of the putative CRP binding site relative to the first codon in the TU.

For this purpose, we pooled the entire upstream inter-TU regions of these five TUs in PCC6803, as well as those of TUs containing their orthologous genes in other cyanobacterial genomes which encode at least one *crp *gene. Motif finding programs including CUBIC [37] and BioProspector [38], were then applied to predict CRP binding sites in these sequences. CUBIC is a graph theoretic based algorithm that identifies highly similar *k*-mers in a set of pooled sequences; while BioProspector uses a Gibbs sampling strategy to find overrepresented *k*-mers in a set of pooled sequences. Putative CRP binding sites with high scores were manually picked up from the motif finding results, and were used to build a preliminary profile of the CRP binding sites in cyanobacterial genomes.

Since the number of binding sites used to construct the preliminary profile of CRP binding sites was relatively small (for the reason, see the Results section), in order to minimize possible bias of binding site sampling, we conducted a one-round iteration to obtain a more representative profile of the CRP binding sites. To this end, we scanned cyanobacterial genomes with the preliminary profile using the techniques described below, and picked the high scoring sites from each genome to construct the more representative profile of the CRP binding sites. We then used this final profile for genome-scale predictions of CRP binding sites in the cyanobacterial genomes.

### 6. Genome-wide prediction of CRP binding sites

The whole genome screening of all possible CRP binding sites were performed using an algorithm that we have developed previously [32]. The design of this algorithm is to enhance the prediction specificity by integrating the information of co-occurrence of multiple binding sites in the upstream region of a gene and that in the upstream regions of its orthologues in related genomes. Briefly, the final profile of the CRP binding sites obtained above was first used to scan all the inter-TU regions of the cyanobacterial genomes. The best motif was returned for each inter-TU region. Then the 19 to 31 bp downstream region of each putative binding site was further scanned for an *E. coli *-10 σ^-70 ^like box (TAN_3_T) using a corresponding profile that we have constructed previously from cyanobacterial genomes [32], to which a σ-factor of RNA polymerase is likely to bind to transcribe the downstream TU. Then, the upstream inter-TU regions of the orthologues of the genes in that particular TU in other cyanobacterial genomes were scanned for similar CRP binding sites and -10 like boxes. A score that combines these three pieces of information was computed to rank the putative CRP binding sites for each possible CRP-regulated TU.

Specifically, let *M *be the final profile of the CRP binding sites obtained above. For each predicted transcription unit *U*(*g*_1_, ..., *g*_*n*_) containing genes *g*_1_, ..., *g*_*n *_in a genome *G*, we extract the entire upstream intergenic region (if that region is larger than 800 bp, then only the 800 bp upstream the translation starting site were extracted) and denoted it as $I_{U(g_{1},...,g_{n})}$. We also extracted a random DNA sequence with the same length as $I_{U(g_{1},...,g_{n})}$ from the coding region, denoted as $C_{U(g_{1},...,g_{n})}$. We say that $I_{U(g_{1},...,g_{n})}$ and $C_{U(g_{1},...,g_{n})}$ are associated with *U*(*g*_1_, ..., *g*_*n*_) and with each genes *g*_1_, ..., *g*_*n *_as well. All the extracted $I_{U(g_{1},...,g_{n})}$ from one genome are denoted as set *I*_*U*_, and all the $C_{U(g_{1},...,g_{n})}$ in the same genome are denoted as set *C*_*U*_. For each *t *∈ *I*_*U *_or *t *∈ *C_U_*, we scan for possible CRP bindings sites using the profile *M*. The score of a putative binding site found in *t *by scanning with profile *M *is defined as,

(1) $$s_{M}(t)=\underset{h\subset t}{\max}\sum_{i=1}^{l}I_{i}\ln\frac{p(i,h(i))}{q(h(i))},$$

(2) $$I_{i}=(\sum_{b\in \{A,C,G,T\}}p(i,b)\ln\frac{p(i,b)}{q(b)})/a,$$

(3) $$a=\frac{n+1}{n+4}\ln(n+1)-\ln(n+4)-\frac{1}{n+4}\sum_{b\in \{A,C,G,T\}}\ln q(b)-\frac{n}{n+4}\ln\underset{b\in \{A,C,G,T\}}{\min}q(b),$$

where *l *is the length of the binding sites of *M*, *h *any substring of *t *with length *l*, *h*(*i*) the base at the *i*-th position of *h*, *p*(*i, b*) the relative frequency of base *b *occurring at position *i *in *M*, *q*(*b*) the background frequency of base *b*, and *n *the number of sequences used to construct *M*. When computing *p*(*i, b*), a pseudo count 1 is added to the frequency of each base at each position, and *a *is for normalization to keep *I*_*i *_within the range of [0,1].

When multiple profiles are considered for scanning *t*, we sum up the individual *S*_*M*_(*t*) s as defined by

(4) $$s_{M_{1}...M_{z}}(t)=\sum_{j=1}^{z}s_{M_{j}}(t).$$

For this study, we use *M*_1 _for the CRP binding sites and *M*_2 _for the -10 like box (TAN_3_T).

For the inter-TU sequence *t *associated with *U*(*g*_1_, ..., *g*_*n*_) in genome *G*, if *g*_*i *_has orthologues in *m*_*i *_closely related genomes $G_{1},...,G_{m_{i}}$, we denote *o*_*k*_(*g*_*i*_) as the inter-TU sequence upstream the TU containing *g*_*i *_'s orthologue in genome *G*_*k*_. When the presence of similar motifs in *o*_*k*_(*g*_*i*_) is also considered, the score of co-occurrence of the multiple binding sites in *t *is redefined as

(5) $$s(t)=s_{M_{1}...M_{z}}(t)+\underset{1<i<n}{\max}\sum_{j=1}^{z}\sum_{k=1}^{m_{i}}\frac{l_{j}-d_{i,j,k}}{m_{i}l_{j}}s_{M_{j}}(o_{k}(g_{i})),$$

where *d*_*i*, *j*, *k *_is the Hamming distance between the sequence found by using profile *M*_*j *_in *t *and *o*_*k*_(*g*_*i*_), *l*_*j *_the length of binding site motif with profile *M*_*j*_.

### 7. Statistical evaluation of the predicted binding sites

We evaluated the statistical significance of our predictions by comparing the probability of finding a high scoring binding site in inter-TU sequences with that of finding the same high scoring binding site in randomly extracted coding sequences [32,33]. Let *p*(*S*_*t*_>*s*) be the probability that an extracted sequence *t *(*t *∈ *I*_*U *_or *t *∈ *C*_*U*_) contains a putative binding site with a score (*S*_*t*_) larger than *s*. To avoid possible biased sampling, 300 *C*_*u*_s for each TU in each genome were randomly generated, and *S*_*Cu *_was computed for each *C*_*u*_. Then a log-odd ratio (LOR) function defined as following was used to estimate the confidence of the prediction:

(6) $$LOR(s)=\ln\frac{p(S_{I_{U}}>s)}{p(S_{C_{U}}>s)}.$$

Since $p(S_{C_{U}}>s)$ is the probability of type-I error for testing the null hypothesis that *I*_*u *_does not contain a binding site when $S_{I_{U}}$ is greater than a cutoff *s*, we used it to estimate the false positive rate of the prediction results. In this way, $p(S_{C_{U}}>s)$ could be also considered as an empirical *p-*value, and a cutoff of *p *< 0.01 was used for the CRP binding site prediction in each genome.

## Results

### 1. Conservation of the DBDs of the CRP proteins in cyanobacterial genomes

**Figure 1:**
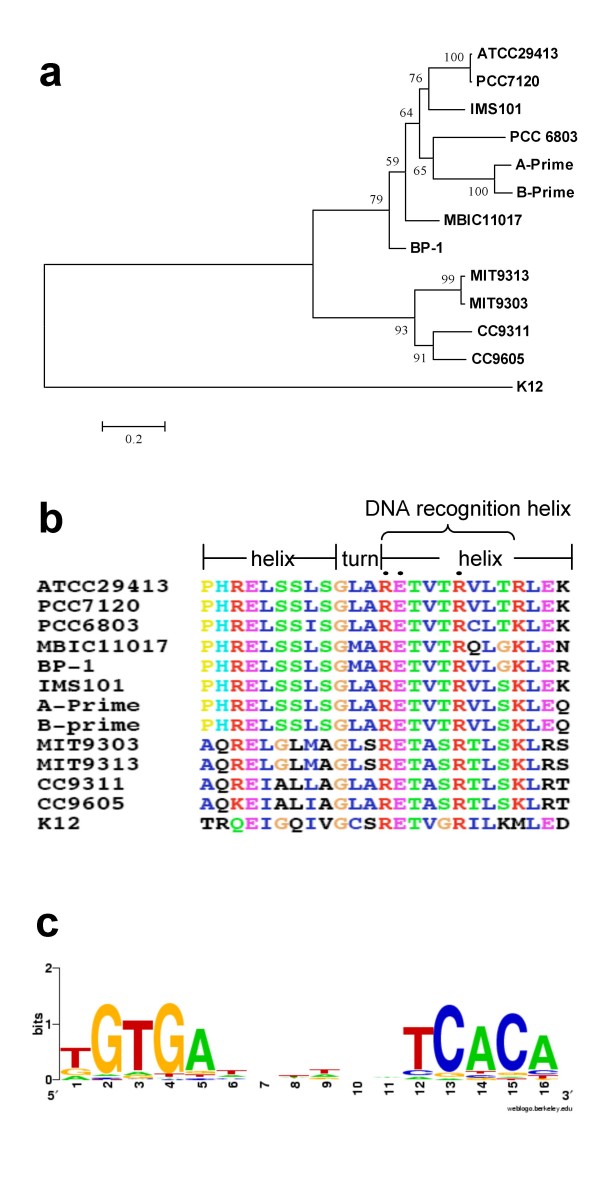
**(a) Phylogenetic relationships of CRPs from 12 cyanobacterial genomes**. The tree is rooted with the CRP of *E. coli *K12, and bootstrap values are shown on the nodes. **(b) **Multiple sequence alignments of the DNA binding domains of the 12 cyanobacterial CRPs. The DNA binding domains contains a helix-turn-helix motif in which residues 1–9 form the first helix, residues 10–12 form the turn and residues 13–25 form the second helix. Particularly, residues 13–21 form the DNA recognition helix with residues 13, 14, 18 (indicated by solid dots) being in direct contact with DNA through forming hydrogen bonds with it [39]. **(c)**. Logo representation of the profile of the 112 putative CRP binding sites predicted by the phylogenetic footprinting technique. Logo was generated by the Weblogo server [49].

Using the BDBH algorithm and the criterion described in Methods, we identified orthologues of SyCRP1 (sll1371) of PCC6803 in 12 of the 29 sequenced cyanobacterial genomes. All these 12 genomes encode one SyCRP1 homologue, with the exception that the PCC6803 and PCC7120 genomes contain two: sll1371 and sll1924 (SyCRP2) in PCC6803, and alr0295 (AnCRPA) and alr2325 (AnCRPB) in PCC7120, which are identified by a unidirectional BLASTP search using SyCRP1 of PCC6803 as the query and an E-value cutoff 10^-20^. The phylogenetic tree of these 12 CRP orthologues shows that they fall into two distinct groups (Figure 1a). There are clear subtle differences between the DBDs of the two groups. Nonetheless, the residues of the DBD that are in direct interaction with the DNA counterpart as revealed by the crystal structure of *E. coli *CRP/DNA complex [39], are highly conserved in all these cyanobacterial CRP sequences, suggesting that they might bind to similar DNA sequences (Figure 1b).

### 2. Profiles of the CRP binding sites predicted by phylogenetic footprinting

When the 18 genes located in the five TUs (Table 1) in PCC6803 that are likely to be regulated by SyCRP1 in this species, are searched against the 12 cyanobacterial genomes that encode *crp *genes, we found a total of 30 orthologues in five genomes. These 30 orthologous genes are located in a total of 10 TUs in these five genomes, indicating that these TUs are not well conserved in these 12 genomes. By using the phylogenetic footprinting techniques (see Methods), we predicted a total of eight putative CRP binding sites from the 10 upstream inter-TU regions, suggesting that the CRP binding sites are largely shared by these orthologous genes. In order to increase the representation of the profile of the CRP binding sites and to minimize the possible bias of our original choice of the five TUs, we performed a one-round iteration of putative CRP binding site scanning using this preliminary profile constructed from these eight putative CRP binding sites (see Methods). From this preliminary whole genome scanning results, we selected a total of 112 putative CRP binding sites (Table S1 in Additional file 2) with high scores to construct the final profile of CRP binding sites. These sites display a strong pseudo-palindromic structure with consensus TGTGAN_6_TCACA (Figure 1c), which is similar to the canonical CRP binding sites in *E. coli*, suggesting that the pattern of CRP binding sites is well conserved between cyanobacterial and *E. coli*. This result is also in agreement with the observation that the binding sites of members of the CRP/FNR superfamily maintain a high level of conservation across difference lineages [40].

### 3. Genome-wide prediction of CRP binding sites in the 12 cyanobacterial genomes

**Figure 2:**
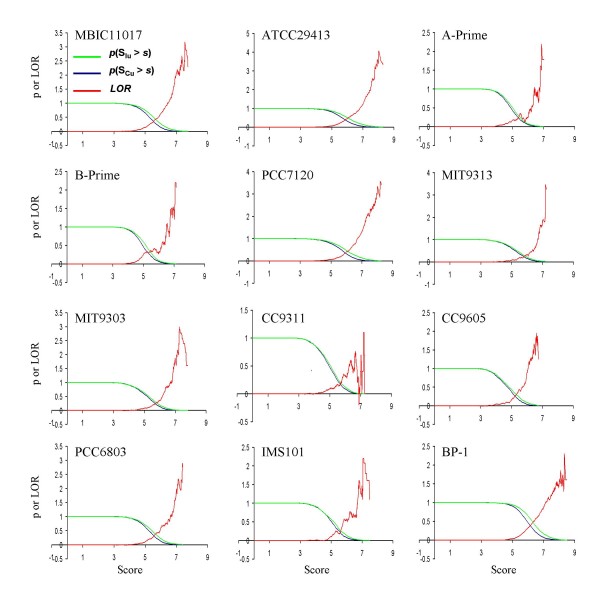
**Evaluation of the genome-wide prediction of CRP regulons in 12 cyanobacterial genomes**. The green curves represent the probability $p(S_{I_{U}}>s)$ and the blue ones represent $p(S_{C_{U}}>s)$. The red curves are the log-odd ratio (LOR), defined as $LOR(s)=\ln(p(S_{I_{U}}>s)/p(S_{C_{U}}>s))$ (see Methods).

We then apply our motif scanning algorithm to predict putative CRP binding sites in the 12 cyanobacterial genomes that encode a *crp *gene using the profile of the 112 putative CRP binding sites as well as that of the previously prepared -10 like box from cyanobacteria [32]. The log-odds ratio (LOR) function of the predictions in each genome is all high when the score *s *is high (Figures 2), suggesting that these genomes are likely to contain functional CRP binding sites. The predictions in each of the 12 genomes at *p*-value < 0.01 are listed in Tables S3-S14 in Additional file 2, and all the prediction results are available upon request. In general, all these predicted CRP promoters contain a pseudo-palindromic CRP binding site and most of them also contain a downstream TA-rich σ-factor binding site, therefore they are likely to be true CRP promoters. As shown in Table S12 (see Additional file 2), the five CRP binding sites associated with the five TUs (shown in bold) in PCC6803, which we selected as the starting point of the our genome-wide prediction, were not necessarily ranked very high (they were ranked 4th, 12th, 14th, 17th, and 43rd) among the 59 predicted CRP binding sites in that genome, suggesting that our initial choice of the five TUs did not bias our prediction to them. Table 2 summarizes the predictions in the 12 cyanobacterial genomes, and the most prevalent genes of predicted CRP regulons are listed in Table S2 in Additional file 2.

**Table 2:** Summary of the genome-wide CRP binding site predictions.

Genome	No. of TU	% genes shared with *E. coli *K12^1^	Score at p < 0.05	LOR at p < 0.05	No. of sites predicted at p < 0.05	Score at p < 0.01	LOR at p < 0.01	No. of sites predicted at p < 0.01
MBIC11017	3406	16.18	6.42	0.96	445	6.85	1.60	174
ATCC29413	3278	19.98	6.95	1.14	521	7.41	1.85	211
A-prime	1429	29.08	6.03	0.32	100	6.4	0.98	41
B-prime	1495	29.20	6.1	0.37	109	6.51	1.08	44
PCC7120	3300	18.56	6.89	1.17	537	7.34	2.01	249
MIT9313	1340	32.19	6.47	0.63	124	6.83	1.55	63
MIT9303	1786	24.62	6.34	0.69	186	6.81	1.10	55
CC9311	1656	26.89	6.22	0.43	130	6.65	0.66	34
CC9605	1391	28.42	5.93	0.67	237	6.37	1.31	90
PCC6803	1622	27.14	6.34	0.76	181	6.82	1.19	59
IMS101	3253	19.34	7.13	1.01	451	7.68	1.40	132
BP-1	1075	31.41	6.16	0.62	101	6.68	0.93	29

1. Calculated as the number of genes that have an orthologue in the *E. coli *K12 genome normalized to the total number of genes in that genome.

Although it has been estimated that CRP controls the expression of more than 200 TUs in *E. coli *[2], and the RegulonDB (release 5.8) contains 288 experimentally verified CRP binding sites, the number of predicted CRP binding sites at *p*-value < 0.01 in each cyanobacterial genomes is relatively small, ranging from 29 in BP-1 (containing 1075 putative TUs) to 249 in ATCC29413 (containing 3300 putative TUs), if one considers that the *E. coli *K12 genome encodes a total of 2070 putative TUs (predicted by the algorithm described in Methods), and that some of these cyanobacterial genomes contain much more TUs/genes (Table 2) than the *E. coli *K12 genome does. This result suggests that cyanobacterial CRPs might regulate fewer genes than the *E. coli *CRP does. We then ask whether the target genes of CRPs are conserved between *E. coli *and cyanobacteria, as well as among the 12 cyanobacterial genomes, given that the DBDs of CRPs as well as their binding sites are highly conserved.

**Figure 3:**
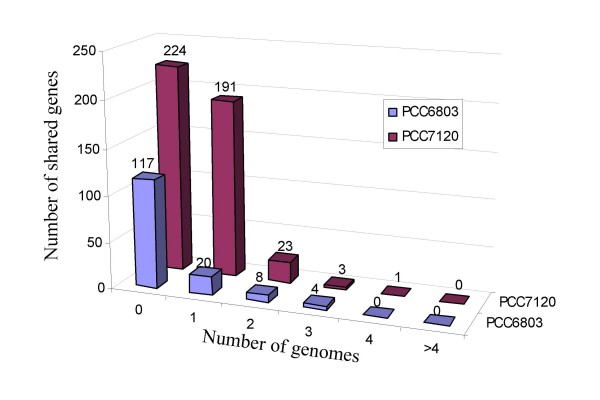
**Distribution of shared putative CRP-regulated genes in sequenced cyanobacterial genomes**. Only two representative examples from PCC6803 and PCC7120 are shown for clarity. The horizontal axis is the number of genomes that share putative CRP-regulated genes with PCC6803 or PCC7120, and the vertical axis is the number of shared genes. Among the 149 predicted CRP-regulated genes (at *p *< 0.01) in PCC6803, 117 (75%) are species specific, and the rest 32 (25%) are shared with other genomes; of the 442 predicted CRP-regulated genes (at p < 0.01) in PCC7120, 224 (50.7%) are species specific, and the rest 318 (49.3%) are shared with other genomes, most of which (191) are shared with the closely related ATCC29413 genome.

Although these 12 cyanobacterial genomes share 13.88~32.19% of their genes with *E. coli *(Table 2), they almost have no common genes in their CRP regulons with that of *E. coli *K12 (Tables 3, and S3-S14 in Additional file 2), suggesting that the CRPs in cyanobacteria have adapted to regulate very different sets of genes than those in *E. coli *during the course of evolution. On the other hand, the majority of the CRP targets in these cyanobacterial genomes are not conserved either, rather, only a small portion of the CRP targets are shared by more than 2 of the 12 cyanobacterial genomes (Figure 3), even though our motif scanning algorithm might be biased toward the genes that have orthologues in reference genomes (see Methods). In the extreme cases, many CRP targets are species or lineage specific, suggesting that the targets of CRP have changed very rapidly since the speciation of these cyanobacterial genomes. For example, only 32 out of the 149 putative CRP target genes in the PCC6803 genome are conserved in at least 2 of the 12 cyanobacterial genomes. The only exception is the PCC7120 genome in which the number is 218 out of 442. However, this is largely because the reference genomes include a closely related species ATCC29413. Moreover, the most conserved putative CRP-regulated genes are only shared by five genomes (Figure 3). In addition, 17 of the 29 sequenced cyanobacterial genome do not encode the *crp *gene, suggesting that CRP is not required for the life of these 17 organisms; alternatively, the function of CRP in these organisms have been replaced by another TF.

**Table 3:** The CRP regulons are not conserved between PCC6803, PCC7120 and *E. coli *K12.

	*E. coli *K12	PCC6803	PCC7120
No. of genes	4133	5366	3172
No. of TUs	2070	1622	3300
No. of CRP-regulated genes	410	382 (p < 0.05),149 (p < 0.01)	969 (p < 0.05),442 (p < 0.01)
No. of CRP-regulated TUs	270	181 (p < 0.05),59 (p < 0.01)	537 (p < 0.05),249 (p < 0.01)
No. of CRP-regulated genes shared with *E. coli *K12	--	7 (p < 0.05), 2 (p < 0.01)	17 (p < 0.05),6 (p < 0.01)

### 4. Functional classification of CRP regulons in cyanobacteria

Based on the functional annotations of the CRP target genes that we have predicted in this study, CRP seems to be involved in a rather diverse spectrum of functions in cyanobacteria (Table 4) as summarized below.

**Table 4:** Putative CRP-regulated genes involved in different biological processes.

Genome	Photosynthesis and carbon fixation	Carbon metabolism	Nitrogen assimilation	Transporters/porins	Kinases	Transcription factors
MBIC11017	*AM1_1272* *AM1_1560* *AM1_0526*	*AM1_2193* *AM1_2114*	*AM1_2462* *AM1_5490* *AM1_0481*	*AM1_1165* *AM1_6038* *AM1_0773* *AM1_2986* *AM1_3534* *AM1_4901* *AM1_2335*	*AM1_5208* *AM1_5107* *AM1_2792* *AM1_5169*	*AM1_3844* *AM1_4185* *AM1_5140* *AM1_0632*
ATCC29413	*Ava_4451* *Ava_3710* *Ava_0640*	*Ava_1491*	*Ava_4669*	*Ava_0687* *Ava_4995* *Ava_1172* *Ava_0874*	*Ava_4457* *Ava_0873* *Ava_0613* *Ava_4753* *Ava_1559* *Ava_3542* *Ava_1149* *Ava_3207* *Ava_3867* *Ava_4503* *Ava_3009*	*Ava_3703* *Ava_1629* *Ava_2629* *Ava_1558* *Ava_1021*
A-prime	*CYA_0295*			*CYA_2315*		
B-prime	*CYB_2824*				*CYB_0465* *CYB_2795*	
PCC7120	*asr0847* *alr0317 alr0318* *alr0523 alr0524 alr0525*	*alr0169*	*alr0874* *all3335 all3334 all3333 all3332*	*alr2210 alr2211 alr2212 alr2213* *alr2118 alr2119 asr2220* *all3335* *alr3938*	*all0853* *alr3037* *alr1192* *all1191* *all3207* *alr0428* *all4668* *alr1665* *alr3268* *alr2137* *all0323* *all3767* *all2883*	*alr1044* *all0187* *all2962* *alr1941*
MIT9313	*PMT1665*			*PMT2110* *PMT1524*	*PMT0265* *PMT0845*	*PMT0986*
MIT9303	*P9303_22121* *P9303_22711*			*P9303_04171*		*P9303_17661* *P9303_11401*
CC9311				*sync_0219*		
CC9605	*Syncc9605_1640* *Syncc9605_0485*			*Syncc9605_1575* *Syncc9605_2642*		*Syncc9605_2284*
PCC6803	*sll1577 sll1578 sll1579 sll1580 ssl3093* *sll0634* *slr1739* *sll1874 sll1875* *slr1459* *slr1838 slr1839 slr1838*	*sll1709* *slr2082 slr2083* *ssl2153* *sll0084* *slr1349* *slr1350*		*slr1392* *sll0537* *sll0240* *slr1452 slr1453 slr1454 slr1455 slr1457 slr1453* *slr1488* *sll0273* *slr1950*	*slr1400* *slr1805* *slr0484*	*sll1708* *sll1371* *slr1489*
IMS101	*Tery_4669*			*Tery_2879* *Tery_1324* *Tery_4986* *Tery_3858* *Tery_0199* *Tery_2779*	*Tery_1627* *Tery_3423* *Tery_2051* *Tery_4912*	*Tery_1557*
BP-1	*tsr0033*	*tlr1171*	*tll0330* *tlr2000 tlr2001 tlr2002 tlr2003*	*tll0559* *tlr0335* *tlr2000 tlr2001 tlr2002 tlr2003*		*tll0328*

#### 4.1. Photosynthesis and carbon fixation

Various numbers of genes involved in photosynthesis and carbon fixation were predicted to bear a CRP-regulated promoter in the 12 cyanobacterial genomes that encode *crp *genes. Specifically, a total of 14 (at *p *< 0.01) photosystem I and II reaction center genes were predicted to be regulated by CRP, including *AM1_0526, asr0847, Ava_4451, Ava_0640, asr0847, CYA_0295, CYB_2824, PMT1665, P9303_22121, P9303_22711, Syncc9605_1640, sll0634, slr1739*, and *Tery_4669*. Several carbon dioxide concentrating mechanism protein ccmK genes, including *alr0317-0318 *and *slr1838-slr1839*, were also predicted to bear a CRP binding site. Moreover, putative CRP-regulated promoters were found for the phycobiliprotein family light-harvesting genes, including *alr0523-0525, sll1577-1580, ssl3093, slr1459 *and *tsr0033*. In consistent with these results, it has been previously shown that cellular cAMP levels change significantly in response to environmental stimuli such as light-dark cycle [41]. Thus cyanobacterial CRPs are likely to participate in photosynthesis pathways.

#### 4.2. Nitrogen assimilation

Three nitrogenase related proteins AM1_2462, Ava_4669 and alr0874 in MBIC11017, ATCC29413, and PCC7120, respectively, were predicted to be CRP-regulated. Furthermore, an operon encoding a nitrate transporter (all3332-3335) in PCC7120 was predicted to bear a CRP binding site. It has been previously reported that nitrogen starvation resulted in a 3–4-fold increase in intracellular cAMP level in *Anabaena variabilis *[42,43]. Based on our prediction results, a possible scenario for a role of CRP in the signaling pathway of nitrogen assimilation could be as follows. Nitrogen starvation somehow increases the adenylyl cyclase activity, leading to an increase in the intracellular cAMP level. The activation of CRP by cAMP then lowers the transcription level of genes such as nitrate transporter (*all3332-3335*), while it enhances the expression of genes like *alr0874 *(nitrogenase reductase) and nitrogenase (*AM1_2462, Ava_4669*), as the cell switches to the more energy intensive nitrogen fixation of nitrogen gas. Nonetheless, an in-depth study is needed to elucidate the details of the role that CRP may play in nitrogen fixation in these cyanobacteria. Since not all cyanobacterial species are capable of nitrogen fixation, this role of CRP is unique to the cyanobacterial species capable of nitrogen fixation, such as MBIC11017, ATCC29413, and PCC7120.

#### 4.3. Transporters and porins

A few genes coding for transporters and porins were predicted to be CRP-regulated, including several ion transporter in PCC7120 (*alr2210-2213 *and *alr2118-2120*) and PCC6803 (*slr1392 *and *slr1950*). Besides, several antiporters and ABC transporters were also predicted to be CRP-regulated in various genomes, e.g. CYA_2315, Ava_0687, sll0240, slr1452-1457, tll0559.

#### 4.4. Kinases and two-component signal transduction systems

Dozens of genes coding for kinases and two-component signal transduction systems were predicted to be CRP-regulated, suggesting that CRP might play an important role in response to environmental changes in cyanobacteria. Interestingly, it has been reported that the CRPs in PCC6803 are involved in phototaxis as both *sycrp1 *and adenylyl cyclase mutants showed impaired phototaxis [9,29]. However, the genes that are involved in signal transduction for pilus assembly and phototaxis, as listed in [29], were not predicted to be CRP-regulated by our algorithm, therefore, they might be regulated by CRP indirectly.

#### 4.5. Other functions unique to a species/lineage

Among the top hits of our prediction results, a large portion of putative CRP-regulated genes are species or lineage specific. The functions of these genes vary from genome to genome, such as the type IV pilus synthesis in PCC6803 (*slr1667-1668 *and *slr2015-2018*) and MBIC11017 (*AM1_3323-3324*); various transposase in PCC7120 (*all3624*), BP-1 (*tll2385*) and IMS101 (*Tery_0925*); methyltransferase in MBIC11017 (*AM1_5474*); aldo/keto reductase in A/B-Prime (*CYA_0976 *and *CYB_2928*); nblA in BP-1 (*tsr0033*); TPR repeat containing protein in ATCC29413 (*Ava*_*3483*); peptidase in MIT9313 (*PMT1940*); nuclease in CC9311 *(sync_1258*), etc. However, the functions of many other species or lineage specific putative CRP-regulated genes are largely unknown, most of them are annotated as hypothetical proteins, such as *slr0442*, *sll1268*, *ssr2848 *and *sll1924 *in PCC6803, *asr4669 *in PCC7120, *AM1*_*3950*-*3951*, *AM1*_*4103*, *AM1_4957 *and *AM1_2209-2210 *in MBIC11017, *CYA_0127/CYB_2776 *in A/B-Prime, *Tery_2530 *and *Tery_1044 *in IMS101, *Ava_3757 *in ATCC29413, *PMT1492*, and *PMT1223 *in MIT9313, *P9303*_*06191*, *P9303*_*04111 *and *P9303*_*12031 *in MIT9303, *sync_093*, and *sync_1261 *in CC9311, and *Syncc9605_0955 *and *Syncc9605_0452 *in CC9605. It would be interesting to experimentally characterize the functions of these genes as well as the roles that CRP plays in their transcriptional regulation.

### 5. crp genes were lost in some cyanobacterial genomes during the course of evolution

**Figure 4:**
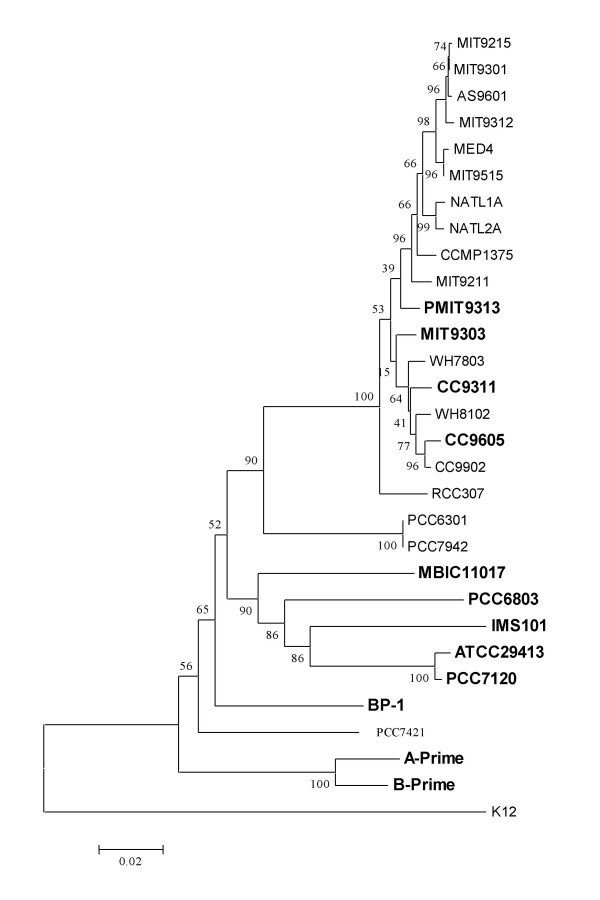
**Relationships between the 29 sequenced cyanobacterial species/strains inferred from their 16S rRNA genes**. The tree is rooted with the 16S rRNA gene of *E. coli *K12. Bootstrap values are shown on the nodes. The cyanobacterial species/strains encoding a *crp *gene are indicated by bold font.

The presence of *crp *genes in some, but not all cyanobacterial genomes, raised the question about their evolutionary origin: are *crp *genes in cyanobacteria acquired through horizontal gene transfer (HGT) from other species, or are they vertically inherited from their last common ancestor, but lost in some cyanobacterial species/strains? To address this question, we constructed a species tree of the 29 sequenced cyanobacterial genomes based on their 16S rRNA gene sequences (Figure 4). Although some nodes in the tree are not well supported by the currently available sequence data, the relationships of the species in the tree are in excellent agreement with a previously inferred cyanobacterial species tree [44]. As shown in Figure 4, the genomes encoding a *crp *gene do not form a particular monophyletic group, rather, they are sporadically scattered across the tree. On the other hand, all the known cyanobacterial CRPs form a monophyletic group (Figure S1 in Additional file 1), suggesting that they are likely derived from a common ancestor. Therefore, the current sporadic distribution of the *crp *genes within the sequenced cyanobacteria likely resulted from differential gene losses during the course of evolution. Independent acquisition of the *crp *gene by individual cyanobacterial lineage/species, though theoretically possible, is less parsimonious since it would entail multiple HGT events from the same or closely related donors. Therefore, we conclude that the 17 cyanobacterial genomes that do not encode a *crp *gene actually lost their original ones during the course of evolution to adapt to their respective environments. It is interesting to note that most of the species lacking a *crp *gene belong to marine cyanobacteria; these species often have a reduced genome and inhabit a relative stable oligotrophic environment. On the other hand, the species that contain a *crp *gene are distributed in both fresh water and terrestrial environment. This again suggests that the *crp gene *might be beneficial to the species that live in a variable environment, and those that have adapted to a more stable environment such as oligotrophic ocean lost their *crp *genes.

### 6. Degradation of CRP binding sites in cyanobacterial genomes after the crp genes were lost

We have previously shown that when a genome lost a TF in the course of evolution, then it would rapidly lose its cognate biding sites in inter-TU regions [32,33]. To extend this conclusion, we applied our CRP binding site prediction algorithm to the 17 cyanobacterial genomes that do not encode a CRP orthologue. Indeed, in four genomes, namely, CC9902, RCC307, WH8102 and WH7803 (Figures S2 in Additional file 3), the LOR function oscillates around zero as the score *s *increases, indicating that there is no significant difference between the signal of CRP binding sites in the inter-TU regions and that in the randomly selected coding regions (see Methods). These results strongly suggest that these four genomes are unlikely to contain functional CRP binding sites, which is in agreement with our previous observation [32,33]. However, there is a clear CRP binding site signal in the rest of 13 genomes as indicated by their relatively high LOR when the score *s *is high, suggesting that there exist CRP-like binding sites in the inter-TU regions in these genomes. The reason for this unexpected observation is unknown, but one explanation would be that these sites are bound by a different regulator in these genomes. To identify possible TFs in these genomes that are likely bind these CRP-like binding sites, we analyze the distribution of the CRP/FNR superfamily in these genomes, and found that they all encode at least one member of the superfamily, which are likely to recognize these CRP-like binding sites, as it has been shown that members of the CRP/FRN superfamily recognize similar consensus sequence, and the binding specificity is achieved through competitive binding among the members.

## Discussion

### 1. CRP is a lineage-specific global regulator

Studies have shown that CRP in *E. coli *functions as an important global regulator controlling the expression of genes involved in many pathways such as the carbon and energy metabolism pathways. It was also reported that CRP regulates a variety of genes in other bacterial species. For instance, one recent study showed that CRP-like protein regulates genes involved in quorum sensing, motility and intestinal colonization in *Vibrio cholerae *[7]. In this study, we suggest that CRP in cyanobacteria seems to have distinct functions. First, our results show that the members of the CRP regulons in cyanobacteria have little in common with those in *E. coli *(Table 3), which is consistent with the observation that genes whose expression is mostly affected by *sycrp1 *disruption in PCC6803 are involved in the type IV pilus synthesis [8,25,29]. In contrast, genes that are involved in carbon and energy metabolisms as seen in *E. coli *are not significantly affected by *sycrp1 *disruption [8,25,29]. Second, cyanobacterial CRPs seem to regulate distinct sets of genes specific to each lineage or strain as we can not clearly identify a particular set of genes common to most of the 12 cyanobacterial genomes that are under CRP control (Figure 3, Table S2 in Additional file 2). Third, more than half (17) of the 29 completely sequenced cyanobacterial genomes do not encode a CRP orthologue, suggesting that CRP is dispensable in these strains/species. For a closely related group of species, it is unlikely that an essential global regulator can be replaced or lost in some genomes while present in the others. Therefore, we conclude that CRP is not a global regulator with conserved functions; instead, it is likely a lineage-specific global regulator.

In fact, it has been shown that global regulators are not necessarily conserved in moderately related species. For instance, among the 7 and 6 global regulators in *E. coli *and *B. subtilis*, respectively, none of them is in common [11]. Furthermore, it is not surprising that CRP in cyanobacteria does not function as a conserved global regulator as seen in *E. coli*, given that CRP is mainly involved in the transcriptional regulation of genes related to carbon metabolism in *E. coli*, while organic carbon assimilation is no longer a constraint for the growth of autotrophic cyanobacteria. On the other hand, since nitrogen assimilation is a constraint for cyanobacteria, the nitrogen assimilation regulator, NtcA, a member of the CRP/FNR family, which is unanimously encode in all the 29 sequenced genomes, has been characterized as one of the conserved global regulators in cyanobacteria. Thus, it seems that global regulators are often lineage-specific and that the environment plays a vital role in determining which TF functions as a global regulator, and which genes are regulated by the TF.

### 2. The functions of CRP in cyanobacteria are highly diverse

It has been reported that SyCRP1 regulates the cellular motility in PCC6803, as *sycrp1 *disruptants were devoid of mobility and showed reduced type IV pilus biogenesis [8]. In another study, it was shown that the operons *slr1667-slr1668 *and *slr2015-slr2018 *in PCC6803, which are involved in type IV pilus biosynthesis [29,45], were down-regulated in *sycrp1 *disruptants using microarray gene expression profiling [25]. In consistent with these findings, we have predicted putative CRP binding sites for these genes. However, the *slr1667-slr1668 *genes were unique to PCC6803, and the orthologues of *slr2015-slr2018 *could only be found in MBIC11017 (Table S2 in Additional file 2) among the 29 cyanobacterial genomes. Thus, this function of CRP is likely to be restricted to these two species/strains only. In addition, based on our predictions of CRP regulons in the 12 cyanobacterial genomes, we argue that CRP might be also involved in other functions in different cyanobacterial lineage/strains, including carbon fixation, photosynthesis, nitrogen fixation, ion channels/transporters, and two-component signal transduction, etc. (Table 4, Table S3 – S14 in Additional file 2). Furthermore, as a large portion of our predicted CRP binding sites are associated with hypothetical proteins, CRP might be involved in the regulation of the other novel functions yet to be discovered. In this regard, we have provided a set of candidates for further experimental characterization. However, due to the lack of sufficient information about the functions of the CRP target genes, it is currently rather difficult to derive a general pathway model involving CRP regulons in cyanobacteria. Lastly, AnCrpA was shown to regulate the expression of genes related to nitrogen fixation in PCC7120 [28], including *all1517 *(*nifB*), *all1439*, *all1432 *(*hesA*), *alr2515 *(*coax-II*), and *alr2834 *(*hepC*). However, our algorithm failed to find high scoring CRP binding sites for all these genes, the possible reasons for this are addressed below.

### 3. Binding sites of AnCRPA in Anabaena sp. PCC7120

Our failure to identify high scoring CRP binding sites in the upstream regions of these nitrogen fixation genes in PCC7120 is actually in agreement with the finding by Suzuki and coworkers who suggested that AnCrpA might have a different binding site pattern from the conventional pseudo-palindromic motif [28]. However, an *in vitro *binding affinity test using EMSA showed that AnCrpA could bind to the conventional palindromic motif [27]. Thus, a possible explanation of this inconsistency would be that AnCrpA in PCC7120 could form a monomer or a heterodimer in addition to a homodimer, given that two CRP homologues are encoded in this genome, and one of them does not have a DBD. Such a monomer or heterodimer might favor a non-canonical CRP binding site, while the homodimer remains its ability to bind to the conventional palindromic motif. Clearly, a more in-depth study on this topic is needed to verify this hypothesis. This explanation is in agreement with the results that our algorithm predicts many CRP binding sites for other genes in PCC7120 with statistical significance (Table S7 in Additional file 2).

### 4. Origin of crp genes in cyanobacteria and implications for the evolution of CRP regulatory networks in bacteria

Because the *crp *gene is widely distributed in many distantly related bacterial groups, including actinobacteria, aquificales, bacteroidetes, chlamydiae, chloroflexi, cyanobacteria, deinococci, firmicutes, fibrobacteres, planctomycetes, proteobacteria, spirochaetes, etc. (Figure S1 in Additional file 1), it was likely present in the common ancestor of the extant eubacterial lineages. Under this scenario, the *crp *gene in this ancestor was flexible enough to regulate different sets of genes. Alternatively, it is also possible that the *crp *gene originally evolved in a specific bacterial lineage and was subsequently spread to other groups via HGT. Such HGT events may benefit the recipient organism given the flexibility of CRP in regulating different biochemical activities, which are often coupled to environment stimuli leading to the generation of intracellular signaling molecule cAMP. Therefore, the *crp *gene in the ancestor of modern cyanobacteria was acquired by either vertical inheritance or an ancient HGT event from other lineages. Some cyanobacteria lost their *crp *genes since harboring the gene might not necessarily increase their fitness in their new environments. On the other hand, in other cyanobacteria, the *crp *genes were adapted to better meet their unique physiology and environmental requirements. In other groups of bacteria, *crp *evolved to regulate other lineage/species specific functions. For instance, it regulates the carbon and energy metabolisms in *E. coli*, cell-cell communication in *Stenotrophomonas maltophilia *[46], and quorum sensing in *Vibrio cholerae *[7], etc

### 5. Rapid degradation of CRP binding sites

Evolution of *cis*-regulatory binding sites is an interesting, but not well-studied problem. The 17 cyanobacterial genomes that do not encode a CRP orthologue provided us an excellent opportunity to examine the degradation of the CRP binding site in these genomes. It was expected that high scoring CRP binding sites do not present in those genomes, as previous studies have indicated that binding sites rapidly fade out when the corresponding TF was lost during the course of evolution [32,47]. We do see such fading in the CC9902, RCC307, WH7803, and WH8102 genomes (Figure S2 in Additional file 3). These observations were in consistent with the well-accepted rule that "if no such a TF in a genome, then no corresponding binding sites in the genome". However, surprisingly, in the rest 13 genomes, high scoring CRP-like binding sites seem to appear in the inter-TU regions with a higher probability than in the randomly selected coding regions (Figure S2 in Additional file 3), suggesting that there exist sequence patterns similar to CRP binding sites in these cyanobacterial genomes. A possible explanation of this unexpected observation would be that there exist in these genome TFs that recognize binding sites similar to CRP binding sites. Indeed, at least one member of the CRP/FNR superfamily is encoded in these 13 genomes, and it has been shown that the binding sites of the members of this TF superfamily are well conserved across many species with a wide range of evolutionary distance [40]. Thus, the CRP-like binding sites found in these genomes are likely to be recognized by these non-CRP TFs of the superfamily. The specificity of these similar binding sites is likely to be achieved through the competition among homologous TFs for the same binding site, which is governed by their thermodynamic equilibrium [48].

## Conclusion

In this paper, we have predicted CRP binding sites in 12 cyanobacteria genomes that encode a CRP orthologue using a highly accurate motif scanning algorithm. Based on the analysis of these predictions as well as experimental data available to us, we conclude that 1) CRP has rather different functions in cyanobacteria than in *E. coli*; 2) cyanobacterial CRP also has a very diverse spectrum of functions in different lineages or species/stains, and is even dispensable in some species/strain; 3) CRPs in modern cyanobacteria are likely to be vertically inherited from their last common ancestor, and some cyanobacteria lost their *crp *genes during the course of evolution to adapt to their new environments; and 4) once the *crp *gene is lost, its binding sites degrade rapidly. Although many of our predictions still await experimental verification, we should have provided a high quality candidate set for further experimental characterization of the CRP binding sites and regulons in this important group of bacteria.

## Abbreviations

ORF: Open reading frame; TU: Transcription unit; BDBH: bidirectional best hit; CRP: cAMP receptor protein; DBD: DNA binding domain; HTH: helix-turn-helix; EMSA: electrophoresis mobility shift assay; TF: transcription factor; HGT: horizontal gene transfer; LOR: log-odd ratio; MBIC11017: *Acaryochloris marina *MBIC11017; ATCC29413: *Anabaena variabilis *ATCC 29413; A-prime: *Synechococcus sp*. JA-3-3Ab; B-prime: *Synechococcus sp*. JA-2-3B'a(2-13); PCC7421: *Gloeobacter violaceus *PCC 7421; PCC7120: *Nostoc sp*. PCC 7120; CCMP1375: *Prochlorococcus marinus *CCMP1375; MED4: *Prochlorococcus marinus *MED4; AS9601: *Prochlorococcus marinus *AS9601; MIT9211: *Prochlorococcus marinus *MIT 9211; MIT9215: *Prochlorococcus marinus *MIT 9215; MIT9301: *Prochlorococcus marinus *MIT 9301; MIT9312: *Prochlorococcus marinus *MIT 9312; MIT9303: *Prochlorococcus marinus *MIT9303; MIT9313: *Prochlorococcus marinus *MIT9313; MIT9515: *Prochlorococcus marinus *MIT 9515; NATL1A: *Prochlorococcus marinus *NATL1A; NATL2A: *Prochlorococcus marinus *NATL2A; PCC7942: *Synechococcus elongatus *PCC 7942; PCC6301: *Synechococcus elongatus *PCC 6301; RCC307: *Synechococcus sp*. RCC307; WH7803: *Synechococcus sp*. WH 7803; WH8102: *Synechococcus sp*. WH8102; CC9605: *Synechococcus sp*. CC9605; CC9902: *Synechococcus sp*. CC9902; CC9311: *Synechococcus sp*. CC9311; PCC6803: *Synechocystis sp*. PCC 6803; BF-1: *Thermosynechococcus elongates BP-1*; and IMS101: *Trichodesmium erythraeum *IMS101.

## Authors' contributions

M.X. designed and conducted the experiments, and wrote the manuscript. Z.S. conceived the project and wrote the manuscript. All authors read and approved the final manuscript.

## Supplementary Material

Additional File 1 **Figure S1**. A protein tree containing 202 SyCRP1 orthologues from a wide range of bacterial species. The red-labeled branch is CRPs from cyanobacteria. Click here for file

Additional File 2 **Table S1**. Putative CRP binding sites used to construct the profile of CRP binding sites. **Table S2**. Most conserved putative CRP-regulated genes/TUs in 12 cyanobacterial genomes. **Table S3–14**. Predicted CRP binding sites in 12 cyanobacterial genome at *p*-value < 0.01. Click here for file

Additional File 3 **Figure S2**. Genome-wide scanning for CRP-like binding sites in the 17 genomes that do not encode a CRP protein. Click here for file
