# Supplementary material for: Computational prediction of cAMP receptor protein (CRP) binding sites in cyanobacterial genomes
Source: BMC Genomics. 2009 Jan 15;10:23. doi: 10.1186/1471-2164-10-23 (PMC2633013; doi:10.1186/1471-2164-10-23)
Supplement: Additional File 2 — Table S1. Putative CRP binding sites used to construct the profile of CRP binding sites. Table S2. Most conserved putative CRP-regulated genes/TUs in 12 cyanobacterial genomes. Table S3–14. Predicted CRP binding sites in 12 cyanobacterial genome at p-value < 0.01. [file 1471-2164-10-23-S2.doc]

**Table S1**. 112 Putative CRP binding sites used to construct the CRP binding sites motif profile.

| Genome | Rank^1^ | Transcription unit | Names | putative CRP binding sites | Position^2^ | Score^3^ |
| --- | --- | --- | --- | --- | --- | --- |
| MBIC11017 | 1 | AM1_3321 | - | TGTGAtctagaTCACC | -652 | 8.43 |
| MBIC11017 | 3 | AM1_2586 | - | GGTGAccagacTCACT | -133 | 8.16 |
| MBIC11017 | 4 | AM1_4906 | - | AGTGAcctggaTCACT | -193 | 8.15 |
| MBIC11017 | 5 | AM1_2210 | - | TGTGAtttgagTCACT | -204 | 8.02 |
| MBIC11017 | 6 | AM1_5560 | - | TGTGAgattcaTCACT | -118 | 7.93 |
| MBIC11017 | 7 | AM1_2924 AM1_2925 | - - | AGTGAtttaccTCACA | -156 | 7.89 |
| MBIC11017 | 8 | AM1_5474 | - | GGTGAtgggcaTCACA | -481 | 7.86 |
| MBIC11017 | 9 | AM1_1025 AM1_1026 AM1_1027 AM1_1028 AM1_1029 | - - - - - | TGTGAtcttccACACA | -103 | 7.80 |
| MBIC11017 | 10 | AM1_2336 | - | AGTGAtgtcaaTCACG | -71 | 7.79 |
| MBIC11017 | 11 | AM1_4101 | - | TGTGActtagaTCACA | -173 | 7.76 |
| MBIC11017 | 15 | AM1_1024 AM1_1023 | - - | TGTGTggaagaTCACA | -50 | 7.71 |
| MBIC11017 | 22 | AM1_6038 AM1_6039 | - - | TGGGAtcgtccTCACA | -78 | 7.61 |
| MBIC11017 | 32 | AM1_1753 AM1_1752 | ksgA ispE | AGTGAtccgaaTCACA | -154 | 7.53 |
| MBIC11017 | 39 | AM1_3950 AM1_3951 | - - | TGTGActagtaTCACA | -272 | 7.45 |
| ATCC_29413 | 1 | Ava_4457 Ava_4456 | - - | TGTGAtttgagTCACA | -362 | 9.24 |
| ATCC_29413 | 3 | Ava_0186 | - | GGTGAtacaagTCACA | -309 | 9.23 |
| ATCC_29413 | 4 | Ava_4877 | - | TGTGAtacagaTCACA | -109 | 9.22 |
| ATCC_29413 | 7 | Ava_1278 | - | TGTGAtctaaaTCACT | -82 | 8.76 |
| ATCC_29413 | 8 | Ava_1249 | - | TGTGCtggtatTCACA | -100 | 8.73 |
| ATCC_29413 | 11 | Ava_1279 | - | AGTGAtttagaTCACA | -377 | 8.66 |
| ATCC_29413 | 13 | Ava_0223 | - | TGTGAttaactCCACT | -426 | 8.60 |
| ATCC_29413 | 22 | Ava_4108 | - | AGTGCataattTCACG | -695 | 8.49 |
| ATCC_29413 | 31 | Ava_4669 | - | GGTGTtgtcaaTCACA | -273 | 8.33 |
| ATCC_29413 | 34 | Ava_2212 | - | TGAGTtgttcaACACA | -45 | 8.30 |
| Nostoc_sp | 1 | all0853 all0852 | - - | TGTGAtttgagTCACA | -362 | 9.24 |
| Nostoc_sp | 2 | alr0208 | - | GGTGTtgttttTCACC | -195 | 9.23 |
| Nostoc_sp | 3 | asr2365 alr2366 | - - | GGTGAtacaagTCACA | -98 | 9.23 |
| Nostoc_sp | 4 | asr3217 asr3218 | - - | TGTGAtccaaaTCACA | -109 | 9.22 |
| Nostoc_sp | 5 | all4297 | - | TGTGCtgttatTCACA | -100 | 8.80 |
| Nostoc_sp | 7 | asl4328 | - | TGTGAtctaaaTCACT | -82 | 8.75 |
| Nostoc_sp | 8 | alr1192 | - | AGTGAcaactgACACG | -174 | 8.69 |
| Nostoc_sp | 12 | all2415 | - | TGTGAttaactCCACT | -301 | 8.63 |
| Nostoc_sp | 15 | all4073 | - | TGTGAaccttgACACC | -111 | 8.55 |
| Nostoc_sp | 20 | all1219 | - | GGTGAcatcatTCACA | -137 | 8.49 |
| Nostoc_sp | 29 | alr3608 | - | GGTGAaattttTCACC | -150 | 8.37 |
| Nostoc_sp | 32 | all0122 | - | AGTGCgatcgcTCACC | -59 | 8.32 |
| Nostoc_sp | 35 | all2573 | - | AGTGCtttaaaACACA | -626 | 8.30 |
| PCC6803 | 1 | ssr2333 slr1392 | - feoB | AGTGAttatacTCACA | -86 | 8.65 |
| PCC6803 | 2 | sll1543 | - | TGTGAcccagaTCACA | -181 | 8.30 |
| PCC6803 | 3 | sll1247 | - | TGTGAtctagaTCACC | -151 | 8.22 |
| PCC6803 | 4 | slr1351 | murF | GGTGAtctagaTCACA | -87 | 8.01 |
| PCC6803 | 5 | slr2015 slr2016 slr2017 slr2018 | - - - - | GGTGTttattgTCACA | -346 | 7.98 |
| PCC6803 | 8 | sll1874 sll1875 | AT103 ho2 | TGTGAgaataaTCACA | -387 | 7.62 |
| PCC6803 | 12 | sll1520 sll1521 sll1522 | recN - pgsA | TGTGAtctggaTCACA | -561 | 7.52 |
| PCC6803 | 13 | sll0536 sll0537 | - amt3 | AGTGTccttcgCCACA | -89 | 7.52 |
| PCC6803 | 14 | slr1667 slr1668 ssr2786 | - - - | TGTGAtctgggTCACA | -245 | 7.45 |
| PCC6803 | 15 | slr0442 | - | TGTGAtccagaTCACA | -189 | 7.45 |
| PCC6803 | 17 | sll1268 | - | TGTGAtctagaTCACA | -146 | 7.43 |
| BP-1 | 2 | tll0332 tll0331 tll0330 tll0329 tll0328 | - pdxH ureB - - | TGTGActcagaTCACC | -200 | 7.67 |
| BP-1 | 3 | tlr1736 | - | GGTGAgattggGCACA | -56 | 7.63 |
| BP-1 | 4 | tlr1235 | - | TGTGAtcaatgTCACA | -177 | 7.59 |
| BP-1 | 5 | tlr0333 | - | GGTGAtctgagTCACA | -303 | 7.56 |
| BP-1 | 6 | tll0251 | - | GGTGTgccagtTCACA | -64 | 7.55 |
| IMS101 | 1 | Tery_2466 | - | AGTGTatatcaTCACA | -174 | 8.75 |
| IMS101 | 2 | Tery_3081 Tery_3082 Tery_3084 | - - - | GGTGGcctcatTCACA | -312 | 8.67 |
| IMS101 | 3 | Tery_1627 | - | CGTGAgactatTCACT | -349 | 8.59 |
| IMS101 | 4 | Tery_2346 Tery_2345 | - - | TGTGAtgactaTCCCC | -85 | 8.57 |
| IMS101 | 7 | Tery_2502 | - | AGTGCgatcgcTCACT | -302 | 8.45 |
| IMS101 | 8 | Tery_4209 | - | CGTGAgcatttCCACA | -634 | 8.43 |
| IMS101 | 10 | Tery_3484 | - | AGTGCttttttCCACA | -69 | 8.33 |
| IMS101 | 15 | Tery_2476 Tery_2475 | - - | GGTGTataattTCACA | -212 | 8.25 |
| IMS101 | 23 | Tery_0339 | - | AGTGTcaccatTCTCA | -186 | 8.14 |
| IMS101 | 26 | Tery_2255 | - | TGAGAtatttaTCCCA | -466 | 8.12 |
| IMS101 | 28 | Tery_2530 | - | AGTGAtttgaaTCACA | -133 | 8.12 |
| IMS101 | 32 | Tery_0754 Tery_0755 | - - | TGCGCcttaaaCCACT | -488 | 8.10 |
| IMS101 | 33 | Tery_3906 Tery_3907 | - - | TGTGAaaaataCCACA | -570 | 8.09 |
| A-Prime | 1 | CYA_0977 CYA_0976 | - tas | AGAGAtacggaTCACA | -180 | 7.67 |
| A-Prime | 2 | CYA_2457 CYA_2458 CYA_2459 CYA_2460 CYA_2461 | - - - - - | GGTGAtcgggaTCCCG | -113 | 7.48 |
| A-Prime | 8 | CYA_2527 | - | GGTGAtggtggGCACC | -776 | 6.91 |
| A-Prime | 10 | CYA_2632 | - | AGGGAtccagaTCACT | -85 | 6.89 |
| A-Prime | 11 | CYA_2315 | - | AGGGAacgagaTCACA | -179 | 6.86 |
| B-Prime | 1 | CYB_2927 | - | AGAGAtacagaTCACA | -184 | 7.71 |
| B-Prime | 2 | CYB_0465 CYB_0464 CYB_0463 | - - - | GGTGAtcggttTCACC | -112 | 7.61 |
| B-Prime | 3 | CYB_1665 CYB_1666 CYB_1667 | acpP fabF - | GGTGAggctgcTCACA | -52 | 7.51 |
| B-Prime | 6 | CYB_1142 | - | CGTGTtcggtaACACA | -654 | 7.29 |
| B-Prime | 8 | CYB_2776 | - | AGTGAtctgaaTCACT | -397 | 7.20 |
| B-Prime | 15 | CYB_2030 | - | AGGGAacgagaTCACA | -179 | 6.91 |
| MIT_9303 | 1 | P9303_10971 P9303_10981 | - - | TGTGAgctcgtTCACA | -595 | 8.09 |
| MIT_9303 | 7 | P9303_24381 P9303_24391 P9303_24401 P9303_24411 | - - - - | GGTGAttttgcTCACA | -713 | 7.71 |
| MIT_9303 | 8 | P9303_11191 | - | TGTGAggttttATACA | -43 | 7.71 |
| MIT_9303 | 12 | P9303_12331 P9303_12341 | - - | TGTGAcaactaGCACG | -471 | 7.54 |
| MIT_9303 | 15 | P9303_20811 | - | TGTGAgcttaaTCACA | -103 | 7.45 |
| MIT_9303 | 17 | P9303_15451 | - | GGTGAtagcaaTCACA | -263 | 7.32 |
| MIT_9303 | 20 | P9303_04481 P9303_04491 | fer - | TGTGAcaatcaACACG | -664 | 7.27 |
| MIT_9303 | 23 | P9303_06191 | - | TGTGAtcctgcTCACT | -359 | 7.22 |
| MIT_9303 | 38 | P9303_02951 P9303_02941 | - - | TGCGAtgtctaACACA | -153 | 7.01 |
| MIT_9303 | 51 | P9303_27701 P9303_27711 | - - | CTTGAaagtgaTCACC | -110 | 6.85 |
| MIT_9303 | 55 | P9303_00661 | - | TGTGAggaaaaGCACA | -93 | 6.82 |
| MIT9313 | 1 | PMT1007 | - | AGTGAtggtctTCACA | -773 | 8.33 |
| MIT9313 | 3 | PMT2221 | - | AGTGTtggggaTCACT | -296 | 7.95 |
| MIT9313 | 4 | PMT1008 | - | TGTGAactcgcTCACA | -565 | 7.92 |
| MIT9313 | 9 | PMT0992 | hli7 | TGTGAggttttATACA | -57 | 7.73 |
| MIT9313 | 11 | PMT0265 PMT0266 PMT0267 | - - - | TGTGAgctcgaTCACA | -78 | 7.66 |
| MIT9313 | 24 | PMT1492 | - | TGTGAtgttggTCACA | -331 | 7.27 |
| MIT9313 | 28 | PMT1940 PMT1941 | - - | TGTGAgcaagaTCACA | -95 | 7.24 |
| MIT9313 | 35 | PMT1656 | - | AATGTtgtcagTCACG | -37 | 7.17 |
| MIT9313 | 49 | PMT0328 | - | TGTGAagcaagTCACA | -78 | 6.93 |
| MIT9313 | 48 | PMT0487 PMT0488 | - - | TGTGAtcaaggTCAAG | -305 | 6.93 |
| CC9605 | 1 | Syncc9605_1714 | - | TGTGAtcaagaTCACA | -104 | 7.36 |
| CC9605 | 2 | Syncc9605_0559 Syncc9605_0560 | - - | TGTGAtcaagaTCACA | -200 | 7.34 |
| CC9605 | 3 | Syncc9605_1000 Syncc9605_0999 Syncc9605_0998 Syncc9605_0997 Syncc9605_0996 | - - - - - | AGTGAaatagcGCACA | -78 | 7.27 |
| CC9605 | 4 | Syncc9605_2504 Syncc9605_2505 Syncc9605_2506 | - - - | CGTGAcccacaTCACC | -50 | 7.17 |
| CC9605 | 5 | Syncc9605_1249 | - | AGTGAaaaaacTCACT | -38 | 6.90 |
| CC9605 | 7 | Syncc9605_2489 | argJ | TGTGAgcttgaTCACA | -633 | 6.77 |
| CC9605 | 10 | Syncc9605_2507 | - | TGTGAtcaacaTCACA | -711 | 6.73 |
| CC9605 | 28 | Syncc9605_0529 | - | CGTGAtgaacaGCGCA | -470 | 6.57 |
| CC9605 | 34 | Syncc9605_1709 | - | AGTGAaagactGCACT | -110 | 6.54 |
| CC9311 | 1 | sync_1260 | - | TGTGAcgaagaTCACA | -98 | 7.32 |
| CC9311 | 3 | sync_0351 sync_0352 sync_0353 | - coaBC psbO | TGTGAttttgcTCACT | -291 | 7.23 |
| CC9311 | 6 | sync_1258 | - | TGTGAagttatTCACT | -606 | 7.04 |
| CC9311 | 14 | sync_1291 | - | TGTGTatgccaTCACG | -294 | 6.86 |

1. The rank of palindromic CRP boxes recovered by our scanning algorithm.

2. Positions of the putative CRP binding sites relative to the first codon in the TU.

3. The score of CRP boxes recovered by our scanning algorithm.

**Table S2**. Most conserved putative CRP regulated genes/TUs in 12 cyanobacterial genomes.

| # | Genome | Transcription unit | | Name | Annotation |
| --- | --- | --- | --- | --- | --- |
| 1 | MBIC11017 | AM1_4520 AM1_4521 AM1_4522 | | - - - | hypothetical protein, FHA domain protein, hypothetical protein |
|  | ATCC29413 | Ava_0819 Ava_0820 Ava_0821 | | - - - | hypothetical protein, hypothetical protein, hypothetical protein |
|  | A-prime | CYA_2690 | | - | FHA domain protein |
|  | B-prime | CYB_1386 | | - | FHA domain protein |
|  | PCC7120 | all4085 all4084 | | - - | endonuclease V, hypothetical protein |
| 2 | 7120 | alr1196 alr1197 alr1198 alr1199 | | - - - - | hypothetical protein, hypothetical protein, hypothetical protein, hypothetical protein |
|  | MIT9303 | P9303_04171 | | - | small mechanosensitive ion channel (MscS family protein) |
|  | PCC6803 | slr0509 slr0510 | | - - | alkaline phosphatase like protein, hypothetical protein |
|  | BP-1 | tlr1736 | | - | hypothetical protein |
| 3 | ATCC29413 | Ava_4709 Ava_4710 | | - - | microcompartments protein, microcompartments protein |
|  | PCC7120 | alr0317 alr0318 | | ccmK - | carbon dioxide concentrating mechanism protein, carbon dioxide concentrating mechanism protein |
|  | PCC6803 | slr1838 slr1839 slr1840 | | ccmK ccmK - | carbon dioxide concentrating mechanism protein CcmK, carbon dioxide concentrating mechanism protein CcmK, hypothetical protein |
|  | IMS101 | Tery_4328 Tery_4329 | | - - | microcompartments protein, microcompartments protein |
| 4 | ATCC29413 | Ava_1074 Ava_1075 Ava_1076 Ava_1077 | | - - - - | hypothetical protein, Small GTP-binding protein domain, Ferrous iron transport B-like, FeoA |
|  | PCC7120 | alr2118 alr2119 asr2120 | | - - - | iron(II) transporter, iron(II) transporter, hypothetical protein |
|  | PCC6803 | ssr2333 slr1392 | | - feoB | hypothetical protein, ferrous iron transport protein B |
|  | IMS101 | Tery_2879 Tery_2878 | | - - | FeoA, ferrous iron transport protein B |
| 5 | ATCC29413 | Ava_0613 | | - | Periplasmic Sensor Signal Transduction Histidine Kinase |
|  | PCC7120 | alr1192 | | - | two-component sensor histidine kinase |
|  | MIT9313 | PMT0265 PMT0266 PMT0267 | | - - - | two component sensor histidine kinase, putative ABC transporter, hypothetical protein |
|  | MIT9303 | P9303_20811 | | - | Signal transduction histidine kinase |
| 6 | MIT9313 | PMT2221 | | - | possible serine protease inhibitor |
|  | MIT9303 | P9303_29661 | | - | hypothetical protein |
|  | CC9311 | sync_2863 | | - | Ecotin precursor |
| 7 | MBIC11017 | AM1_4906 | | - | hypothetical protein |
|  | PCC6803 | sll1247 | | - | hypothetical protein |
|  | BP-1 | tll0142 tll0141 | | - - | hypothetical protein, hypothetical protein |
| 8 | MIT9313 | PMT1493 | | BioA | putative diaminopelargonic acid synthase |
|  | A-prime | CYA_0687 CYA_0688 CYA_0689 | | ISSoc1 - - | ISSoc1 (transposase), succinate dehydrogenase/fumarate reductase (flavoprotein subunit), beta-alanine--pyruvate transaminase |
|  | B-prime | CYB_2126 CYB_2127 CYB_2128 | | ISSoc1 - - | ISSoc1 (transposase), succinate dehydrogenase/fumarate reductase (flavoprotein subunit), beta-alanine--pyruvate transaminase |
| 9 | IMS101 | Tery_0595 | | - | hypothetical protein |
|  | MIT9313 | PMT2025 PMT2024 PMT2023 PMT2022 PMT2021 PMT2020 PMT2019 PMT2018 PMT2017 PMT2016 PMT2015 | | - - - - gltA ndhA ndhI ndhG ndhE ppnK - | possible Fungal Zn(2)-Cys(6) binuclear cluster, hypothetical protein, hypothetical protein, possible Leishmanolysin, citrate synthase, NADH dehydrogenase subunit H, NADH dehydrogenase subunit I, NADH dehydrogenase subunit J, NADH dehydrogenase kappa subunit |
|  | MIT9303 | P9303_26951 P9303_26941 P9303_26931 P9303_26921 P9303_26911 P9303_26901 | | - - sixA gltA ndhA - | possible Fungal Zn(2)-Cys(6) binuclear cluster, hypothetical protein, Phosphohistidine phosphatase SixA, Citrate synthase, putative respiratory-chain NADH dehydrogenase subunit, hypothetical protein |
| 10 | MIT9313 | PMT2080 | | - | Proline-rich region |
|  | MIT9303 | P9303_27691 | | - | Proline-rich region |
|  | CC9605 | Syncc9605_2479 | | - | hypothetical protein |
| 11 | PCC6803 | sll0564 sll0565 | | - - | hypothetical protein, hypothetical protein |
|  | PCC7120 | alr3037 alr3038 | | - - | two-component sensor histidine kinase, hypothetical protein |
|  | ATCC29413 | Ava_0873 Ava_0872 | | - - | Histidine Kinase, hypothetical protein |
| 12 | MIT9303 | P9303_15781 P9303_15791 P9303_15801 P9303_15811 P9303_15821 P9303_15831 | | - - - - gatA dnaE | STAS domain:Anti-sigma factor antagonist, hypothetical protein, rRNA methylase, hypothetical protein, Glutamyl-tRNA(Gln) amidotransferase A subunit, DNA polymerase III alpha subunit |
|  | MIT9313 | PMT0652 PMT0651 PMT0650 PMT0649 PMT0648 PMT0647 | | - - - - gatA dnaE | STAS domain:Anti-sigma factor antagonist, hypothetical protein, tRNA/rRNA methyltransferase (SpoU):RNA methyltransferase TrmH..., hypothetical protein, Glutamyl-tRNA(Gln) amidotransferase A subunit, DNA polymerase III subunit alpha |
|  | A-prime | CYB_2828 CYB_2829 CYB_2830 CYB_2831 | | | hypothetical protein, RNA methyltransferase(TrmH family & group 3), hypothetical protein, ferredoxin(2Fe-2S) |
| 13 | MBIC11017 | AM1_0938 AM1_0939 AM1_0940 AM1_0941 AM1_0942 | - petL gatB - - | | hypothetical protein, cytochrome b6f complex subunit PetL, aspartyl/glutamyl-tRNA amidotransferase B subunit, hypothetical protein, Cytosine/adenosine deaminase |
|  | B-prime | CYB_2019 CYB_2018 | - ureE | | hypothetical protein, urease accessory protein |
|  | BP-1 | tlr0651 tlr0652 tlr0653 tlr0654 tlr0655 tlr0656 | - - - - - - | | hypothetical protein, ribonuclease II, hypothetical protein, hypothetical protein, ABC transporter ATP-binding protein, pseudouridylate synthase |
| 14 | IMS101 | Tery_0486 | - | | hypothetical protein |
|  | PCC7120 | asl4328 all4327 | - - | | hypothetical protein, aspartate aminotransferase |
|  | ATCC29413 | Ava_1278 | - | | hypothetical protein |
| 15 | MIT9313 | PMT1524 | - | | small mechanosensitive ion channel (MscS family) |
|  | MIT9303 | P9303_04171 | - | | small mechanosensitive ion channel (MscS family protein) |
|  | PCC6803 | slr0509 slr0510 | - - | | alkaline phosphatase like protein, hypothetical protein |
| 16 | A-prime | CYA_0127 | - | | hypothetical protein |
|  | B-prime | CYB_2776 | - | | hypothetical protein |
| 17 | PCC7120 | all0853 all0852 | - - | | two-component sensor histidine kinase, hypothetical protein |
|  | ATCC29413 | Ava_4457 Ava_4456 | - - | | GAF Sensor Signal Transduction Histidine Kinase, Streptomyces cyclase/dehydrase |
| 18 | MIT9313 | PMT1006 PMT1005 | - - | | possible Cystine-knot domain, possible Gamma-thionins family |
|  | MIT9303 | P9303_10971 P9303_10981 | - - | | hypothetical protein, possible Gamma-thionins family protein |
| 19 | MBIC11017 | AM1_3604 AM1_3603 AM1_3602 | - - ubiA | | hypothetical protein, hypothetical protein, 4-hydroxybenzoate polyprenyl transferase |
|  | BP-1 | tll2194 tll2193 tll2192 | - - glyS | | hypothetical protein, hypothetical protein, 4-hydroxybenzoate polyprenyl transferase |
| 20 | PCC7120 | alr0208 | - | | hypothetical protein |
|  | ATCC29413 | Ava_2699 | - | | hypothetical protein |
| 21 | B-prime | CYB_2927 CYB_2928 | - tas | | hypothetical protein, aldo/keto reductase Tas |
|  | A-prime | CYA_0977 CYA_0976 | - tas | | hypothetical protein, aldo/keto reductase Tas |
| 22 | MBIC11017 | AM1_3321 AM1_3322 AM1_3323 AM1_3324 AM1_3325 AM1_3326 | - - - - - cbiB | | general secretion pathway protein, hypothetical protein, hypothetical protein, hypothetical protein, hypothetical protein, cobalamin biosynthesis protein CbiB |
|  | PCC6803 | slr2015 slr2016 slr2017 slr2018 | - - - - | | hypothetical protein, hypothetical protein, hypothetical protein, hypothetical protein |
| 23 | MIT9313 | PMT2025 PMT2024 PMT2023 PMT2022 PMT2021 PMT2020 PMT2019 PMT2018 PMT2017 PMT2016 PMT2015 | - - - - gltA ndhA ndhI ndhG ndhE ppnK - | | possible Fungal Zn(2)-Cys(6) binuclear cluster, hypothetical protein, hypothetical protein, possible Leishmanolysin, citrate synthase, NADH dehydrogenase subunit H, NADH dehydrogenase subunit I, NADH dehydrogenase subunit J, NADH dehydrogenase kappa subunit |
|  | MIT9303 | P9303_26951 P9303_26941 P9303_26931 P9303_26921 P9303_26911 P9303_26901 | - - sixA gltA ndhA - | | possible Fungal Zn(2)-Cys(6) binuclear cluster, hypothetical protein, Phosphohistidine phosphatase SixA, Citrate synthase, putative respiratory-chain NADH dehydrogenase subunit, hypothetical protein |

**Table S3.** Predicted CRP binding sites in *Acaryochloris marina* MBIC11017 genome at *P* < 0.01

| Rank | Transcription Unit | Names | CRP TFBS | Downstream region of the CRP TFBS | CRP site position | Score |
| --- | --- | --- | --- | --- | --- | --- |
| 1 | AM1_3321 AM1_3322 AM1_3323 AM1_3324 AM1_3325 AM1_3326 | - - - - - cbiB | TGTGAtctagaTCACC | CAAGTTGGTGATTTCGATTCT**TAGACT**AGAC | -652 | 8.44 |
| 2 | AM1_3604 AM1_3603 AM1_3602 | - - ubiA | CGTGAtccttaTCACT | ATCGATACTGTTGGTAAAACACAG**TCTATT**T | -35 | 8.25 |
| 3 | AM1_2586 | - | GGTGAccagacTCACT | TTAGATATTTTCATTTGTTTT**TATGAT**AAGA | -133 | 8.16 |
| 4 | AM1_4906 | - | AGTGAcctggaTCACT | CTTTAAAGTGCGTTTGATCACACC**TAAATT**A | -193 | 8.16 |
| 5 | AM1_1165 AM1_1166 AM1_1167 | - putP - | CGTGAtccccaTCACT | GAATCGCCTTTATAGGGAAAAC**TAGGGT**GGT | -182 | 8.00 |
| 6 | AM1_3403 AM1_3402 AM1_3401 AM1_3400 | - - - - | CGTGTcattcaTCACC | GAAACAGTTTGGATTTTAA**TAAAGT**CCGGAT | -121 | 7.97 |
| 7 | AM1_2924 AM1_2925 AM1_2926 AM1_2927 | - - - - | AGTGAtttaccTCACA | ACTACCCGTCTTCTAATCTTG**TAATCT**GCAA | -156 | 7.90 |
| 8 | AM1_1025 AM1_1026 AM1_1027 AM1_1028 AM1_1029 | - - - - - | TGTGAtcttccACACA | GCAAGCGGATTTTGTGGT**TCGTTT**AAGCATT | -103 | 7.81 |
| 9 | AM1_2336 | - | AGTGAtgtcaaTCACG | TCTATCGGCAAAGGAAAAGGACGC**AAAGTT**A | -71 | 7.79 |
| 10 | AM1_6020 | - | AGTGAacttgaTCGCT | GTTTAAATTGGCTCGGAAGACTGGA**TATAAT** | -236 | 7.77 |
| 11 | AM1_4101 | - | TGTGActtagaTCACA | TAGTGAATTCTTTTCTCGCGG**TTAAAT**AATT | -173 | 7.76 |
| 12 | AM1_3704 AM1_3706 | - - | TGTGAtttataTCACG | CCAATCACTGGCTGAGAACAT**TACGAT**CAGG | -226 | 7.76 |
| 13 | AM1_1841 | - | CGTGGcacctaTCACA | CTTCCGAAAATGGATAATTTCTG**TAGTAT**TC | -472 | 7.75 |
| 14 | AM1_5560 | - | TGTGAgattcaTCACT | GCAGCTTTTTGGCACAGCGCTT**TAAAGT**GAG | -118 | 7.74 |
| 15 | AM1_1024 AM1_1023 | - - | TGTGTggaagaTCACA | TTACAAATTGCGGATGTG**TTTAAT**GATACAG | -50 | 7.71 |
| 16 | AM1_2824 | - | GGTGAttttgaTCACA | CACATGATTGATTTAAAG**CACGGT**TTAGACA | -98 | 7.68 |
| 17 | AM1_6071 AM1_6070 AM1_6069 | - - - | AGTGActgtagTCACG | GATTGGCCGCCAAACGGTGC**TGAGCT**TAGTC | -712 | 7.68 |
| 18 | AM1_6406 AM1_6405 AM1_6404 AM1_6403 AM1_6402 AM1_6401 AM1_6400 AM1_6399 | - - - - - - - - | AGTGAtcttcaTCCCA | ATAGGTACGACTTTTGGCC**AATAGT**CCCTCT | -75 | 7.67 |
| 19 | AM1_6038 AM1_6039 | - - | TGGGAtcgtccTCACA | CTGCTTGAGTACCTGGATCACAAAT**TATTCT** | -78 | 7.61 |
| 20 | AM1_5181 AM1_5182 | - - | TGTGAttgttgTCACT | GAGAAATCAAGTTGCATCACCCCA**TGGCCT**A | -103 | 7.60 |
| 21 | AM1_2193 AM1_2194 AM1_2195 AM1_2196 AM1_2197 AM1_2198 AM1_2199 AM1_2200 AM1_2202 | - kdsB kdsA kdsC - - - - - | AGTGAtctaatTCACA | GAACTAGACGAGATTTGAGAT**CATCAT**GGAG | -179 | 7.60 |
| 22 | AM1_4520 AM1_4521 AM1_4522 | - - - | GGTGAtgattgTCACG | GCGGCACGGGTATGCTGAGGG**GAAGGT**AAGG | -148 | 7.59 |
| 23 | AM1_4117 AM1_4118 AM1_4119 | - - - | TGTGAgagtgcTCACT | GTGTTGAGCAGTTATGGGAAATCAC**TGTTCT** | -92 | 7.58 |
| 24 | AM1_1753 AM1_1752 AM1_1751 AM1_1750 | ksgA ispE - - | AGTGAtccgaaTCACA | AGAATGTATCGTTAAATA**CATTTT**CCCTAAA | -154 | 7.57 |
| 25 | AM1_3703 | - | CGTGAtataaaTCACA | GCTCTCTAAAGCGGGCCAGCAC**AAGCTT**GTA | -308 | 7.54 |
| 26 | AM1_2923 AM1_2922 | - - | TGTGAggtaaaTCACT | GGCGAGGGTACGGAAAAA**TGTATT**CGATCCG | -166 | 7.53 |
| 27 | AM1_3599 | thiG | TGTGAgatactTCACT | AGCCTAGCGGCAGTTTCCC**TTCATT**GCTGAA | -57 | 7.48 |
| 28 | AM1_5746 AM1_5745 AM1_5744 AM1_5743 AM1_5742 | - - - - - | AGTGAgtttgaTCACC | TTTCAGCCCTGGGTTTTCTA**AAAGTT**GCGGA | -149 | 7.48 |
| 29 | AM1_1272 AM1_1273 AM1_1274 AM1_1275 | - - serA prmA | AGTGAgaatatGCACT | TTGTTGCATATTACATCGTC**TGCGTT**GCCTA | -45 | 7.47 |
| 30 | AM1_3950 AM1_3951 | - - | TGTGActagtaTCACA | AACTAGTAAACTCAGTCGTTG**TACAGT**CAAG | -272 | 7.45 |
| 31 | AM1_4103 | - | TGTGAtctaagTCACA | TAATTGCTGAATTTGCAA**TAGAGT**ATTTAAA | -263 | 7.44 |
| 32 | AM1_5474 | - | GGTGAtgggcaTCACA | AGGGCTAGGCCAGTAACCC**TAGCTT**TTTGCC | -481 | 7.44 |
| 33 | AM1_5747 | - | GGTGAtcaaacTCACT | GATTAGAGTATGAACCATAATC**TTTAAT**GAT | -210 | 7.44 |
| 34 | AM1_2831 AM1_2832 | - - | CGTGAtccagaTCACA | AATGCAAGCCGCTATAGTC**ACTGCT**CGCAGG | -135 | 7.44 |
| 35 | AM1_4513 AM1_4514 | - - | CGTGAggacaaCCACA | CGAATTGTTGGGTAAGTATGTT**CAGCAT**TCC | -726 | 7.42 |
| 36 | AM1_0773 AM1_0772 | modA - | CGTGTtcgtatACACA | AATTTGCCTCATCTGAAAGCCT**TACGCT**TCG | -138 | 7.41 |
| 37 | AM1_4962 | - | GGTGAtgggtgTCCCA | TGTGTAAGTTTTAAGGTT**TGCTTT**TTGACTC | -489 | 7.41 |
| 38 | AM1_2210 AM1_2209 | - - | TGTGAtttgagTCACT | TGAATTTCTTTTATTGTTTATT**TATACT**CTT | -204 | 7.40 |
| 39 | AM1_5354 | - | TGTGAtataaaTCACG | TTCAATATCTCTAGAAAT**TATTGT**AATAGAA | -95 | 7.40 |
| 40 | AM1_0112 AM1_0113 AM1_0114 AM1_0115 | moaC - - - | CGTGCtctagtCCACT | GAGCTACAAGCCCCTAAGGGAT**TATCAT**CTT | -100 | 7.39 |
| 41 | AM1_4957 | - | AGTGAtttccaTCACA | CCTATTTTGCCGTGAAACGGGA**TAGGCT**CTT | -133 | 7.38 |
| 42 | AM1_2114 AM1_2113 AM1_2112 AM1_2110 | glgP - - - | AGTGCtgcacaGCACA | AACATGATTAACATTGAATGATAA**TAATCT**G | -95 | 7.38 |
| 43 | AM1_2588 AM1_2589 | - - | AGTGAgtctggTCACC | ATCACTAATAGTTCTAAA**TAATTT**ATTAAAA | -475 | 7.37 |
| 44 | AM1_3535 | - | CGTGGtagagtTCACA | ATTCTGTATAAGGCGATACAT**TTTTTT**CTGG | -141 | 7.37 |
| 45 | AM1_4956 AM1_4955 AM1_4954 | - - - | TGTGAtggaaaTCACT | TCAAGGGGGTGTAACGCTCACC**AATCCT**TGG | -277 | 7.36 |
| 46 | AM1_0346 | - | AGTGAtgggaaTCCCT | CGGGCGTGTGCTAGAAGAGTCCAGG**TCTAGT** | -143 | 7.36 |
| 47 | AM1_5530 | - | CGTGAcaactgTCACG | TTCAGTCCCATTTCCTCCGCC**TAAATT**ATAA | -124 | 7.36 |
| 48 | AM1_5686 AM1_5687 | - - | AGAGAtactatTCACA | CCAATCGGTTCTGTACCT**TAGGAT**TGGTGTG | -73 | 7.36 |
| 49 | AM1_6037 | - | TGTGAggacgaTCCCA | GGTGGTCTTGATTCATGGA**TACAGG**AGGAGC | -223 | 7.34 |
| 50 | AM1_3044 | - | TGTGAaatctaCCTCG | GCGGGCAGTCCAACACTAGCCAG**TAGAAT**CA | -141 | 7.34 |
| 51 | AM1_3820 | - | CGTGActgtaaTCACT | GCAGCGCATTATGCTGAAAAACT**TTCTAT**GA | -381 | 7.31 |
| 52 | AM1_2986 | - | CGTGAgaagccACACA | TTGGACGCCTATTATTAGAGGAT**TCATTT**AG | -255 | 7.31 |
| 53 | AM1_6031 AM1_6030 AM1_6029 | - cydA cydB | TGGGAtttaaaTCACA | TCACAAACAATCAATATAAGT**TACACT**GGGG | -112 | 7.31 |
| 54 | AM1_5907 AM1_5908 AM1_5909 AM1_5910 AM1_5911 AM1_5912 | - - - - - - | AGTGAagctggTCCCT | TATACCTTGTCCGAAGCAGG**GGGATT**GGGGG | -67 | 7.28 |
| 55 | AM1_5169 | - | CGTGGggtaccTCTCT | TCAGACTGTATTCCAGGACAC**TACTCT**AGGG | -122 | 7.28 |
| 56 | AM1_2115 AM1_2116 AM1_2117 AM1_2118 | msrB - - ilvN | TGTGCtgtgcaGCACT | AGAAGCCACCCCTTAACTTCA**TAATTT**AGGA | -399 | 7.27 |
| 57 | AM1_5553 | - | AGTGAgtgtacCCACT | TTTTTGCCTTGGCAAAGATGGCAGG**TTTCTT** | -72 | 7.27 |
| 58 | AM1_1158 AM1_1159 | - - | TGTGAatttatTCGCA | AGGGCGAGGGCTAATCCACACTGT**TCAAGT**T | -185 | 7.26 |
| 59 | AM1_2489 AM1_2488 AM1_2487 AM1_2486 | - - - - | AGTGTtaaaggGCACC | CTATTTCTCTAGCCAGTCAAT**TACTGT**GAGA | -146 | 7.26 |
| 60 | AM1_2725 | - | AGTGGgggactTCACC | TGTTTTTTTCACAGATATTCACAA**TCAGAT**T | -257 | 7.26 |
| 61 | AM1_5723 AM1_5722 | - - | AGTGGgaatgcTCACA | GAAAACATATGGAGTCATACGG**TATTCT**TCT | -146 | 7.26 |
| 62 | AM1_2844 AM1_2843 AM1_2842 AM1_2841 AM1_2840 AM1_2839 | - - - - anmK - | TGTGTgctaggACACG | ACTAGGACAAACCATTGTTGGCGA**TAGCCT**G | -424 | 7.24 |
| 63 | AM1_2427 | - | TGTGTttaacgTCACA | GTATTTCTTGAGTGCTAG**TACCTT**TTTGAGT | -130 | 7.24 |
| 64 | AM1_4519 | - | CGTGAcaatcaTCACC | GCGATCTCAAGCTTCTACAGGC**TATAGA**TGG | -158 | 7.23 |
| 65 | AM1_2717 | - | GGTGAagctttTCACT | GTCATGTAGTTTATAGAAATAGTTC**CACACT** | -204 | 7.23 |
| 66 | AM1_4966 AM1_4967 | - - | TGTGAtctgaaTTACA | AACTTATGTTAATATCCGCA**TAATTT**TTTGA | -705 | 7.21 |
| 67 | AM1_1395 AM1_1396 AM1_1397 AM1_1398 | pilM pilN pilO - | AGTGGgtatccTCCCA | ACAGAACAAAGCCTCAAACAT**TAGGTT**TAAT | -75 | 7.21 |
| 68 | AM1_3118 | wecB | AGTGTaattgaTCACT | GCGCCTCTCTTTTACGATAAACA**TGAGTT**AT | -99 | 7.21 |
| 69 | AM1_0186 | - | TGAGCagtgccTCACC | GGCATGCTCAGGATTGTTTTG**TATATT**GACT | -94 | 7.21 |
| 70 | AM1_5529 | uvrB | CGTGAcagttgTCACG | GGGGGGTATTGTTCTATGT**CATGCT**GCTGCC | -81 | 7.21 |
| 71 | AM1_0481 AM1_0482 AM1_0483 AM1_0484 | - - - - | AGTGAgaggcaTCTCC | TCCAGACCCGCTCAGCATTGGGCG**AACCTT**G | -106 | 7.19 |
| 72 | AM1_2461 AM1_2460 AM1_2459 | - - - | AGTGCcgctgcTCACT | GATTGTAACCTCTGCGAACAAAG**GATGCT**CA | -104 | 7.19 |
| 73 | AM1_1071 | - | TGTGCtgccgtGCTCT | GCTGCAGTACCAATTCTTCTTTCA**TAAATT**T | -168 | 7.19 |
| 74 | AM1_4900 | - | TGTGGagtaatCCACG | GGGGGACTTATTACGAGTGACTC**TACTCT**AT | -70 | 7.18 |
| 75 | AM1_0938 AM1_0939 AM1_0940 AM1_0941 AM1_0942 | - petL gatB - - | AGTGAagaacaACACC | AAGGAATCGGCCTGCTTTGGAACTG**AATAAT** | -344 | 7.17 |
| 76 | AM1_1559 AM1_1558 | - apcA | AGTGTtgtgctTCACA | AATAGCTCTGTTGAAATTGATGTG**CAGCGT**T | -641 | 7.17 |
| 77 | AM1_3844 AM1_3843 AM1_3842 AM1_3841 AM1_3840 | - - - - - | TGTGAggttaaTCCCT | ACAATGTGAAGATGAGAAT**GAGTAT**TTATGC | -49 | 7.16 |
| 78 | AM1_0227 | - | TGTGActcagaTCACA | GTAGCTTGAAATAATTGTCAT**TGTAAT**GAAC | -225 | 7.16 |
| 79 | AM1_4201 AM1_4202 AM1_4203 AM1_4204 AM1_4205 AM1_4206 AM1_4207 | - - - - - - - | TGAGAaagcaaTCGCA | GATAAATATTCAATTTTTGTG**TAATTT**TTTA | -169 | 7.15 |
| 80 | AM1_5554 | - | AGTGGgtacacTCACT | GAACAGCTAACGCTATTGAATTGAA**AATTTT** | -718 | 7.15 |
| 81 | AM1_4673 AM1_4671 | - - | GGTGAcatgggCCACT | AACTGATGGCCATAGAGACT**CATGAT**CAGAG | -187 | 7.14 |
| 82 | AM1_2295 AM1_2294 | acsF - | GGTGActtagcGCCCT | GCATCAACCGGGCATTCAGG**TAATAT**TGCTT | -167 | 7.14 |
| 83 | AM1_1453 | rpsA | GGTGAatagcgTCCCA | ACAATATTTCTCACATTTCT**TGCGGT**TAAAA | -128 | 7.13 |
| 84 | AM1_3949 | - | TGTGAtactagTCACA | GTCCCTTAAAATCAGTCAAACGA**TCAGTT**CG | -565 | 7.12 |
| 85 | AM1_0226 | - | TGTGAtctgagTCACA | AAACGCTTAGAACACCTGTTTGAAA**TGCTTT** | -620 | 7.12 |
| 86 | AM1_3804 AM1_3805 | - - | CGTGGtcagggACACG | ATGTTTATAGCCCTCAGACTGACAG**TAGAGT** | -113 | 7.12 |
| 87 | AM1_1590 AM1_1591 | - - | CGTGAccttgaTCGCC | AATTCGCCGATCAGCTCGGACCA**GAAGAT**CA | -511 | 7.11 |
| 88 | AM1_1670 | - | AGTGAgaagccTCTCA | TACTCTGACTGAGCAAGAGT**TCTAGT**TAACT | -516 | 7.11 |
| 89 | AM1_3600 | rpoE | AGTGAagtatcTCACA | TTATAAGAAACCAACTTGC**GGTCAT**GGGAAC | -89 | 7.10 |
| 90 | AM1_3534 AM1_3533 | amtB amtB | TGTGAactctaCCACG | TGTTGAATCGATTAAATCAG**TGGGTT**GGGTG | -183 | 7.10 |
| 91 | AM1_4901 | - | CGTGGattactCCACA | CTTAGTGACTACCGTTTTTGAT**TCAGAT**TCC | -248 | 7.10 |
| 92 | AM1_0058 AM1_0059 AM1_0060 | - - - | AGTGAtaaacaACACG | ATCTCTTGTGACACACATCA**TATTTT**TCAAC | -154 | 7.10 |
| 93 | AM1_0611 | - | CGTGAtaattgGCACA | ACCCTGACAAGGAGACACTGCTGA**TTCCGT**C | -231 | 7.10 |
| 94 | AM1_3483 AM1_3484 AM1_3485 | - - - | AGGGGtgatccTCACA | TTTTCCCCAAGTTTGGAGAG**TATTCT**CCACA | -110 | 7.10 |
| 95 | AM1_5498 | - | TGCGAtacattTCACC | CTAAGTAATCCTGTAAATCCTAAA**TAAGAT**T | -177 | 7.10 |
| 96 | AM1_2214 | - | GGTGActaggaCCACG | GCCTGTGCCTGGCAACAGTT**TATGAT**AGGTT | -77 | 7.09 |
| 97 | AM1_4512 AM1_4511 AM1_4510 AM1_4508 AM1_4507 | purU - - - - | TGTGGttgtccTCACG | CTTTGTCTTTGCAAGCTCAA**TCGGCT**CAATG | -96 | 7.09 |
| 98 | AM1_1310 | - | TGTGAtattaaTCTCA | ACAGAATTTAGTTTGATGTGAA**TAAATT**GCT | -119 | 7.09 |
| 99 | AM1_6009 | - | TGTGAtctaagCCACT | TCTTTATAACTTTCCGGTACT**TAGTAT**AAAA | -65 | 7.07 |
| 100 | AM1_5882 AM1_5881 AM1_5880 AM1_5879 | - - - - | GGTGAtgtcatCCTCT | TTCTTGGTTCATTGAGGG**TTCGCT**GCAACCT | -93 | 7.07 |
| 101 | AM1_4861 | - | TGTGAttacatTCCCT | CAAAACAGCCTACTAGCC**TCGTGT**ATCCAAG | -197 | 7.06 |
| 102 | AM1_0603 AM1_0604 AM1_0605 | - thiL - | CGTGGggtggcTCACG | ATCGAGCTGGCTTGATTTAAGTTGC**AATTTT** | -40 | 7.05 |
| 103 | AM1_5172 AM1_5173 AM1_5174 | - - - | TGTGGaagactTCTCA | TTCAACTCTCAGGGAACAC**TCAGCT**AAGTCA | -97 | 7.05 |
| 104 | AM1_5353 | - | GGTGAgaatcaTCACA | ATGCAGGACGCTGCTCTTATGAGA**GATAAT**T | -317 | 7.05 |
| 105 | AM1_0042 AM1_0043 AM1_0044 AM1_0045 AM1_0046 AM1_0047 | - nblB - - - - | TGTGCtaactgACACA | TAGAAGCTGTGATCCAAAAAC**AATATT**GATA | -216 | 7.04 |
| 106 | AM1_5292 AM1_5291 AM1_5290 | - - - | GGTGGtttgtcGCACG | ATTTGGGTCAAACAATGG**TGTTGT**TGCTGCG | -181 | 7.04 |
| 107 | AM1_2825 AM1_2826 | - - | TGTGAtcaaaaTCACC | CTTAATCCTGCATCTTTTTATTCC**TCATTT**T | -586 | 7.04 |
| 108 | AM1_1656 | - | TGTGActggcgGCACT | AATACTGATGAGTTATGGCCC**TTGGAT**ATAA | -311 | 7.04 |
| 109 | AM1_0578 | - | GGTGAacaagaTCACC | AGCGCCAGCTGGCTAATCGACTGAT**TTTGTT** | -308 | 7.04 |
| 110 | AM1_2462 AM1_2463 | - - | AGTGAgcagcgGCACT | TTGCAATGAATCCTTTGG**TGACCT**ACTTCTG | -42 | 7.04 |
| 111 | AM1_2046 | chlP | CGTGAtacgatTCGCT | ACGTAGCCGTTAAAAGTTTGGAGGA**TCGCCT** | -32 | 7.03 |
| 112 | AM1_4552 AM1_4551 | - - | CGTGAacaaaaTCACA | ACCGTCAATTGCTATGACCA**AATCTT**CGTAG | -336 | 7.03 |
| 113 | AM1_3317 | - | CGTGAtttgaaTAACA | GCTTTCTCAAAGCCATTCAAG**TACCGT**GAAC | -78 | 7.03 |
| 114 | AM1_3898 AM1_3899 AM1_3900 | hli - - | AGTGAcaatggACTCA | TTACCGCTTTCAGCAGGGTGGCT**TGTCAT**GA | -206 | 7.03 |
| 115 | AM1_3076 | - | TGTGGggatcaTCTCA | AAGTGTATGTGTCAAAATT**TTTAGT**TAAGGA | -374 | 7.03 |
| 116 | AM1_1687 AM1_1688 | - glpK | TGTGAaagtttTCCCA | ATCCGAAGACTCATAGGTGA**AGTGGT**AGGGG | -621 | 7.03 |
| 117 | AM1_5208 | - | AGTGAttaagaTGACA | CCTCATGGCGATGGGGTTTGT**TAGCCT**TTGT | -182 | 7.02 |
| 118 | AM1_1562 | - | AGTGTagtcagCCACC | TGTCGAAAGATAGATAGT**TCTTAT**ATCTAAA | -273 | 7.02 |
| 119 | AM1_4750 AM1_4749 AM1_4748 | - - - | TGTGActcgctGCACC | TCACTTGGACAGGGTGGCTGTG**TATCTG**TGG | -248 | 7.01 |
| 120 | AM1_1825 AM1_1824 | - - | TGTGAaggggtTCCCC | CACATAGGGATGCAAAAAAACC**GACGCT**AAT | -785 | 7.01 |
| 121 | AM1_5371 AM1_5372 | - - | GGTGAaatgttACGCT | GTATACCTAGTTAGCAGGGGG**TAAATT**GTGT | -114 | 6.99 |
| 122 | AM1_2966 | - | CGTGAcctgcaGCACT | GCTCGGGTCAGATGATCAAGA**TAAGGT**CTTC | -87 | 6.99 |
| 123 | AM1_3262 | - | AGTGAagtgaaTCACC | TAAATTGAGGAAAAAAATATGGT**TTTAGT**TC | -218 | 6.99 |
| 124 | AM1_2722 AM1_2723 AM1_2724 | - - - | AGTGAgggagaACACG | CGCAGGATCGGATCGCGAGGC**TAGACT**AACG | -60 | 6.99 |
| 125 | AM1_5282 AM1_5283 | - - | AGTGCcattgaTCCCT | TGGAATATCTTTCTCCTTGACAACA**CACTTT** | -421 | 6.99 |
| 126 | AM1_1621 AM1_1622 AM1_1623 | - - - | GGTGCataaagTCTCC | TGCGAAGCCAAAGAGGAGTGCG**TTATCT**ACG | -276 | 6.99 |
| 127 | AM1_3640 AM1_3639 AM1_3638 | - - - | CGTGAgcagagCCCCC | ATCAATCGATCGAGGGATTGA**TATTCT**TCAA | -154 | 6.98 |
| 128 | AM1_5431 | - | GGAGAacctcaGCACG | ATCAACTTTTAGATCTATTAGT**TAGTTT**TCA | -240 | 6.98 |
| 129 | AM1_5500 | - | GGTGAaatgtaTCGCA | GGATACAAAATGGCTATG**AAAAAT**CAGCATC | -148 | 6.98 |
| 130 | AM1_0720 AM1_0721 AM1_0722 AM1_0723 AM1_0724 | - aspH aspH aspH - | AGAGAtgctgaTCGCC | AATTCTCCAATCATTCGTTT**TAATTT**GGGAA | -656 | 6.98 |
| 131 | AM1_2808 | - | CGTGAattcatTCCCT | AATCGATTTGCAAGTTAG**TATTTT**GATGTAT | -64 | 6.98 |
| 132 | AM1_3474 | - | TGTGAtggttaTCACT | TCTCCAGACTTGAACTATCACAGC**TGCTAT**A | -184 | 6.98 |
| 133 | AM1_2261 AM1_2263 | - - | TGTGCaacagtACCCA | ATCGAGGACTAGTCGCTA**TAGGCT**ACGGCTC | -180 | 6.97 |
| 134 | AM1_5107 AM1_5108 | - - | AGTGAaatcttTCTCT | AGAATTATCTCTTGATAGA**TTTTTT**GGGGAG | -198 | 6.97 |
| 135 | AM1_3979 AM1_3980 | gloA - | TGTGAcgaccaCCTCT | TGACTTTATTAACTCTTAAACAAA**TAGTCT**T | -33 | 6.97 |
| 136 | AM1_1560 AM1_1561 | ccb2 - | TGTGAagcacaACACT | CTCTCCCCAACGGATTACTATAAA**GATAGT**C | -55 | 6.97 |
| 137 | AM1_2790 | - | AGTGAcacatcTCACA | GAAATCACCTGAAGCTAATCAC**AAAGAT**GCC | -148 | 6.97 |
| 138 | AM1_2792 | - | TGTGAgatgtgTCACT | GAGTAACCCCAATGGAACTGGCA**TTGTCT**AG | -60 | 6.96 |
| 139 | AM1_5350 AM1_5349 | rplI dnaB | TGTGAtgattcTCACC | TCAACGCATGAGATTGAAA**CGTTCT**AGAGAA | -412 | 6.96 |
| 140 | AM1_4782 AM1_4781 AM1_4780 AM1_4779 AM1_4778 AM1_4777 AM1_4776 | - - - - - - - | TGGGGctgcacTCACC | GTTTATGCGGAAAGCGTCTGGCTA**TGCGGT**G | -132 | 6.96 |
| 141 | AM1_4013 AM1_4014 AM1_4015 AM1_4016 AM1_4017 AM1_4018 | - - - - - - | GGTGAcatgatGCTCA | GTATATGATTTCACTTAGC**TAATAG**AAGGGG | -79 | 6.96 |
| 142 | AM1_2437 AM1_2436 | - ftsH | CGGGAtagctaGCACC | GATCCCAATCCCAGCGATTCGTGA**TAATCT**T | -119 | 6.95 |
| 143 | AM1_3784 AM1_3783 | - - | CGTGAtaagtgTCACT | CGCCTTTCAGGCACATATCAC**TGAAAT**ACTA | -157 | 6.95 |
| 144 | AM1_4185 | - | AGTGCacagaaACACA | ACGAAATGCGCATTGATGATG**TCGGGT**CTTC | -180 | 6.95 |
| 145 | AM1_5140 | - | AGTGCccaccgCCACG | CTACTCTGCCGTATGATTACCAC**TGAATT**CT | -535 | 6.95 |
| 146 | AM1_1075 AM1_1074 AM1_1073 | - - - | GGTGGcaacacTCCCA | AGCGCGGGACTCCCGAGTTTGCC**AAATGT**GC | -618 | 6.94 |
| 147 | AM1_1005 | - | CGTGAtcgcgtTCTCG | TCTGTTTTCTCCTTCAGCAA**TGCAGT**CTCCA | -191 | 6.93 |
| 148 | AM1_0041 AM1_0040 | - - | TGTGTcagttaGCACA | AACCACTGGGCCTACTCCAGAAC**GGATGT**AA | -143 | 6.93 |
| 149 | AM1_0526 | psbO | GGTGCacttgcACCCA | GAGCATAGCACTTACATCCC**TAAAGT**GCAGA | -175 | 6.93 |
| 150 | AM1_2100 AM1_2099 AM1_2097 | - - - | AGCGCctcgtaTCACA | CTGGTGGCGTTCCGGAGGAA**TACCGT**GGCAA | -466 | 6.93 |
| 151 | AM1_2211 | - | AGTGActcaaaTCACA | AAGTAACAATTTTCATCATAG**AAGCCT**TGCA | -741 | 6.92 |
| 152 | AM1_4051 AM1_4050 | - - | TGGGTtgcagcTCACT | ATATGAATCTGAATCTCCAT**TATCTT**GCAAG | -574 | 6.92 |
| 153 | AM1_2791 | - | AGTGAaaatagTCTCA | TACTCTATGGAGAAATTTCCCTAC**AAGCAT**A | -144 | 6.91 |
| 154 | AM1_3031 AM1_3030 AM1_3029 | - - - | TGTGTcgcataACACC | CTCACTTCCACCCCAATTGCGATCA**TTCACT** | -277 | 6.91 |
| 155 | AM1_0632 AM1_0631 AM1_0630 AM1_0629 | - - - - | GGTGAtatatcTATCA | TCAACTAACTCTTTATAAATGACAA**TATGAT** | -70 | 6.91 |
| 156 | AM1_1058 | - | TGTGCtgacacTCCCC | TGACATTTTATCTGGCTAT**TGCTAT**CCCCAG | -181 | 6.91 |
| 157 | AM1_5588 AM1_5587 AM1_5586 | - - - | GGTGCaatagaACTCA | AGGTCATGGGCAGTATAGGGA**TGAAAT**ATGA | -202 | 6.90 |
| 158 | AM1_4675 | - | CGTGGctccaaTCACA | CTTTCTAAATTAGTCGCAA**TAAAAG**CCGCAA | -660 | 6.90 |
| 159 | AM1_3764 AM1_3765 AM1_3767 | - - - | GGCGAtggcccGCACT | GTAACTGTTGATGCCAGCAGCT**TAGGGT**GCC | -79 | 6.89 |
| 160 | AM1_0018 AM1_0017 AM1_0016 AM1_0015 AM1_0014 | - - - - - | TGTTTgcgggaTCACC | CTGATCTTCTTTCTATGCTGAC**TATTTT**GAA | -130 | 6.89 |
| 161 | AM1_5062 | - | TGGGAgaggctTCACC | ATTCTGATAGGTTGATTACTTGCCC**TAAGCT** | -291 | 6.89 |
| 162 | AM1_5490 | glnB | AGTGGggagttTCACA | CTGATATCCCAATGTGTGAAGGTGC**TTTTCT** | -160 | 6.88 |
| 163 | AM1_0166 | - | AGTGGagaatgGCACC | TACACGCCTCTGTCTCGCCC**TATCTT**TATTT | -711 | 6.88 |
| 164 | AM1_2767 AM1_2768 AM1_2769 | - - - | CGTGTaactttCCCCC | GCTAAGCCCAAAGAAAACAC**TCTTTT**GGGTT | -78 | 6.88 |
| 165 | AM1_4910 AM1_4909 AM1_4908 AM1_4907 | - sds lepB chlI | GGTGCttaaccCCACC | ACTAAGGTCTTAAGGGCGATGGC**GATTTT**GA | -286 | 6.88 |
| 166 | AM1_2551 AM1_2550 AM1_2549 | - - - | AGTGAtggataTCACT | GAGAGAATGCTGACCAAT**CACATT**GGCGGTT | -148 | 6.87 |
| 167 | AM1_5223 | dut | AGTGTtgttttACTCC | TCTGTAAGCATTGCAAAAT**TTGGAT**GAATAA | -68 | 6.87 |
| 168 | AM1_1927 | - | CGTGCtctagtCCACT | GAGCTACAAGCCCTAAAGCGTATT**TATGTT**A | -146 | 6.87 |
| 169 | AM1_5142 | - | CGTGGcggtggGCACT | TGGCTTATGCCAAGTCACCC**TCCTGT**TCAAA | -89 | 6.86 |
| 170 | AM1_2335 AM1_2334 | - - | CGTGAttgacaTCACT | CAAGTTTCAGATCCGATTCGCGAA**GATGGT**G | -74 | 6.86 |
| 171 | AM1_3819 AM1_3818 AM1_3817 | - - - | AGTGAttacagTCACG | TGAGCCTTGGATCAACATCACT**GAATCT**GAA | -104 | 6.85 |
| 172 | AM1_2710 AM1_2711 | - - | GGCGAtttgaaTCTCT | CTAAAAATCGAGAACGCTTAGTCGA**TGATAT** | -144 | 6.85 |
| 173 | AM1_1392 | - | AGTGAtacggaTCAAT | CATCGTATTTCCCAAACAGCT**TAAACT**TCAA | -200 | 6.85 |
| 174 | AM1_0990 AM1_0991 | - - | AGGGAtaaatgCCCCA | AAGGTCAGCAACCTGAATGGGGT**TAGGAT**TC | -201 | 6.85 |

**Table S4**. Predicted CRP binding sites in *Anabaena variabilis* ATCC29413 genome at *P* < 0.01

| Rank | Transcription Unit | Names | CRP TFBS | Downstream region of the CRP TFBS | CRP site position | Score |
| --- | --- | --- | --- | --- | --- | --- |
| 1 | Ava_4457 Ava_4456 | - - | TGTGAtttgagTCACA | ACAGTACTAATGCGGTTAATCACAA**TGAAGT** | -362 | 9.25 |
| 2 | Ava_2699 | - | GGTGTtattttTCACC | TTAAATTAAGGTTTACTTAAACT**TAGATT**TA | -194 | 9.24 |
| 3 | Ava_0186 | - | GGTGAtacaagTCACA | AGCTGATACTATCAAAATC**TCAAGT**ATCAAT | -309 | 9.24 |
| 4 | Ava_4877 | - | TGTGAtacagaTCACA | GACGATACTGACTAGGATCACT**AATAAT**TGC | -109 | 9.23 |
| 5 | Ava_2743 | - | GGTGGatgcgaTCGCC | CCAACAGCTAACTACCCTATGTG**TAAGCT**TT | -265 | 8.89 |
| 6 | Ava_4982 | - | AGTGTttttgtTCTCC | ACCCCCTGCATAGCACTTTCAAGC**TAGACT**A | -55 | 8.77 |
| 7 | Ava_1278 | - | TGTGAtctaaaTCACT | TAAATAGCCATGACTGGATCACA**TTTTAT**AC | -82 | 8.77 |
| 8 | Ava_1249 | - | TGTGCtggtatTCACA | AAAGAAGTTTCATGAATACTGTC**GAACAT**AC | -100 | 8.74 |
| 9 | Ava_3123 | - | TGTGTgcgccaGCGCA | TTAATCCAACTATTAGCCCTCC**TAAAAT**GGG | -209 | 8.70 |
| 10 | Ava_2092 | - | GGTGTaaatatACACA | AAAAGTCACTGGCATTAGAAA**TTTACT**GCCT | -658 | 8.68 |
| 11 | Ava_1279 Ava_1280 | - - | AGTGAtttagaTCACA | ATTAAACTCAGTAGATATTTCC**TGATTT**AAC | -377 | 8.66 |
| 12 | Ava_3702 Ava_3703 | - - | CGTGAcagaaaTCTCA | ACATAGCAAAATGTAAAGAA**CAACTT**AAGAA | -178 | 8.63 |
| 13 | Ava_3050 | - | CGAGAgtgtaaTCACC | TTTTCATTGGAAATTTCT**TATTCT**AGGAAAA | -552 | 8.61 |
| 14 | Ava_0873 Ava_0872 | - - | GGTGAattttcTCTCT | GATTGCGATAATGTCCGC**TCTCCT**CGTTAAA | -81 | 8.59 |
| 15 | Ava_0612 | - | CGTGTcagttgCCACT | GGCTAGGGGTTTTATATCTGGAA**TACCAT**CC | -467 | 8.54 |
| 16 | Ava_1862 | - | TGTGGaggcgaTCGCT | CACTCTTTTTACTCAAAGTTTCT**TGTTTT**GA | -79 | 8.54 |
| 17 | Ava_4895 | - | AGGGTatgataTCACG | GGATAAATTTTAATTTTCAATAG**TAAAAT**AA | -206 | 8.52 |
| 18 | Ava_3301 | - | GGTGAgatgctCCACA | TTAGCAGAATATAGCAGAAATCCCC**TGCTTT** | -740 | 8.52 |
| 19 | Ava_0637 | - | TGTGAgtatttACACC | ATAAATTATTAAAAAATAATTAAAA**AAACAT** | -326 | 8.50 |
| 20 | Ava_1399 Ava_1400 Ava_1401 | - - - | CGTGTgtaaacTCACA | TATTGTATGCTCATGAATTT**TCAACT**TTGAA | -308 | 8.45 |
| 21 | Ava_4916 Ava_4915 | - - | GGTGAaattttCCTCA | CCGATTCCACAGGATTTCCACAGG**GATTTT**C | -166 | 8.44 |
| 22 | Ava_2093 | - | TGTGTatatttACACC | TAGCATAGAATAATTTATTTGACCA**AAAACT** | -53 | 8.43 |
| 23 | Ava_4451 | - | TGTGCcatagtTCACC | TAAGATTAGTTGCCAAAGCTTTTTT**TAATCT** | -79 | 8.43 |
| 24 | Ava_1358 Ava_1357 | - - | AGTGCctgattACACC | AATACCCATCACAACCACA**AATCCT**TACCAG | -263 | 8.43 |
| 25 | Ava_0687 Ava_0688 | - - | GGTGTcatcacCCACC | CTTACAAAAGTGTCAAGAC**GACAAT**TAATAC | -242 | 8.38 |
| 26 | Ava_2711 | - | GGTGTgagcgaTCGCC | ATAAATATCTTGTAATACCTGAGA**AATACT**A | -242 | 8.36 |
| 27 | Ava_1074 Ava_1075 Ava_1076 Ava_1077 | - - - - | AGTGTctatctTCACA | TCTACATCAGAAGATATT**TCATTT**TGGGAAT | -127 | 8.34 |
| 28 | Ava_4669 | - | GGTGTtgtcaaTCACA | TCAAGTTTTTGGTAAGCAATTTC**TACTCT**AA | -273 | 8.33 |
| 29 | Ava_2212 | - | TGAGTtgttcaACACA | GCGATCGCTACAATCTAGGGTAAA**TAAAAT**T | -45 | 8.31 |
| 30 | Ava_1543 | - | GGTGAttttgcCCACA | TAATTAACTTTCAAAATGTG**TACGTT**ACGTA | -61 | 8.29 |
| 31 | Ava_4510 | - | GGCGGtgttcaCCACT | ACAATCTTGGGAAAGCCTATC**TAAACT**GGAA | -104 | 8.26 |
| 32 | Ava_1395 | - | AGAGAaggacaTCTCT | CAGTCCTAGCCCGTCAGC**TAACTT**CGTAGGC | -648 | 8.25 |
| 33 | Ava_2543 Ava_2544 | - - | CGTGAttgtccTCACC | TAAAAGGCAGAAATAATTGA**TTTTGT**CTATA | -529 | 8.20 |
| 34 | Ava_3636 Ava_3637 | - - | TGTGAttgttaTCACA | TTATTTTTCTGAATAACC**TGTATT**TACGAGA | -430 | 8.19 |
| 35 | Ava_3543 | - | AGCGAtcgcatTCACC | CAATCGGGTGAACCATTGAT**TCTTCT**GTCAA | -395 | 8.16 |
| 36 | Ava_0854 Ava_0853 Ava_0852 Ava_0851 Ava_0850 Ava_0849 Ava_0848 Ava_0847 Ava_0846 Ava_0845 Ava_0844 Ava_0843 Ava_0842 Ava_0841 Ava_0840 Ava_0839 Ava_0838 Ava_0837 | - - - - - - - - - - - - - - - - - lpxD | AGTGTatatacTCTCT | AAAATGTAAAGTTTTTATAAAGAG**TGGAAT**G | -181 | 8.15 |
| 37 | Ava_0174 | - | AGTGAattactACACG | GAAACCCCACTGAAAAAAT**TATAAT**CTTTAC | -122 | 8.14 |
| 38 | Ava_2300 | - | CGTGGaaataaTCACT | AGACAGATGGTATTCAGTTCTTC**AAACTT**AA | -388 | 8.14 |
| 39 | Ava_2299 | - | TGTGAgagcgaTCGCC | GAAACTAACACTCAACCCA**TAACAT**ATGACC | -518 | 8.14 |
| 40 | Ava_2736 | - | AGTGTggtgatTCACA | AACGATTTTAGAGATTTTA**TCTCAT**AAAACC | -87 | 8.13 |
| 41 | Ava_4108 | - | AGTGCataattTCACG | ATGTTGATCCGTAATATA**TAATAT**CGCTGAT | -695 | 8.13 |
| 42 | Ava_4402 Ava_4403 | - - | TGTGTtataaaACTCA | CAGAAATTTATCTTAGCGGAAAGC**TGCTCT**A | -32 | 8.11 |
| 43 | Ava_2266 | - | AGTGTaatttaTTACA | TATAAGCCAATTGTAAAATTAT**TACACT**AAA | -180 | 8.11 |
| 44 | Ava_4668 | - | TGTGAttgacaACACC | CATCCTACAGATATTACTAA**TATCTT**CAATC | -234 | 8.11 |
| 45 | Ava_1542 | - | TGTGGgcaaaaTCACC | TATATTTCGTCCATGAATACAG**TAATGT**TTG | -175 | 8.11 |
| 46 | Ava_2257 Ava_2258 | - - | GGGGAactcaaACACA | AGCGTGATGTAGACGTTTTGTGTC**TCAATT**A | -76 | 8.10 |
| 47 | Ava_4815 Ava_4816 | - - | AGTGGcaaataTTACC | TAGTTGTTGATAATGGGCTA**TATGCT**AATAA | -189 | 8.10 |
| 48 | Ava_2698 | - | GGTGAaaaataACACC | TAATTATCTTTAATTAAGATATAG**AAATAT**C | -140 | 8.09 |
| 49 | Ava_4485 | - | CCTGAtaaaatTCACA | ATTTATGATGTTGGTTAT**TATTTT**TAATCTA | -679 | 8.08 |
| 50 | Ava_3667 | - | AGTGGccacatCCCCG | ACTTCTTCAAGAAGTCGGGGC**TATAGT**CGGG | -135 | 8.08 |
| 51 | Ava_2797 | - | TGTGTtttcaaTTACT | GCATTGGTTATTTTTTTAAATA**TAAAAT**GTT | -140 | 8.08 |
| 52 | Ava_2774 Ava_2773 | - - | GATGAtcctgaTCACA | TACTGAATTAGTTAATACCAGC**TATACT**TAA | -76 | 8.06 |
| 53 | Ava_4340 | - | CGTGTttttcaTCACT | TCGTCAAATATAAAAATCAAT**TAGTAT**AGAA | -35 | 8.06 |
| 54 | Ava_0223 | - | TGTGAttaactCCACT | GACGAAATAAAAGCATTCAG**TAGAAT**TTTCC | -426 | 8.05 |
| 55 | Ava_2630 | - | GGTGTgcattaCCACC | AATCAAAATCTATAAAGA**TAGGTG**GTGAAAA | -33 | 8.05 |
| 56 | Ava_4995 | - | GGTGAtttaatTCTCT | AAACCATTGACTACATTTGTAGCAC**TAATGT** | -81 | 8.05 |
| 57 | Ava_1491 Ava_1490 Ava_1489 Ava_1488 | - - - - | AGTGCgatcgcTCACC | ATATTAGCTACGATGGCACGA**TAAGAT**TCTA | -58 | 8.05 |
| 58 | Ava_2539 | - | TGTGTcctaacTAACT | CTATAATTTTCTAGGTCTAGAT**TAACTT**TGC | -57 | 8.04 |
| 59 | Ava_4547 | - | AGTGTtttccgCCTCC | GTAGAGTTTTTTGTATCTGTCT**TAGCAT**TGA | -132 | 8.02 |
| 60 | Ava_4728 | - | AGTGCctatttACACG | GACAAAATTACTAAGAAT**TAATTT**TTCCTAG | -130 | 8.02 |
| 61 | Ava_0236 Ava_0237 | - - | AGTGAtaaataTTACA | TTACCAGCTAAACCACATAAT**TATTTT**CTGA | -116 | 8.01 |
| 62 | Ava_0190 | - | CGTGTcaattgACACA | AAATGAGTCCTTATGGCTCTT**TTTCTT**TCCT | -401 | 8.01 |
| 63 | Ava_0613 | - | AGTGGcaactgACACG | CGCAGCACAAGGAAAATATTTGG**TATTTT**AT | -174 | 8.00 |
| 64 | Ava_0051 Ava_0052 | - - | TGTGCcgccttTCCCC | GAACAGCACCCATAACAATTCAAAA**TACCCT** | -327 | 7.99 |
| 65 | Ava_1547 | - | TGTGAattaatTCTCT | CGAAAGATTAATTTTTCTCAAAA**TACTGT**AA | -278 | 7.99 |
| 66 | Ava_2627 Ava_2626 | - - | AGTGTgattcaGCACT | GACGGGCTTTTTGCTTAGG**TTTGTT**CTACAT | -38 | 7.98 |
| 67 | Ava_3143 Ava_3142 | - - | AGTGAtcaggcACACT | TTAGCCAGTTCTATTGCTTTT**TAAGTT**CTGT | -600 | 7.98 |
| 68 | Ava_2196 | - | TGTGAttttgcACACT | ATAAGTGTTAATTTAAGAATACTGG**TGTATT** | -61 | 7.98 |
| 69 | Ava_1431 Ava_1432 Ava_1433 | - - - | AGTGAggacttACACA | ACTGGCAGATTCGTATGGTGTG**TCAGAT**TTG | -333 | 7.98 |
| 70 | Ava_2547 | - | TGTGAgtaataACTCA | CACTAGCAAGGTTCAGAC**TGGGCT**TACAAGC | -56 | 7.98 |
| 71 | Ava_2679 Ava_2678 Ava_2677 | - - - | AGTGCggtcttACACT | GGCAAGATTGGTAGCTTGCTT**TAACAT**AAAT | -61 | 7.96 |
| 72 | Ava_4190 | - | CGAGAaaatttTCACA | CCGCTTTAAGTCTACTCC**CATGTT**TAGCAAT | -117 | 7.96 |
| 73 | Ava_4753 Ava_4754 | - - | GGTGCttgttaTTACA | GGTATCCCTTCGTCTTTTTGCGGC**TATAAT**A | -513 | 7.95 |
| 74 | Ava_1559 | - | TGCGCctgcatCCACC | GATAGAAATAATAAATCTATT**TTTTTT**GGGC | -327 | 7.94 |
| 75 | Ava_4571 | - | CGGGAtgggttGCACT | ATCGCTCTACTCATCCCACTATA**AATGTT**GC | -261 | 7.94 |
| 76 | Ava_2035 | - | GGTGAcaatatTCTCA | AAATAAGATGTTGATAAA**TAATGT**ACATCTG | -212 | 7.93 |
| 77 | Ava_2307 | - | TGTGCaaacttCCACA | CATCGCTTGAGAATTTGATTTTC**TATTGT**CA | -404 | 7.93 |
| 78 | Ava_1928 | - | TGTGCttcgccTCTCT | ATTTTCGACTTGTCTAGC**CATCCT**TGCAAGT | -161 | 7.92 |
| 79 | Ava_0990 Ava_0991 Ava_0992 | - - - | TGTGCaaaagtACTCT | TGCACCGTTGTTAAGTTTGG**TATCTT**TCTGA | -62 | 7.92 |
| 80 | Ava_3116 | - | TGTGCatttcgGCTCA | CACTTGTTTTATACAGAGAAGCAGT**TATACT** | -124 | 7.91 |
| 81 | Ava_3027 Ava_3028 | - - | GGTGAagctccTCACT | CTTACTTAGAGTAAATCTTGAAAGT**TCATTT** | -83 | 7.91 |
| 82 | Ava_4031 Ava_4030 | - - | TGTGGaagcgaTCGCC | CAGATTAAAATGAAAGGGTGATATA**TAAGTT** | -40 | 7.89 |
| 83 | Ava_3832 | - | GGTGTtcaaccGGACA | CGATATAATTTCTAGTCCC**TATGCT**CATGAA | -49 | 7.88 |
| 84 | Ava_4994 | - | AGAGAattaaaTCACC | CATTTGGGGGAGAAAGACAGGATT**TAGGGT**T | -195 | 7.88 |
| 85 | Ava_4773 Ava_4774 Ava_4775 | - - - | AGCGTatctatTCGCC | GAAGCTCCAATTTATTCC**TAGCAT**TATTGGG | -199 | 7.88 |
| 86 | Ava_0981 Ava_0982 Ava_0983 | - - - | TGCGCcttaaaCCACT | CGGCCATCTCTCCAGATGTCACGA**TTTACT**A | -121 | 7.87 |
| 87 | Ava_0669 | - | AGTGGttcaatTCACA | AGCAAGTTATCATCATCAAG**TAGTTT**TAAAC | -40 | 7.87 |
| 88 | Ava_4863 Ava_4862 | - - | AGTGAatttccTCATT | TCTTTTGCTGAGTTATTATGTAACA**TAATAT** | -188 | 7.86 |
| 89 | Ava_3729 | - | AGCGAaaataaTCACC | GCCGATACACGCCAATGAACGCTGA**TAAATG** | -316 | 7.86 |
| 90 | Ava_3899 | - | TGTGAaggcgaTCGCT | CTTGGCGGTTCCTATGAATGA**TAATTT**TAAG | -61 | 7.84 |
| 91 | Ava_4709 Ava_4710 | - - | GGTGTctaaagCCACG | TACAATTAATATCACTTAACT**TTTTTT**ATCA | -45 | 7.82 |
| 92 | Ava_2972 Ava_2971 Ava_2970 | - - - | AGTGTtttaaaACACA | TCTTGCGTAACAATTTAG**TATGTT**ATAACAC | -136 | 7.82 |
| 93 | Ava_3330 Ava_3331 Ava_3332 Ava_3333 | - - - - | GGTGCagcattACACT | GTTCTGGGAGCATATACCTT**TAAAGT**AGTGA | -141 | 7.82 |
| 94 | Ava_1629 Ava_1628 Ava_1627 Ava_1626 | - - - - | GGTGAgcaatgCCACC | CTACTAATACTTGATGTT**TGCATT**GCCTGTT | -465 | 7.82 |
| 95 | Ava_3542 Ava_3541 | - - | GGTGAatgcgaTCGCT | CCAGTAGGAATTAACCTGAAG**TAAGAA**GCAG | -94 | 7.81 |
| 96 | Ava_1496 Ava_1495 Ava_1494 | - - - | GGTGAtttctcGCTCT | ACCTCTAGGTTTTGACAGAAATGC**AATATT**C | -201 | 7.80 |
| 97 | Ava_4903 Ava_4904 Ava_4905 | - - - | TGTGTtatgaaATACA | AATTTTATACTTAGTTTTG**TAGCAT**TTGCCC | -130 | 7.80 |
| 98 | Ava_4740 | - | CGAGAagttttACACA | AGTGACAAAAGATATGTAATTTA**TACACT**GA | -141 | 7.79 |
| 99 | Ava_3399 | moaC | CGTGCtctagtCCACT | GAGCTACACGCCCTTACAAGAAATA**TATACT** | -52 | 7.79 |
| 100 | Ava_3590 | - | AGTGTtaggatGCCCA | AATTTATGTTTGTACATTGCTG**TATTAT**TGG | -62 | 7.79 |
| 101 | Ava_0573 | uvsE | TGTGActgttgACAAA | AATGCTGCTGTGTACAGGT**TATTTT**AGGTGA | -283 | 7.78 |
| 102 | Ava_4977 Ava_4978 Ava_4979 Ava_4980 | - - - - | GGTGAgagcagTCACA | GTTTGCACAAGCAATAGCG**GAACGT**CTTGCA | -206 | 7.78 |
| 103 | Ava_1482 | - | GGTGTgaatcaACACT | CTCGATAGTGGACACCAATTTAGT**TATACT**A | -49 | 7.77 |
| 104 | Ava_1770 | - | ATTGGtcgcaaTCACA | TAACCTTGATAAGAAACTAC**TATGCT**GATGG | -129 | 7.76 |
| 105 | Ava_4060 | - | TGGGAtttaaaTCACC | AACTCCAACCGTCTTTAATTT**TGAATT**TTGT | -117 | 7.76 |
| 106 | Ava_2661 | - | AGCGAtcgcctACACC | TACACTATCCCTAAAAGCCCTATA**AAAATT**A | -600 | 7.76 |
| 107 | Ava_0387 | - | AGTGGattgctTCCCC | ATATTCTTATAGTCTGACA**TATTTT**TCTGTT | -136 | 7.76 |
| 108 | Ava_4315 | - | TGTGGcctcaaTCACC | TAACTTAGAGTTATGCTAATT**TTTATT**AAGA | -55 | 7.75 |
| 109 | Ava_4727 | - | CGTGTaaatagGCACT | GGAACTTTACTGAATTCAGATTAA**GAGAAT**A | -237 | 7.75 |
| 110 | Ava_1736 | - | AGCGTgcgtagTCGCA | AGCATCCTTCACCGTATT**TTTTCT**CACTGCT | -39 | 7.75 |
| 111 | Ava_4909 | - | AGAGTaatcacTCTCC | CGCCTAGCCTTCGGCGAAGTCA**TAGGCT**ACT | -58 | 7.75 |
| 112 | Ava_2848 Ava_2847 | - - | TGTGGtgttaaATACA | ACTGATTATCACTACAAATTTATG**TATACT**A | -208 | 7.74 |
| 113 | Ava_2333 | - | GGCGAtcgccaTCACA | GCATAAATTTAGCTGCGAC**TATGAT**TTCGTA | -78 | 7.74 |
| 114 | Ava_1149 | - | GGTGGcttttcTCCCT | ACCAATGATGAGTTAGGCTTTCCC**TCTACT**T | -87 | 7.72 |
| 115 | Ava_2723 | metG | TGTGTtcttcaTCACC | ATAAATGCAACGAAAACATTATCC**AATATT**A | -224 | 7.71 |
| 116 | Ava_3170 | - | GGTGGagctttTCTCT | GTTTTTCAGTCCTCAGAACTCTGA**TTTTCT**A | -46 | 7.71 |
| 117 | Ava_1558 | - | GGTGGatgcagGCGCA | TCAAGATATCATCTATCTT**TGGTTT**GGGAAA | -97 | 7.71 |
| 118 | Ava_2973 | - | TGTGTtttaaaACACT | ATTTGTGAATCGGGACTGAATAA**AAACTT**AT | -134 | 7.71 |
| 119 | Ava_1172 Ava_1173 Ava_1174 | - - pyrB | TGGGAactgctTGACA | CAAATTGTCATAGTAGTTAAATT**TACATT**AA | -283 | 7.70 |
| 120 | Ava_2912 | - | GGCGAtcgctaGCACT | GCTAAGAATCGTTATCTAAGTAAGG**TAAGAT** | -260 | 7.70 |
| 121 | Ava_4490 | - | GGTGTtcaaatTCCCT | ACACTTTCCCAGATTCCAAT**TATCAT**AGTTC | -415 | 7.69 |
| 122 | Ava_1008 Ava_1009 | - - | TGTGTattttcTCACC | AAAATCTTCGCCACCATT**TCTACT**ACCAGTA | -87 | 7.69 |
| 123 | Ava_0430 Ava_0429 | - - | AGTGCgatagaTCCCA | ACTATACTCAGAAATTATT**CATACT**GACGCA | -113 | 7.69 |
| 124 | Ava_0624 | - | TGAGCatttctGCACC | ATCTCCTATTTTCTGCTAAA**TTTTAT**GAAGC | -304 | 7.68 |
| 125 | Ava_2428 | - | AGTGActttttTCTCT | CTCTAGGTTGAGAAAATCTGAGT**TTTGTT**TC | -367 | 7.68 |
| 126 | Ava_1429 | - | TGTCAtcaattACACA | CTTTTCCCAAAAGCTCCAGA**TAGAAT**CATGA | -310 | 7.68 |
| 127 | Ava_3207 | - | GGTGAatttacTGGCA | ATGAACATTCTTATGATTGTTC**TATTGT**GTA | -544 | 7.67 |
| 128 | Ava_1920 Ava_1921 Ava_1922 | - ppnK - | TGTGGctgcatTCACC | GGAAAACTTTGTTTTAGCGA**TTTTTT**CAGTA | -127 | 7.67 |
| 129 | Ava_4339 | - | TGTGTtcttaaCCACC | TCACATCCGGCACATTAAAG**TTTTGT**CAAGA | -472 | 7.67 |
| 130 | Ava_0920 | - | TGTGAacctctTTACA | TCTTCAAGTTGGGAAACCCACCCT**TATCTT**A | -168 | 7.66 |
| 131 | Ava_1951 | - | TGAGAtttttcACTCA | TACGATCGCCTGGTAACA**TTATTT**GCGACTA | -259 | 7.65 |
| 132 | Ava_2808 | - | GGTGAtggcaaTGACA | CAATTATCGGTAATAGTGCTGC**TAACAT**TTT | -158 | 7.65 |
| 133 | Ava_2648 | - | TGTGCcagtttTTACT | TATTTTTATTAGTTAAAAAT**TACTTT**CTTGA | -303 | 7.65 |
| 134 | Ava_4857 | - | CGTGCggatttTCCCA | GCATTTAGGAATTTTTGGG**TATTTT**TTT--- | -29 | 7.64 |
| 135 | Ava_2256 | - | TGTGTttgagtTCCCC | TCTCATCCTTTATACCGTTAGTAAG**AATAAT** | -557 | 7.64 |
| 136 | Ava_2629 | - | TGTGTataaatACCCT | TTCCCGAACCAAGGGACTG**TGTTAT**AAGAAG | -85 | 7.63 |
| 137 | Ava_0308 | - | GGTGAaagcttGCGCC | GTAATCCTTGGTTCAATAGG**TATGGT**TAGGG | -732 | 7.63 |
| 138 | Ava_1359 | - | GGTGTaatcagGCACT | AATATTGTGTTAATAAATG**TATGGC**TAGTAG | -80 | 7.63 |
| 139 | Ava_3710 Ava_3711 | - - | AGTGAgagcgaTCGCT | AAGATGATAAAAGATTCACTCGCTC**TCTAAT** | -41 | 7.63 |
| 140 | Ava_2083 | - | TGTGGacaattTAACA | TTTATGGTCTTGAATATTATCATCA**GAATAT** | -172 | 7.63 |
| 141 | Ava_0682 | cls | CGTGAtatccaTCGCA | AAGAATCCATTTGTTTGAGAT**TGTCTT**GGCT | -750 | 7.63 |
| 142 | Ava_3947 | - | AGTGAactttaACTCA | CCATTCTTTTCTTCTAGCTTTGC**TTTTTT**GT | -444 | 7.62 |
| 143 | Ava_3610 | - | TGTGAagattaCCGCA | AATTCCACCCTAGTACAGA**TGTCTT**CACTAA | -258 | 7.62 |
| 144 | Ava_2423 | - | CGTGTttagatAGACT | CGGCTTCAAGGGATAGAGGCTTGG**TAGGAT**C | -75 | 7.62 |
| 145 | Ava_3867 | - | GGTGGgattttTCCCA | AGCAGTACATTACTGGATGGG**TAAGCC**ATTT | -118 | 7.61 |
| 146 | Ava_0874 Ava_0875 Ava_0876 | - - - | TGTGGggcgtaTCTCA | CAGAGAGTGTCAACTAAAGAGTC**AAGAGT**TA | -247 | 7.60 |
| 147 | Ava_4967 | - | CGTGTcttttgACACA | ATTTTACAAAAAAAGTAAC**AATAAT**ACTTAG | -59 | 7.60 |
| 148 | Ava_1471 | - | TGTGAtctctcCCACT | AGAGTTGGGAATAATAAAATCAA**CCACAT**CC | -145 | 7.60 |
| 149 | Ava_2609 | - | CGAGAggagacTCACT | CTCGACCTGAATCACAGAG**TAGCGT**TCGCCA | -98 | 7.59 |
| 150 | Ava_3265 | - | TGTGTctaattTCACT | TATGTCTGTAGATTTTGAG**TTCCCT**ATTCAG | -370 | 7.58 |
| 151 | Ava_0492 | - | TGTGAgttggtTCTCT | ATTATTTGATGAACTTATAGGTAAC**AAAAAT** | -116 | 7.58 |
| 152 | Ava_3643 | - | TGTGGgcgcgaTCGCC | TGCAATATCGACATATTAGGTTTA**AAAATT**A | -272 | 7.58 |
| 153 | Ava_0942 Ava_0943 | - - | TGTGTtttttcTGACA | ACTTATTTATAATTGCTA**TATTAT**CAATTTA | -90 | 7.57 |
| 154 | Ava_0819 Ava_0820 Ava_0821 | - - - | AGTTAaaaattTCACA | AATGTTTTAAATAGCTACG**TACATT**TATTGA | -70 | 7.57 |
| 155 | Ava_2826 | - | GGTGGagggtaGCCCA | ATCGCTGTTAGTTTAATAATGAAT**TACTCT**A | -40 | 7.57 |
| 156 | Ava_1293 | - | AGTGCtagctaTCACC | TAAGGTGGGGCATAGCCCA**TCGCAT**ATCAAC | -458 | 7.57 |
| 157 | Ava_0354 Ava_0353 Ava_0352 | - - - | AGTGGctagaaGCACC | TCGGAACGGGAATTAAGGAAC**TTCCAC**---- | -28 | 7.57 |
| 158 | Ava_4503 Ava_4504 | - - | GGGGAtgaagtTCTCA | GATTATGTTGCCCAAATCGATTA**TAGGGT**TT | -134 | 7.57 |
| 159 | Ava_2796 | - | GGTGGttggagCCACC | GTTTTTTATTGACGTTACA**AAAGTT**TATGCC | -223 | 7.57 |
| 160 | Ava_1106 Ava_1107 Ava_1108 | - - - | AGGGTgtgttaTCACT | TAGGGTAACGCACCCTACGTG**TATTGT**AAAA | -322 | 7.57 |
| 161 | Ava_4379 Ava_4380 | - - | AGTGAcatccgCCTCA | TCTCCTTCATCTTCCTCATTCCT**TCATAT**CC | -61 | 7.56 |
| 162 | Ava_0284 | - | TGTAAttaaagCCACA | TTTATCTCGTTTTACGGCATCAG**TATTAT**CA | -119 | 7.55 |
| 163 | Ava_1895 | - | GGTGAatttatACTCA | TTATTTAATGTTTAGTTAC**TACTCT**TATTGC | -307 | 7.55 |
| 164 | Ava_1737 Ava_1738 Ava_1739 | - - - | TGCGActacgcACGCT | TTGCGGGAAGCTTCCTATAGTGG**TCAAAT**AG | -52 | 7.55 |
| 165 | Ava_1021 | - | TGCGTaatactGCACC | AGTAGCTAGTTTTAGTGGATGATC**TAAATT**T | -709 | 7.54 |
| 166 | Ava_0294 | - | TGTGAgtcaatACCCT | GACCTTTACTCAGACAATTTCC**TATTTT**GCC | -185 | 7.54 |
| 167 | Ava_3009 Ava_3010 | - - | TGTGAgctaatATACA | TAGAATCGTGTTTGCATTTTTTGC**AAAACT**A | -58 | 7.54 |
| 168 | Ava_2277 | - | TGTGGcttgctTCCCG | TGGGGTCTTTTGAATTGG**TATGAT**GCACCAG | -77 | 7.54 |
| 169 | Ava_1470 | - | AGTGGgagagaTCACA | TTTTGATAAATGTTATTGCCAT**TACCCA**GGA | -48 | 7.53 |
| 170 | Ava_0980 | - | AGTGGtttaagGCGCA | GCACTGGAAATGCTGTAATGCGG**AAACGT**AT | -534 | 7.52 |
| 171 | Ava_4186 | - | TGGGCtacgccACACC | ATAGGTAAAGCAGTCTAAAAA**AACATT**AATA | -463 | 7.52 |
| 172 | Ava_1386 | - | GGTGTttattaCAACT | AAAAAATCTTTTGGGTGCTT**TAGAGT**TTAGG | -368 | 7.51 |
| 173 | Ava_4130 | - | TGTGTtagctgTCGCA | ATGCTACAAACTATTTGT**TAGTCA**GGAGTAG | -36 | 7.51 |
| 174 | Ava_0640 Ava_0639 | - - | AGTGGcaaactCCTCT | TGTAGCCAGGTTTTATCTTAACA**TATCTT**AT | -176 | 7.51 |
| 175 | Ava_3666 | - | CGGGGatgtggCCACT | GATAATACATAACAGGAATTTAAA**AAACTT**C | -202 | 7.50 |
| 176 | Ava_4536 | - | CGTGAtctttcTCTCC | CGTCAAATACCATAAATAGTCAT**CATTAT**GG | -283 | 7.50 |
| 177 | Ava_4399 | - | GGAGAacccgtTCGCG | TTAGCGTTGCGTAGCAAAGGG**TATTTT**GTAG | -162 | 7.50 |
| 178 | Ava_3654 Ava_3655 Ava_3656 Ava_3657 | - - - - | TGAGAtgatggACGCA | AAAATTATACTCTTAATGGTTTCAA**CAACTT** | -79 | 7.50 |
| 179 | Ava_1734 Ava_1735 | aat - | AGTGCagaggaGCGCC | AAGTATGAGTAACAACATCAGAG**CGCGTT**CC | -62 | 7.50 |
| 180 | Ava_4493 | - | TGTGCtattacACTCC | ATTAAAACTATGATGTAGGCA**TAAATC**GCCC | -266 | 7.49 |
| 181 | Ava_1007 | - | GGTGAgaaaatACACA | GGTCAATTTAAAATTTAAATTCCG**GACAAT**T | -52 | 7.49 |
| 182 | Ava_4478 | - | AGTGAacattgCCTCT | CTTCGCTAGTATTACGAGCCAGTG**AAAAGT**A | -776 | 7.49 |
| 183 | Ava_2724 | - | GGTGAtgaagaACACA | AATCTTCCAAACTACGGAATTTAGC**GGCTTT** | -142 | 7.49 |
| 184 | Ava_0154 Ava_0155 | - - | TGCGTagcccaTCGCA | CAGAAGATTGTCTGTATTT**TAACCT**CACTAT | -180 | 7.49 |
| 185 | Ava_1679 | - | TGTAAaatctcTCACT | TTGAAAATTATCATGATCAGT**TATTAT**ATGA | -205 | 7.48 |
| 186 | Ava_2747 Ava_2746 | - - | GGCGAcaaattGCACT | CCTAACATGATATTGGTAT**TACAGC**GACTTA | -88 | 7.47 |
| 187 | Ava_1566 | - | TTTGAgtatttTCACT | GTACATTATCAACTCGAT**TATAGT**TAAGTTG | -81 | 7.47 |
| 188 | Ava_4427 | - | TGTGTacacttTCGCC | CTTCTCGCAACTTCACCTCCGTTA**GAGACT**C | -142 | 7.47 |
| 189 | Ava_1202 | - | GGTGAggcgtaAGCCC | TTGCTGAGTAAAAATTCTAGCCTC**TAGCCT**C | -125 | 7.47 |
| 190 | Ava_0552 | - | GGTGTaaaaaaTGACG | ACACTACTGTTAAAAGAGCATCAAA**CAGCTT** | -124 | 7.47 |
| 191 | Ava_1553 | - | AGAGTattaaaCCACA | TCAAAAACAATTTAGAGACAA**TATACT**TATT | -371 | 7.47 |
| 192 | Ava_4892 | - | CGGGAtttaaaTCCCC | GTCTGAACGCGTACCGTAGGCAAAA**TGTATT** | -99 | 7.47 |
| 193 | Ava_2234 | - | TGTGTttggaaTCGCC | AGAAAAGAGCGATCGCCAAAAT**TATAGT**CGG | -339 | 7.46 |
| 194 | Ava_0574 | - | TTTGTcaacagTCACA | AACTGTACCAGCAAAATAA**TAGGGT**AATA-- | -30 | 7.46 |
| 195 | Ava_1294 | - | GGTGAtagctaGCACT | CAGAAATACGCTTTGCTGGA**AAAATT**AAAAC | -283 | 7.46 |
| 196 | Ava_2168 Ava_2167 | - - | AGTGTtgggttACGCA | AAGCCTCCACCCAACCTACATTT**TAGGCT**TG | -110 | 7.45 |
| 197 | Ava_1080 Ava_1081 Ava_1082 | - - - | AGTGGaaatcaTCCCG | AACCTCTACCCCAGAAAATGCAA**TTAATT**AA | -380 | 7.45 |
| 198 | Ava_2751 | - | TGAGTtatcggTCTCA | ACGAAAATTATAAATAGGCAATAG**CAAAAT**A | -136 | 7.45 |
| 199 | Ava_2524 | - | AGGGCaaaataTCACA | AGCAAAGTCGAACTAAATT**TCACTT**TAGGCG | -110 | 7.44 |
| 200 | Ava_2427 | - | TGTGCttttgcTCCCA | CTTGCAAATAGCAAGCTTACG**CATTTT**CACC | -308 | 7.44 |
| 201 | Ava_4920 Ava_4921 | - - | GGCGAtttttaACCCT | ATCCTGAGATAGAGCTTTT**TTTCGT**TGCCCA | -49 | 7.44 |
| 202 | Ava_2227 | - | TGCGAtcgctaGCACA | GGCTCACACTCATATAAAATATCAC**TATCAT** | -148 | 7.44 |
| 203 | Ava_3045 | - | AGCGActtgacGCACC | CTACCAAACCTGTTGACTGTTGAC**TATATT**T | -54 | 7.43 |
| 204 | Ava_3570 Ava_3569 | - - | AGTGCttaaaaTCACA | GCCAATCACTTTTCAAACAA**CCTCTT**AGGGG | -592 | 7.43 |
| 205 | Ava_4639 Ava_4640 Ava_4641 | - - - | AGGGTcaaaagACACT | ACTCTGCAAAAGTAGCCCATT**TAACTT**TAGA | -351 | 7.43 |
| 206 | Ava_0370 | - | TGTGActaaatGCTCA | CTAACGGGAGATAATAATA**AATCCT**CACCAC | -114 | 7.42 |
| 207 | Ava_2662 | - | GGTGGgtattaCCACA | CCCTACAGATACTTAGAT**TCTTGT**ACGCAAG | -276 | 7.42 |
| 208 | Ava_3837 | - | TGTGAcaatttTGTCA | CAATTTAGGGAAATTTAGGC**AAAGTT**TTTCA | -203 | 7.42 |
| 209 | Ava_1959 Ava_1958 | - - | AGTGAccaataTGGCC | TAATGTTTTGCCCTAGTAT**TCTGTT**GAGTAA | -122 | 7.42 |
| 210 | Ava_4988 | - | TGTGAcagattCCCCA | AAAGCGGTGATTACAGTAATTTTGG**GAACTT** | -161 | 7.42 |
| 211 | Ava_0462 | - | GGGGAtagtacCCACA | TCAAGACTGGGGCTGATGTTTG**TAGTAT**ATG | -55 | 7.41 |

**Table S5**. Predicted CRP binding sites in *Synechococcus sp.* JA-3-3Ab genome at *P* < 0.01

| Rank | Transcription Unit | Names | CRP TFBS | Downstream region of the CRP TFBS | CRP site position | Score |
| --- | --- | --- | --- | --- | --- | --- |
| 1 | CYA_0127 | - | CGTGAtccggaTCACT | TCGGGAGGGGGGGGAAACGGCA**GAGACT**GGA | -253 | 7.76 |
| 2 | CYA_0977 CYA_0976 | - tas | AGAGAtacggaTCACA | TCTTATCCATCCTTTGCCGGTC**TAGAGT**GAA | -180 | 7.67 |
| 3 | CYA_2314 CYA_2313 CYA_2312 | - - - | TGTGAtctcgtTCCCT | GTGTTTTTTCTCTAAGCTCTGCAA**AATAGT**A | -249 | 7.48 |
| 4 | CYA_0705 | - | GGTGAgcctgaCCTCA | ATTCTACCGCCTTGTCTTAGGA**TCCCGT**TCC | -156 | 7.12 |
| 5 | CYA_1135 CYA_1134 CYA_1133 CYA_1132 | rpmF - - - | GGTGAccggcaTCACC | TGGGCGCCTGTTCCCAGAAGA**TGTCCT**ATAC | -239 | 7.11 |
| 6 | CYA_0687 CYA_0688 CYA_0689 | - - - | AGTGTatggctTCGCC | ATGCAAGCCATAGCCATTCCAAAC**TGGATT**G | -199 | 7.10 |
| 7 | CYA_2527 | - | GGTGAtggtggGCACC | CGCAGCAGGCGGCGATTGCCAG**TAGCCT**CGG | -776 | 6.91 |
| 8 | CYA_2183 | - | AGTGCtccagaAAACA | GACAATTCTTGGACAGTATGT**TAAGTT**CAAG | -284 | 6.90 |
| 9 | CYA_2632 | - | AGGGAtccagaTCACT | GGCGGCTTCACAGCTTTATC**TAAGCT**TGATA | -85 | 6.89 |
| 10 | CYA_2315 | - | AGGGAacgagaTCACA | GCCACGGAAGACTTGAAGGGA**TAGCAT**AAAC | -179 | 6.86 |
| 11 | CYA_0978 | - | TGTGAtccgtaTCTCT | TTGTCCAATGGAAGTCTCTAAAGCA**GAATCT** | -300 | 6.77 |
| 12 | CYA_0295 CYA_0294 CYA_0293 | psaF psaJ psaL-2 | TGTGAgctaaaACTCG | CAATCCGTTGGAGCGAGGTACTCGG**GAGTAT** | -119 | 6.76 |
| 13 | CYA_1696 | - | CGTGAaaacagTCACG | CGGGGAATAGATGTTTTTTGTA**AAACAT**CTA | -75 | 6.75 |
| 14 | CYA_0192 CYA_0193 CYA_0194 CYA_0196 | - dxr - murE | TGTGGgcaaaaCCACC | ACGGCGGTACACCTGGCCCTC**TACCTT**CAAC | -572 | 6.74 |
| 15 | CYA_0696 CYA_0697 | - - | TGTGGcaagtgTCTCT | AGCTTAGCTGAACGTAAGACGATG**TATGCC**C | -407 | 6.71 |
| 16 | CYA_0900 CYA_0899 | - - | AGTGGcgaggaTCCCT | TGGAGAGTGTCTTATATCTGGTT**TAAAGT**GG | -198 | 6.69 |
| 17 | CYA_1742 | - | TGTGTtcctctTCTCA | GCGATTGCCCCAACATTGG**ACTCAT**CGAGCT | -116 | 6.65 |
| 18 | CYA_2375 | - | TGTGAacctgaCCTCT | GGTTCTCCTGCTCCGGTGGT**TCTGCT**AGCGA | -118 | 6.64 |
| 19 | CYA_1290 | trpA-2 | GGTGAgctggcGCACC | GCTCTGGCTTATGATGCCACCCGGG**TTTTCT** | -294 | 6.64 |
| 20 | CYA_1585 CYA_1587 | - codA | AGTGAgcaataACCCA | ATCAACTGGTTTCCTAAATTCG**GGCTTT**GAG | -148 | 6.64 |
| 21 | CYA_0706 CYA_0704 | - - | GGTGAcgagacTTTCA | CTGAGTTCACGCAGAGGT**GAGCTT**GGTGAGT | -104 | 6.61 |
| 22 | CYA_1844 | - | CGTGAcgacttTCAGA | ACAGGAAAATCCACCAATCGCTCGG**TACTTT** | -221 | 6.61 |
| 23 | CYA_2120 CYA_2119 CYA_2118 CYA_2117 CYA_2116 CYA_2115 CYA_2114 CYA_2113 CYA_2112 | - atpB - atpE - atpF atpH atpA atpG | GGTGAcctcatTCCCA | TGGCACGTTCTTCCCGAC**TCCTCT**CCCACAG | -170 | 6.57 |
| 24 | CYA_1830 CYA_1831 CYA_1832 CYA_1833 CYA_1834 CYA_1835 CYA_1836 CYA_1837 CYA_1838 CYA_1839 CYA_1840 CYA_1841 CYA_1842 | - dpsA - - nifE nifN nifX - - nifW - - - | AGTGAagactaTTGCG | GCGGCAGGCCGTCGGACTT**TCTTGT**TGTGAC | -318 | 6.57 |
| 25 | CYA_0603 CYA_0604 CYA_0605 CYA_0606 CYA_0607 | ureC ureE ureF ureG - | GGTGGccttgaTCACG | GCGGAGGTACTGGAGGGTATTCGC**GAGGGT**A | -620 | 6.56 |
| 26 | CYA_1199 | - | TGTGTtccaaaATACA | AGCTTCTGGAAAATCTGCAGC**AAACCT**GGAT | -372 | 6.50 |
| 27 | CYA_2503 | - | GGAGAtcccggCCACA | GAAAAGCAGATCCTGACAGT**TATCCT**CGAAC | -92 | 6.50 |
| 28 | CYA_2608 | - | GGTGAtaaaaaTCTCT | CCCTTCTTAAGATTGAGTCGCAA**TAGCTG**GA | -83 | 6.48 |
| 29 | CYA_2487 CYA_2486 CYA_2485 CYA_2484 | secG - - ggt | AGTGAgagatcCCACG | CTGAAACTCCAATCTAATG**GATTTT**GGAAGG | -106 | 6.48 |
| 30 | CYA_1518 | - | AGTGCgggttcTCGCC | ATCTCAGCGCAAAGCTCACCA**TAGCTT**GCCC | -651 | 6.48 |
| 31 | CYA_0488 | - | AGTGGgctaccTCGCC | TCTAAAGCTGTGTTAAGATTTTGCG**TAACTT** | -91 | 6.46 |
| 32 | CYA_2644 CYA_2645 CYA_2646 | - ribE aroK | GGTGAatccctGCGCA | TTTGGACTGCAGAGGCTGGCCGG**GAGGAT**GT | -36 | 6.46 |
| 33 | CYA_0189 CYA_0188 | - rpsU | GGTGGttttgcCCACA | CCTCCTTTGAACCCCAAGACCGC**GATATT**CA | -294 | 6.44 |
| 34 | CYA_1527 CYA_1528 CYA_1529 CYA_1530 | - - - - | GGTGAtcttgaTCCCC | CAGGGATCGGTGGCCTCGTCCAG**TTCTGT**CA | -467 | 6.44 |
| 35 | CYA_2690 | - | GCTGAagccgaTCACA | GGGAATGGAGGCACTGAACTGTG**GAGAAT**GG | -37 | 6.43 |
| 36 | CYA_2669 | - | GGTGAggctgcTCACG | GTTAAAACGCGCCGCTGA**TCCCGT**ACCAGGG | -767 | 6.43 |
| 37 | CYA_2387 CYA_2386 CYA_2384 | ispG hemL - | AGTGAtcctgcTCCCC | TGGGGCAGTTGATTGGCTTGACT**TTTCTT**TA | -202 | 6.43 |
| 38 | CYA_1145 CYA_1147 | - - | AGAGAcatcgcTCACC | CCAGAGATGGGCTTTGGT**TTTCTT**CTCTACC | -88 | 6.43 |
| 39 | CYA_2795 CYA_2796 CYA_2797 | - - - | GGTGAgcttggTCAGC | TCCTCTTGGGCAGAGGTCGTCGGT**TAGTGT**T | -86 | 6.43 |
| 40 | CYA_2395 | fni | AGTGCatctctTCACA | CTCACCCGACACTTGAAGCAGCTC**TACGGC**G | -781 | 6.43 |
| 41 | CYA_2084 CYA_2085 CYA_2086 CYA_2087 | - - - - | TGTGTatacggACTCA | ATGGCACCGGGGAAGGTG**TAGCGT**CGCTGAG | -222 | 6.41 |

**Table S6**. Predicted CRP binding sites in *Synechococcus sp.* JA-2-3B'a(2-13) genome at *P* < 0.01

| Rank | Transcription Unit | Names | CRP TFBS | Downstream region of the CRP TFBS | CRP site position | Score |
| --- | --- | --- | --- | --- | --- | --- |
| 1 | CYB_2776 | - | AGTGAtctgaaTCACT | TCGAAATGGCAAAAAGCAACA**CAAACT**GAGG | -397 | 7.82 |
| 2 | CYB_2927 CYB_2928 | - tas | AGAGAtacagaTCACA | CTCATCCATCCTTCGCCGACT**TAGAGT**GAAC | -184 | 7.71 |
| 3 | CYB_0465 CYB_0464 CYB_0463 | - - - | GGTGAtcggttTCACC | GTCCAAGAGAACATTTTCTGGT**TAACTT**TGG | -112 | 7.61 |
| 4 | CYB_2029 CYB_2028 | - - | TGTGAtctcgtTCCCT | GTGGTTTTTCTCCAAGCTCTGCAA**AATAGT**G | -250 | 7.51 |
| 5 | CYB_1665 CYB_1666 CYB_1667 CYB_1669 | acpP fabF - - | GGTGAggctgcTCACA | AGAGGGGATCCATTGTCCGA**TACAAT**AGGGA | -52 | 7.51 |
| 6 | CYB_1142 | - | CGTGTtcggtaACACA | GAAATCTTGGATCTACATC**TAGCGT**CTAAAG | -654 | 7.29 |
| 7 | CYB_1105 | - | AGTGAgaagccACACA | GCAGGCGGAAGGGTGCTCGT**GATTGT**GGGTG | -331 | 7.23 |
| 8 | CYB_2896 | - | GGTGAgcctgaCCTCA | ATTCTACCGCCTTGTCTTAGGA**TCCCGT**TCC | -154 | 7.16 |
| 9 | CYB_2126 CYB_2127 CYB_2128 | - - - | AGTGTatggctTCGCC | ATGCAAGCCATAGCCATTCCAAAC**TGGATT**G | -199 | 7.14 |
| 10 | CYB_2879 | - | GGTGAacactcTCCCC | GGCTATGCCTTCCAATGGG**TACGGT**CAGAGA | -405 | 7.08 |
| 11 | CYB_1664 CYB_1663 | rpsF - | TGTGAgcagccTCACC | TGCATTTGCCGCTGCGGCCA**GAGTAT**GGAGG | -128 | 7.03 |
| 12 | CYB_0517 | - | GGTGAtaccggTCACC | GCCCTGAAGCTCTTTTGGGTG**CAAGCT**GAGA | -72 | 7.03 |
| 13 | CYB_2207 CYB_2208 | - metH | CGTGAtcctaaTCACA | GACAACTAAAGGTGACTAAGG**GAGCTT**AGAC | -257 | 7.01 |
| 14 | CYB_2564 | - | GGTGAccggtgCCACG | GTTCCCCCGGCGATGCCTGACTC**TATTCT**GG | -68 | 6.96 |
| 15 | CYB_2795 | - | GGTGTcggagaACACA | TATCTAAGAGTTACCCTGGAGC**CATGCT**TAA | -128 | 6.91 |
| 16 | CYB_2030 | - | AGGGAacgagaTCACA | GCCACGGAAGACTTGAAGGGA**TAGCAT**AAAC | -179 | 6.91 |
| 17 | CYB_1412 | - | AGTGCtctggaAAACA | GACAGCCCTCGGTCAATATGC**TAGCTT**TAAG | -294 | 6.90 |
| 18 | CYB_2788 | - | GGTGAatcagaACACA | CCACAAAAGACAGCCAACGCCGT**CAAACT**GC | -79 | 6.90 |
| 19 | CYB_2828 CYB_2829 CYB_2830 CYB_2831 | - - - - | CGTGAtccaaaTCACC | TGCAAGGGGGGAAAGAGCGCGAGT**CAGGCT**A | -131 | 6.83 |
| 20 | CYB_2926 | - | TGTGAtctgtaTCTCT | GTATCCAATGAAAGTCTCTCAAGAA**GAATCT** | -314 | 6.81 |
| 21 | CYB_1106 CYB_1107 | recJ-2 - | TGTGTggcttcTCACT | GCTGCCAGTAGGGTTCAAGGTAGCC**CATCAT** | -117 | 6.80 |
| 22 | CYB_2824 CYB_2825 CYB_2826 CYB_2827 | psaF psaJ psaL-2 - | TGTGAgctaaaACCCG | GGATCCGTCGGGGCGAGGCGCTCAG**GAGTAT** | -123 | 6.80 |
| 23 | CYB_2597 CYB_2598 CYB_2599 CYB_2600 CYB_2601 CYB_2602 CYB_2603 CYB_2604 CYB_2605 CYB_2606 CYB_2607 CYB_2608 CYB_2609 CYB_2610 CYB_2611 CYB_2612 CYB_2613 CYB_2614 CYB_2615 CYB_2616 CYB_2617 CYB_2618 CYB_2619 | rplC rplD rplW rplB rpsS rplV rpsC rplP rpmC rpsQ rplN rplX rplE rpsH rplF rplR rpsE rplO secY adk map-2 infA - | AGTGAgctggcTCTCA | GTTGATGAAAACGAGTCGTGCAGGC**TCGATT** | -43 | 6.72 |
| 24 | CYB_2560 | - | AGTGAaaaggaTCTCA | AAAGAGGCAAAAAAGAGCATGAC**TCTAGT**CA | -304 | 6.71 |
| 25 | CYB_2019 CYB_2018 | - ureE | AGTGGttgtgcCCACG | CCCATTAGCAAGAGCTGACG**TAAGAT**ACTGT | -83 | 6.70 |
| 26 | CYB_2585 CYB_2584 | - - | TGTGGccttaaCCACT | TCCTTGAGGGGTTGTCGATTGT**TATGAT**AGA | -121 | 6.68 |
| 27 | CYB_0393 | codA | AGTGAgcaataACCCA | ATCAACTGGTTTCCTAAATTCG**GGCTTT**GAG | -126 | 6.68 |
| 28 | CYB_1386 | - | GGTGAtccggaTCAGC | GCAAGCCTTTGGTCAGGGGAG**TAGGTT**ATAG | -152 | 6.67 |
| 29 | CYB_1753 CYB_1754 | - - | GGTGAgggcatACACT | TCCTCCGGCGGCTCCCGGTCGGCA**TCTTCT**A | -594 | 6.65 |
| 30 | CYB_2118 CYB_2117 | - - | GGTGAcgagacTTTCA | CTGAGTTCACGCGGAGGT**GAGCTT**GGTGAGT | -104 | 6.63 |
| 31 | CYB_0008 | - | AGTGTgggatcCCACC | TCGCAAGAACCAGAGAAACGGC**TACAGT**AAC | -124 | 6.62 |
| 32 | CYB_1554 | - | CGTGGatcccaGCACT | GGCGAAATGGCGGGATCCTTTGT**TAGCAT**TC | -485 | 6.62 |
| 33 | CYB_0636 CYB_0637 | - - | GGGGAgcgggtTCACG | CCTTTTGAGTCTTGGCGATACGGCT**TAGCTT** | -44 | 6.61 |
| 34 | CYB_0622 | - | GGTGTccctctTCTCA | GCGATTCTCCAGAACTGT**ACCCAT**CGAGCCA | -113 | 6.60 |
| 35 | CYB_0415 CYB_0414 CYB_0413 CYB_0412 CYB_0411 CYB_0410 CYB_0409 CYB_0408 CYB_0407 CYB_0406 CYB_0405 CYB_0404 CYB_0403 | - dpsA - - nifE nifN nifX - - nifW - - - | AGTGAagactaTTGCG | GCGGCAGGCCGTCGGACTT**TCTTGT**TGTGAC | -282 | 6.59 |
| 36 | CYB_0967 | - | AGTGTgccgcaTCGCT | AGCCTAAGCCCTGTTGAGCGGGTT**TAATCT**A | -121 | 6.59 |
| 37 | CYB_0902 CYB_0901 CYB_0900 | - purN - | GGTGAcaatccACACA | GCCGCTCACCCGCACAGGAGTGG**GAAACT**CC | -670 | 6.57 |
| 38 | CYB_1365 CYB_1363 | - - | AGCGAtgttcgTCACC | GACAAATGGCGTAGGGTTGCT**TGGCTT**TTTG | -115 | 6.56 |
| 39 | CYB_0727 | - | TGTGTtgctctACACA | TTAGCATCCAACCCATCTGTACC**CAACGT**GT | -49 | 6.56 |
| 40 | CYB_0430 | - | GGTGAagaggcTGACC | CACGACTGGAGGAGCATCCCTCTC**TAAGCT**G | -145 | 6.54 |
| 41 | CYB_2269 CYB_2271 | - - | GGTGAtaaaaaTCTCT | CCCTTCTTAAGATTGAGTCGCAA**TAGCTG**GA | -83 | 6.52 |
| 42 | CYB_2562 | - | TGTGAatgcccTCCCT | CCCATCCGCTGGGATAGGGG**CAAGCT**ATGAA | -187 | 6.52 |
| 43 | CYB_2321 CYB_2322 | - - | TGTGTtccaaaATACA | AGCTTCTGGAAGATCTGCAGC**AAACCT**GGAT | -270 | 6.52 |
| 44 | CYB_0669 CYB_0668 CYB_0667 CYB_0666 | recQ - - - | TGTGAcagggaTCCCC | TACAGGCAAGCCGGTGGGGCCGA**TCCTCT**GC | -153 | 6.52 |

**Table S7**. Predicted CRP binding sites in *Anabaena sp* PCC7120 genome at *P* < 0.01

| Rank | Transcription Unit | Names | CRP TFBS | Downstream region of the CRP TFBS | CRP site position | Score |
| --- | --- | --- | --- | --- | --- | --- |
| 1 | all0853 all0852 | - - | TGTGAtttgagTCACA | ACAGTACTAATGCGGTTAATCACAA**TGAAGT** | -362 | 9.24 |
| 2 | alr0208 | - | GGTGTtgttttTCACC | TTAAATTAAAGTTTACTTAACCT**TAAAAT**TC | -195 | 9.23 |
| 3 | asr2365 alr2366 | - - | GGTGAtacaagTCACA | AGCTGATATTATCAAAATC**TCAAGT**ATCAAT | -98 | 9.23 |
| 4 | asr3217 asr3218 alr3219 | - - - | TGTGAtccaaaTCACA | GCAAATACTGACGAGGATCACT**AATAAT**TGC | -109 | 9.22 |
| 5 | alr0255 | - | GGTGGatgcgaTCGCC | CCAACAGCTAATTACCCTATGTG**TAAGCT**TT | -265 | 8.89 |
| 6 | all4297 all4296 all4295 | - - - | TGTGCtgttatTCACA | GAAAAAGTTTTGTGAATACTG**TCGAAT**ATAC | -100 | 8.81 |
| 7 | all4219 all4218 all4217 | - - - | GGTGTcatcacCCACC | CTTGCAAAAATAATGAGAC**TAACAT**TAACAC | -231 | 8.78 |
| 8 | all1542 all1541 | - - | AGTGTttttgtTCTCC | ACCCCCTGCATAGCACTTTCAAGC**TAGACT**A | -55 | 8.77 |
| 9 | asl4328 all4327 | - - | TGTGAtctaaaTCACT | AAAATAGACATGACAAGATCACA**TTTTAT**AC | -82 | 8.76 |
| 10 | asl2078 | - | TGTGTacgccaGCGCA | TAAATCCAACTATTAGCCCTCC**TAAAAT**GGG | -209 | 8.70 |
| 11 | all4822 | - | GGTGTaaatatACACA | AAAATTCCCTGACATTAGAAA**TTTACT**GCCT | -531 | 8.68 |
| 12 | alr4329 alr4330 | - - | AGTGAtttagaTCACA | CTTAAACTAAGTAGGTATTTCC**TGATTT**AAC | -377 | 8.65 |
| 13 | alr1044 alr1045 | - - | CGTGAcagaaaTCTCA | ACATAGCAAAATGTAAAGAA**CAACTT**AAGAA | -178 | 8.62 |
| 14 | alr3037 alr3038 | - - | GGTGAattttcTCTCT | GATTGCGATAATGTCCAC**TCTGCT**CGTTAAA | -83 | 8.58 |
| 15 | all4073 | - | TGTGAaccttgACACC | TGCTCAAAAGGCCACCAA**TAACCT**AATTCCT | -111 | 8.56 |
| 16 | all3837 all3836 all3835 | - - - | TGTGGaggcgaTCGCT | CACCCTTTTTACTTGAAGTTTCG**TGTTTT**GA | -139 | 8.53 |
| 17 | all4376 | - | AGTGCggtgctTCACA | CTAACAAAACATAGCAGAAATGTCC**TGCTTT** | -740 | 8.53 |
| 18 | alr3238 | - | AGGGTatgataTCACG | GTATAAATTTTAATTTTCAATAG**TAAAAT**AT | -205 | 8.51 |
| 19 | all1219 | - | GGTGAcatcatTCACA | ATCATGAAGAAATAAATGCC**TAAAAT**TATAT | -137 | 8.49 |
| 20 | asl1218 | - | TGTGAgtatttACACT | ATGAATTGTTAAAAAATAATTAAAA**AAATAT** | -329 | 8.49 |
| 21 | asl3262 asl3261 | - - | GGTGAaattttCCTCA | CTGATTCCACAGGATTTCCACAGG**GATTTT**C | -247 | 8.43 |
| 22 | alr4823 | - | TGTGTatatttACACC | TAGCATAGAATAATTTATTTGACGA**AAAACT** | -53 | 8.43 |
| 23 | alr4404 alr4405 | - - | AGTGCctgattACACC | AATACCCATCACAACCACA**AATCCT**TACCAG | -265 | 8.42 |
| 24 | asr0847 | psbN | TGTGCcatagtTCACC | TAAGATTAGTTGCCAAAGCTTTTTT**TAATCT** | -79 | 8.42 |
| 25 | asl0097 | - | AGTGGgagagaTCACA | TTTTGATAAATGTTATTGCCAT**TACCCA**GGA | -366 | 8.40 |
| 26 | alr3608 | - | GGTGAaattttTCACC | TTAATAAGGAAAATTTATG**TAATTT**CTTGCG | -150 | 8.37 |
| 27 | all0220 | - | GGTGTgagccaTCGCC | ATAAATATCTTGTAATTCCTGATA**AATACT**A | -243 | 8.36 |
| 28 | all2573 | - | AGTGCtttaaaACACA | TTCTGCGTAGCACATTAG**TATGTT**ATAACAC | -626 | 8.30 |
| 29 | alr0547 alr0548 alr0549 | - - - | AGTGGctagaaGCACC | TCGGAACGGGAATTAAGGAACT**TCCACT**A-- | -30 | 8.27 |
| 30 | alr1192 | - | AGTGAcaactgACACG | GGCAGCGCAAAGAAAATATTTGG**TATTTT**AT | -174 | 8.27 |
| 31 | alr3930 alr3931 | - - | CGTGAttgctaTCACT | GAAACCTTTATATAACGGGACTCT**TACTTT**T | -687 | 8.26 |
| 32 | asr0905 | - | GGCGGtgttcaCCACT | ACAATCTTGGGAAAGCCTATC**TAAACT**GGAA | -104 | 8.25 |
| 33 | asl1111 | - | CGAGAgtgtaaTCACC | TTTTCATTGGAAATTTCT**GATTTT**AGGAAAA | -624 | 8.25 |
| 34 | asr4524 | - | AGAGAaggacaTCTCT | CAGTCCTAGCCCGTCAGC**TAACTT**CGTAGGC | -457 | 8.24 |
| 35 | alr4525 alr4526 asr4527 alr4528 alr4529 alr4530 alr4531 alr4532 alr4533 alr4534 | - - - - - - - - - - | CGTGTaaagcaACACA | CCATTTGAGGTTTTATAGGTGTA**AATATT**AA | -418 | 8.22 |
| 36 | alr0444 alr0445 | - - | AGTGActtttgTCACT | TATGAAATCCAGCCTTCTTGGCT**GATAAT**TG | -109 | 8.18 |
| 37 | alr2968 | nifV2 | TGAGTgttttgTCGCA | GTACTGAAAAGTTTTGCTGGATG**TAGGTT**AG | -133 | 8.17 |
| 38 | all0443 | - | AGTGAcaaaagTCACT | AATATTTGCTTATTTATATATGAC**TATAGT**C | -479 | 8.16 |
| 39 | asl2353 | - | AGTGAattactACACG | GAAACCCCACTGAAAAAAT**TATAAT**CTTTAC | -122 | 8.14 |
| 40 | alr5293 | - | CGTGAttgtccTCACC | TAAATGGCATAAATAACT**GATTTT**GCCTATA | -524 | 8.13 |
| 41 | alr5001 | - | AGTGTaatttaTTACA | TATAAATCAATTGTAAAATTAT**TACACT**AAA | -182 | 8.11 |
| 42 | alr1146 | - | TGTGTtataaaACTCA | CAGAAATTTATCTTAGCGGAAAGC**TGCTCT**A | -32 | 8.11 |
| 43 | alr0029 | - | TGTGTataaatACCCT | TTCCCGAACCCAGTGACTG**TGTTAT**AAGAAG | -67 | 8.11 |
| 44 | alr1810 | - | AGTGGcaaataTTACC | TAGTTGTTGATAATGCGCTA**TATGCT**AATAA | -449 | 8.09 |
| 45 | asl0881 | - | CCTGAtaaaatTCACA | ATTTATGATGTTAGTTAT**TATTTT**TAATCTA | -680 | 8.08 |
| 46 | all5024 | - | GGGGAactcaaACACA | AGTAAGATGTAGAGGTTTTACCTC**TCGATT**A | -74 | 8.08 |
| 47 | alr3565 | - | AGCGAtcgcatTCACC | CAATCGGGTGAACCATCGATTC**TTCGGT**CAA | -288 | 8.08 |
| 48 | all0349 | - | TGTGTtttcaaTTACT | GCATGGGTTATTTTTTCAGATA**TAAAAT**ATG | -298 | 8.08 |
| 49 | all2415 | - | TGTGAttaactCCACT | GACGAAATAACAGCATTTAG**TAAAAT**TTAGA | -301 | 8.07 |
| 50 | alr5205 | - | GATGAtcctgaTCACA | TACTGAATTAGTTAATACCAGC**TATACT**TAA | -77 | 8.06 |
| 51 | alr4168 | - | AGTGGgagatgCCACA | TTTTAAAAATGTTATGTCAGTATT**TAAAAT**A | -530 | 8.05 |
| 52 | all1191 | - | CGTGTcagttgTCACT | TGCTAGTTGTTTTATATC**TAGATG**AATACCT | -768 | 8.05 |
| 53 | alr0030 | - | GGTGTgcattaCCACC | AATCAAAATCTATAAAGA**TAGGTG**GTGAAAA | -33 | 8.05 |
| 54 | alr2920 | - | AGTGGtttaagGCGCA | GACCTGGAAAGTCTGTAATGCGG**AAACGT**AT | -270 | 8.04 |
| 55 | alr2510 | - | TGTGTaagcccTCACT | GTATTTCTGTTGTCCCTC**TAAGCT**ATTACAC | -520 | 8.04 |
| 56 | asr5289 alr5290 | - - | TGTGTcctaacTAACT | CTATAATTTTCTAGGTCTAGAT**TAACTT**TGC | -150 | 8.04 |
| 57 | alr0276 | - | AGTGCctatttACACG | GACAAAATTACTAAAAAT**TAATTT**TTCGTAG | -134 | 8.02 |
| 58 | all3327 all3326 all3325 all3324 all3323 | - - - - - | GGTGAttttcaCCTCA | ATGGAAAAGCCTAATATG**TATCAT**TGTCTAT | -268 | 8.02 |
| 59 | all1511 asl1510 all1509 all1508 | - - - - | AGTGGgtggatTCTCA | TCCATTTCTAGTAAATCCGTAGG**TAAAGT**CA | -150 | 8.02 |
| 60 | all2371 asl2370 | - - | AGTGGaatattACCCA | TTTTTGCAGCATGACTTGG**TAAACT**ACACAT | -358 | 8.01 |
| 61 | asl3784 | - | TGTGAattaatTCTCT | CGAAAGATTAATTTTTCTCAAAA**TACTGT**AA | -502 | 7.98 |
| 62 | all3310 | - | CGTGTcttttgACACA | ATTTTACAAAAAAAGTAACAA**TACCAC**TTAG | -59 | 7.97 |
| 63 | asr1203 | - | TGAGCatttctACACC | ATCTCCTATTTTCTGCTAAA**TATAAT**TAAGC | -278 | 7.97 |
| 64 | all0187 | - | AGTGCggtcttACACT | GGCAAGATTGGTAGCTTGCTT**TAACAT**AAAT | -61 | 7.96 |
| 65 | asl0639 | - | CGGGAtgggttGCACT | ATCGCCCTACCCATCTCACTATA**AATGTT**GG | -261 | 7.93 |
| 66 | alr2131 alr2132 | - - | CGTGTcgccttTCCCC | GAACAGCACCCATAACAATTCAAAA**TACCCT** | -356 | 7.93 |
| 67 | alr4627 | ilvN | GGTGAcaatatTCTCA | AGATAAGATTTTGATAAA**TAATGT**ACATCTG | -214 | 7.92 |
| 68 | alr1652 | - | AGTGCttaaaaTCACA | TCCAATCACTTTTCAAACATCCTC**TTAGAT**C | -133 | 7.91 |
| 69 | alr2071 | - | TGTGCatttcgGCTCA | CACTTGTTTTATACAGAGAAGCAGT**TATACT** | -124 | 7.91 |
| 70 | alr2834 | - | GGTGCgatcgcACACA | TCTTTTTATTCAGCAAATAATTC**TCAATT**TT | -699 | 7.89 |
| 71 | all2509 | - | AGTGAgggcttACACA | TGAAAACATACCCATTAGGGGTA**TAGGGT**AA | -238 | 7.89 |
| 72 | all3135 | exoV | GGTGTtcaaccGGACA | CGATATAATCTCTAGTCTC**TATGCT**CATGAA | -50 | 7.88 |
| 73 | all4875 all4874 all4873 all4872 all4871 | - - - - - | AGTGAtcctcaACCCA | GGCTATCTTCTGAAGGTAGAGTGGC**AAATCT** | -148 | 7.87 |
| 74 | all1711 | - | AGTGGtcaagtTCACA | TTAGGGATTTATTAGATAAACCAAA**TTGATT** | -210 | 7.87 |
| 75 | all2919 all2918 | - - | TGCGCcttaaaCCACT | CGGCCATCTCTCCAGATGTCACGA**TTTACT**A | -119 | 7.86 |
| 76 | all3207 all3206 | - - | AGTGAatttccTCATT | TCTTTTGCTGAGTTATTATGTAACA**TAATAT** | -188 | 7.86 |
| 77 | alr1877 alr1878 alr1879 | - - - | AGCGTatctatTCGCT | GAAGCTCCAACTTATTCC**TAGCAT**TTTTGGG | -130 | 7.86 |
| 78 | all4465 all4464 all4463 all4462 all4461 | - - - - - | TGTGCagcagtACACT | CTCCTGAGAACGTATATCTTT**TAAAGT**AGTA | -134 | 7.85 |
| 79 | alr3069 alr3070 alr3071 | - - - | CGTGAttcaagACGCG | GACAGAAATTACTAATGA**TATCAT**CATCCAT | -128 | 7.85 |
| 80 | alr1081 | - | AGCGAaaataaTCACC | GCCGATACACACCAATGAACGCTGA**TAAATG** | -318 | 7.85 |
| 81 | all0059 | - | AGCGAtcgcctACACA | TACACTATCCCTAAAAGCTATACA**AATATT**C | -471 | 7.85 |
| 82 | alr0428 alr0429 | - - | AGTGGaaacccACGCA | TCATAGTGGACTCCTGTTTT**TAGGTT**AAAAG | -149 | 7.85 |
| 83 | alr3758 | - | GGGGAtattatCCACA | AAAATACTTAATCTCATCCAATAAT**TAAATT** | -273 | 7.85 |
| 84 | alr3201 alr3202 | - - | TGTGAaagcgaTCGCT | CTTGGCGGTTCCTATGAATGA**TAATTT**TAAG | -61 | 7.84 |
| 85 | alr0317 alr0318 | ccmK - | GGTGTctaaagCCACG | TACAATTAATATCACTTAACT**TTTTTT**ATCA | -45 | 7.82 |
| 86 | all2134 all2133 | - - | TGTGGgttcagTCTCC | TACTGGCGGTGGGGTGGTT**TAACCT**CGCCAG | -522 | 7.81 |
| 87 | all3564 | - | GGTGAatgcgaTCGCT | CCAGCAGGAATTAACCTGAAG**TAAGAA**GCAG | -82 | 7.81 |
| 88 | alr3311 alr3312 | - - | TGTGTcaaaagACACG | CTGTCGTATTTCCCCTAG**TTTATT**GCGAATG | -575 | 7.81 |
| 89 | all0127 all0126 | - cysA | GGTGAtttctcGCTCT | GCCTCCAGGTTTTGACAGAAATGC**AATATT**C | -197 | 7.80 |
| 90 | all4668 | - | GGTGAgtgcagCCACA | CGTGCGGCTATGCCGCCGGG**TGGACT**GCCGA | -345 | 7.80 |
| 91 | alr3248 asr3249 asr3250 alr3251 alr3252 | - - - - - | TGTGTtatgaaATACA | GATTTTATACTTAGCTTTG**TAGCAT**TTGCCC | -118 | 7.79 |
| 92 | asl3322 all3321 all3320 all3319 all3318 | - - - - - | AGTGAtgagaaTAACT | AACGACAACTGACAAGAGGA**TAAATT**----- | -27 | 7.79 |
| 93 | alr1263 | uvsE | TGTGActgttgACAAA | AATGCTGCTGTGTGTACGT**TATTTT**AGGTGA | -507 | 7.78 |
| 94 | alr3714 alr3715 | - - | AGTGTtaggatGCCCA | AATTTATGCTTGTACATTGCTG**TATTAT**GGG | -62 | 7.78 |
| 95 | alr2574 | - | TGTGTtttaaaGCACT | CTGTGTGAATAAGGGCTGAATA**AATATT**TAT | -135 | 7.78 |
| 96 | alr3057 alr3058 alr3059 alr3060 alr3061 alr3062 alr3063 alr3064 alr3065 alr3066 alr3067 | - - - - - - - - - - - | AGTGTatatacTCTCT | ACAATGTAAAGTTTTTGCAAAGAG**GGGAAT**G | -182 | 7.77 |
| 97 | all2962 all2961 all2960 | - - - | AGTGAgtgcaaACACC | TTTATAGGGAGAGTCTCTGA**TGAATT**ATTCA | -238 | 7.76 |
| 98 | alr3932 | - | ATTGGtcgcaaTCACA | TAATCTTGATAAGAAACTAC**TATGCT**GACGG | -131 | 7.76 |
| 99 | alr0113 | - | GGTGTgaatcaACACT | CTCGATAGTGGACACCAATTTAGT**TATACT**A | -49 | 7.76 |
| 100 | alr2210 alr2211 alr2212 alr2213 alr2214 alr2215 | - - - - - - | TGGGAttcctcGCACT | GATGCACCCTATATTTGTAA**TATTTT**TCTCT | -137 | 7.75 |
| 101 | all2456 | - | AGTGGattactTCCCC | ATATTCTTATAGTCTGACA**TATTTT**TCTGTT | -136 | 7.75 |
| 102 | all0275 | - | CGTGTaaatagGCACT | GGAACTTTACTGAAGTCAGATTAA**GAGAAT**A | -237 | 7.74 |
| 103 | alr3965 | - | AGCGTgcgtagTCGCA | AGCATCCTTCACCGTATT**TTTTCT**CACTGCT | -41 | 7.74 |
| 104 | all0435 all0434 | - - | TGTGGtgttaaAGACA | ACTTATTTTAACTACAACTTTATG**TATGCT**A | -208 | 7.72 |
| 105 | all3652 all3651 all3650 | - - - | TGTGAttgttaTCACA | TTATTTTTCCGGATAACCTG**TATCTA**CGTGA | -321 | 7.72 |
| 106 | all0498 all0497 | - - | GGCGAtcgctaGCACC | GCTAAGAATCGGTATCTAAGCAACG**TAAGAT** | -167 | 7.71 |
| 107 | alr1665 | - | GGTGGcttttcTCCCT | ACCAATCATGAGTTAGGCTTTCCC**TCTACT**T | -86 | 7.71 |
| 108 | all0458 | - | TGGGAgcaattTCACG | AATCAGCCAATAAGTCTTGTAAAAA**TACTCT** | -249 | 7.71 |
| 109 | alr2749 | - | TGTGGcctcaaTCACG | TAACTTAGAGTTATGCTAATT**TTTATT**AAGA | -56 | 7.70 |
| 110 | alr4711 alr4712 | - - | AGTGAccaataTGACC | TAATGTTTTGCCCTAGTAT**TCTGTT**GAGCAA | -125 | 7.70 |
| 111 | all1682 | - | TGGGAactgctTGACA | CAAATTATCACAGTAGTTAAATT**TACATT**AA | -236 | 7.70 |
| 112 | alr3768 | orrA | GGTGGatgcagGCGCA | TCAAGATATCATCTATCTT**TGGTTT**GGGAAA | -100 | 7.70 |
| 113 | all2894 all2893 | ruvB - | TGTGTattttcTCACT | AAAATCTTCGCCGCCATT**TCTACT**ACCAGTA | -85 | 7.69 |
| 114 | asr0081 | - | GGTGAgtgcagCCACA | CGTTCGACTATGCCACCGAG**TGGACT**GCCTG | -527 | 7.69 |
| 115 | alr3268 alr3269 | - - | CGTGAaaagttCCACA | AGAAAATATTTAGACTCGACACTA**TTTGTT**A | -213 | 7.69 |
| 116 | all2059 all2058 | - - | GGGGGagaataACACA | ACTAACCACTGACAACCGACTAA**TGAATT**TT | -81 | 7.69 |
| 117 | alr1580 | - | AGAGAaaatttACACA | CCGCTTTAAGTTCACTCT**CATGGT**TAGCAAT | -309 | 7.69 |
| 118 | alr1057 | - | TGTGAgagtcaTCACT | CATCACTTGATTAAAAACC**TACACC**TGTGAG | -309 | 7.68 |
| 119 | alr0169 | - | TGTCAtcgattACACA | CTTTTCAGTCATGCACCAGA**TAGAAT**CATGA | -310 | 7.67 |
| 120 | alr2137 alr2138 | - - | GGTGAtcgctaACACT | AAGCTGTACGCTAACGCACA**TTTATT**CCTAA | -678 | 7.67 |
| 121 | alr1341 | - | AGTGGatagagCCACG | GATTTCTAATCCGTTGGTCG**CAGGTT**CGAAC | -236 | 7.66 |
| 122 | all5295 | - | TGTGAgcttttGCTCA | TATACTGCGAAACATGATGTGAG**TAATAA**CT | -89 | 7.66 |
| 123 | asr0798 alr0799 | - - | GGTGAtgggaaACACG | AAGGAAGCATGGTCATCAGGAG**TAAAAT**TAA | -166 | 7.66 |
| 124 | alr0819 | - | GCTGTaacttaTCACA | AACATCAGTGATTGCTAG**TAAATT**AATTGTG | -194 | 7.66 |
| 125 | all4719 | - | TGAGAtttttcACTCA | TACGATCGCCTGGTAACA**TTATTT**GCGACTA | -200 | 7.65 |
| 126 | all4406 | - | AGGGAgaggatTCACA | GGTAAGGAACTCGGACTATCAACA**TTAATT**C | -87 | 7.64 |
| 127 | alr2118 alr2119 asr2120 | - - - | AGTAAatttccTCACA | AGTTCCTCATCTTTCTCGTTGAGAA**TAATTT** | -59 | 7.64 |
| 128 | all0207 | - | GGTGAaaaacaACACC | TAATTATCTTTAATTAATA**TATAAA**AGGAGT | -140 | 7.64 |
| 129 | all0687 | hupL | GGTGGctaaagACACA | GCCCCCAAATGGGCAGAAGAA**GACTTT**TTCA | -107 | 7.63 |
| 130 | all3227 all3226 | - - | CGTGAgatagtCCCCC | TTATACATATACTTATGCGAA**AATACT**AAAA | -44 | 7.63 |
| 131 | alr3599 | - | GGTGAagctttTCTCT | GTTTTTCAGTCCCCAGAACTCTGA**TTTTCT**A | -46 | 7.63 |
| 132 | all4659 | - | TGTGGgcatagCCGCG | CTTTAAGCGCAAGGCATT**TAGTCT**GTGCTGA | -321 | 7.63 |
| 133 | all4052 all4051 | - - | AGTGTaggcgaTCGCC | TTAACTTAATAGTTAAGA**TCTGTT**TGAAAAA | -526 | 7.63 |
| 134 | alr4812 | patN | TGTGGacaattTAACA | TTCATGGTCTTGAATATTATCATCA**GAATAT** | -172 | 7.63 |
| 135 | asr5004 alr5005 | - - | GGTGTggttacGCACA | ACGCGGTAGTATTGGGGAATGTA**TGCGAT**AC | -416 | 7.63 |
| 136 | asl0046 | - | TGTGCcagtttTTACT | TGTTTTTATTAGTGAAAGAT**TACCTT**TCTGA | -175 | 7.62 |
| 137 | all4403 | - | GGTGTaatcagGCACT | AATATTGTGTTAATAAATG**TATGGC**TAGTAG | -78 | 7.62 |
| 138 | all0233 all0232 | metG - | TGTGTtcttcaTCACC | ATAAATGTAATGAAAGCATTCTCC**AAAATT**A | -223 | 7.62 |
| 139 | alr2306 alr2307 alr2308 alr2309 alr2310 | - - - - - | TGTGTgtgaggCCACA | CGCTTCATTTGTTCTTCTGA**AACAGT**TACTC | -122 | 7.62 |
| 140 | all1119 all1118 | - cyaA | TGTGAttttaaGCACT | TATTGATCCCCCTAACCCCCC**TTTTTT**AAGG | -184 | 7.62 |
| 141 | all0638 all0637 | - - | TGTGAttttaaGCACT | TATTAGTACCCATCAAGCCCCC**TAAACC**CCT | -464 | 7.62 |
| 142 | all5177 all5176 | - - | CGTGTttatctAGACT | CTGCATCAAGGGATAGAGGCTTGG**TAGGAT**C | -75 | 7.61 |
| 143 | all2677 all2676 all2675 | - - - | TGTGGactcaaTCACC | AATACCTGATTGATCAAGAT**TTCACT**GAAGG | -306 | 7.60 |
| 144 | asr0098 alr0099 asr0100 alr0101 | - - - - | TGTGAtctctcCCACT | AAAGTTGGGAATAATAAAATCAA**CCACAT**CC | -116 | 7.59 |
| 145 | all4085 all4084 | - - | AGTTAaaaattTCACA | AATGTCTTAAATCTCTACG**TACATT**TATTGA | -68 | 7.57 |
| 146 | all0037 | - | AGTGTtagtgtTCGCT | ATACAAAAATCCTGGGAACAC**TAAATT**ATAT | -120 | 7.57 |
| 147 | all5222 | - | AGTGCaacttaACACA | GAAAATTTGTGCAGTCCGTGA**TTTAAT**GATT | -135 | 7.56 |
| 148 | all0385 | - | GGTGGagggtaGCCCA | TCGCTGCTAGTTTAATAATGAAT**TACTCT**AC | -39 | 7.56 |
| 149 | all1651 asl1650 | - - | AGAGGactcacGCACT | TTATAAATAGACAGCCTACAG**TAAAGT**ACCG | -537 | 7.56 |
| 150 | all2989 | - | TGTGAacttcaTTACA | TCTTCAAGTTGGGAAATCCACCCT**TACCTT**A | -187 | 7.55 |
| 151 | all3255 | - | AGAGTcatcaaCCTCC | CACATAACCTTCGGCGAAGTCA**TAGGCT**ACT | -58 | 7.55 |
| 152 | all0011 all0010 asl0009 all0008 all0007 all0006 all0005 all0004 | atp1 atpI atpH atpG atpF atpD atpA atpC | TGTGAttaattACACA | ACAAGGTTGAGGAGCATTCCC**TGATCT**TGCC | -284 | 7.55 |
| 153 | all3964 all3963 all3962 | - - - | TGCGActacgcACGCT | TTGCGGGAAGCTTCCTATAGTGT**TCAAAT**AG | -50 | 7.54 |
| 154 | alr1941 alr1942 | - - | TGTGCtttttgTACCT | GACGTTAGACAATAATGCTACAAG**TAGTGT**T | -56 | 7.54 |
| 155 | all2964 | - | TGTGTtttttcTAACA | ACTTATTTATAATTGCTA**TATTAT**CAATTTA | -84 | 7.54 |
| 156 | alr1285 alr1286 | - - | TGTGAgctaatATACA | TAGAATCGTGTTTGCATTTTTTGC**AAAACT**A | -58 | 7.54 |
| 157 | alr0348 | ndhD | GGTGGttggagCCACC | GTTTTTTGTTGACGTTACAA**AAATTT**ATGCC | -220 | 7.53 |
| 158 | alr1555 alr1556 | - - | AGTGGtagagcACACC | CTTGGTAAGGGTGAGGTCACGAG**TTCAAT**CC | -720 | 7.53 |
| 159 | all3624 | - | TGTGAatgctgCCACA | ATAATAGCCAACTCTACCAATTAC**TAAAGT**A | -151 | 7.53 |
| 160 | all0323 | - | AGAGAttaattTCTCA | AAACTCATTGAGGATAAGCTAT**TACGCT**TTT | -175 | 7.52 |
| 161 | all3866 | - | GGTGTtgacatACACC | TTAGCTTAGTTGTAGGGGTGT**AAGGGT**GTAA | -89 | 7.52 |
| 162 | asr5139 alr5140 alr5141 asr5142 alr5143 | - - - - - | CGTGTtttccaACGCG | AATTGTAGTTTGAGTTTC**TTTCCT**GAAAGCA | -443 | 7.51 |
| 163 | all1601 | - | AGTGCcggcttCCGCT | ATCTTAAGTTTTGAGATAA**TAAATT**GCGCTT | -34 | 7.51 |
| 164 | all4756 | - | GGTGAgcaattTCTCG | GTGCAGCTAAGTTTTCTTGCAAG**TAGCGC**AC | -254 | 7.51 |
| 165 | alr0739 alr0740 | - - | CGGGTcaaaagACACT | ACTCTGCAAAAGTAGCCCATT**TAACTT**TAGA | -373 | 7.50 |
| 166 | all2165 all2164 all2163 all2162 | - - - - | GATGAgaccttCCACA | AGTCGTATTCCTCTATGTAT**TAGAGT**TAATG | -231 | 7.50 |
| 167 | all1221 | btpA | AGTGGcaaactCCTCT | TGTAGCCAGGTTTTATCTTAACA**TATCTT**AT | -258 | 7.50 |
| 168 | all3335 all3334 all3333 all3332 | nrtB nrtC nrtA - | TGAGAtgatggACGCA | AAAATTATACTCTTAATAGTTTCAA**AAACTT** | -77 | 7.50 |
| 169 | alr2426 asr2427 | - - | TGTGAtaaataGTACA | TTAGTAGCTAAAACACATAA**TTATTT**CCTGA | -83 | 7.49 |
| 170 | all3967 asl3966 | aat - | AGTGCagaggaGCGCC | AAGCATGAGTAACAACATCAGAG**CGCGTT**CC | -60 | 7.49 |
| 171 | all0316 all0315 all0314 all0313 | - - - - | CGTGGctttagACACC | ATTGTGATGATGAGGGGGAAATT**TCTTTT**GT | -93 | 7.49 |
| 172 | alr3855 | - | TGTGTgaatcgTCACT | GCGCCACTTTAGTATTAAAGGTAT**TGAGAT**T | -88 | 7.49 |
| 173 | alr3235 | trpC | AGGGAataatgGCCCC | CACACCCCTATACCCTTA**TACCCT**TTCTTAA | -62 | 7.48 |
| 174 | alr2335 alr2336 | - - | TGCGTagcccaTCGCA | CAGAAGATTGTCTGTATTT**TAACCT**CACTAT | -193 | 7.48 |
| 175 | asl4022 | rbpD | TGTAAaatctcTCACT | TTGAAAATTATCATGATCAGT**TATTAT**ATGA | -203 | 7.48 |
| 176 | all2883 | - | TGCGTaatactGCACT | AGTAGCTAGTTTTAATGGATGATC**TAAATT**T | -671 | 7.48 |
| 177 | alr3265 asr3266 | - - | GGCGAtttttaACCCT | ATCCTGAGATAGAGCTTTT**TTTGTT**GCCCAT | -111 | 7.48 |
| 178 | all4035 | - | GGTGTgtgattTCACC | TTATTTTAGATTGATATATA**TTGCTT**ATTAC | -288 | 7.47 |
| 179 | asl1243 | - | GGTGTaaaaaaTGACG | ACACTACTGTTAAAACAGCATCAAA**CAGCTT** | -100 | 7.47 |
| 180 | asl4253 | - | GGTGAggcgtaAGCCC | TTGCTGAGTAAAAATTCTAGCCTC**TAGCCT**C | -124 | 7.47 |
| 181 | asl0260 all0259 | - psbV | GGCGAcaaattGCACT | CCTAACATGATATTGGTAT**TACAGC**GACTTA | -89 | 7.47 |
| 182 | asr4669 | - | TGTGGctgcacTCACC | GCCCACGCAACTGGCTCCCCTA**TACCCT**TGT | -550 | 7.47 |
| 183 | alr1600 | - | TGTGCcaacctTCACC | AAACACTAGGACAGCTACCCACC**AACAGT**TA | -703 | 7.47 |
| 184 | alr4977 | recR | TGTGAaatctgCCGCA | ATCCTAACCGTGAAAATAA**TACTAT**CTGCGT | -52 | 7.46 |
| 185 | alr4852 alr4853 alr4854 | - - - | AGCGCtgaggtTCACG | CAGCGAAAAGTTCTGTTCGCG**TAGCGT**CTCC | -132 | 7.46 |
| 186 | all3854 | psbO | AGTGAcgattcACACA | GACTCATAGGAAGCCTTA**TGGGAT**CAACACT | -309 | 7.46 |
| 187 | all2013 all2012 all2011 | - - - | TGCGTttaaaaACACA | ATGATTTTAGATAAAGCTGTAC**TAAATT**AGC | -85 | 7.46 |
| 188 | asr0890 | - | CGTGCtattacACTCC | ATTAAAACTATGATGTAGGCA**TAAATC**GCCC | -233 | 7.45 |
| 189 | alr2895 asr2896 | hisF - | AGTGAgaaaatACACA | GGTCAATTTAAAATTTAAATTCCG**GACAAT**T | -55 | 7.45 |
| 190 | alr5186 asr5187 alr5188 alr5189 alr5190 | - - - - - | GGTGTaacctcTCTCA | GCAATGGGAATTATTCTATTGTAAG**CAAGCT** | -167 | 7.45 |
| 191 | all5037 all5036 | - - | TGTGTaatttaTCACA | CAGTCACTGGAAGTAATGG**TATTCC**GCCCAG | -85 | 7.45 |
| 192 | alr1397 alr1398 | - - | GGTGTcgataaTTACA | ACTAAGCGGTTATTATGAATG**TATTAT**ATGC | -180 | 7.45 |
| 193 | asr1234 | - | AGTGTtattaaGCTCT | TTTTTTAGATTTAAAGCCTGATT**TGTGTT**GT | -361 | 7.44 |
| 194 | alr2856 | - | TGTGAcaacacACACG | GCTTTTATTTGATCTGCTAGA**TAAATA**GATA | -638 | 7.44 |
| 195 | alr1752 | - | TGTAAttaaagTCACA | TTTGTTTTATTTTACTTCATCAG**TGTTAT**CG | -122 | 7.44 |
| 196 | all3767 all3766 all3765 all3764 | - - - - | TGCGCctgcatCCACC | GATAGAAATAATAAATCTATT**TTTTTT**GGGT | -461 | 7.44 |
| 197 | asr1135 alr1136 | - - | AGTGTtgatgcTCACG | ACTGCTTGCGGTGGTGGAAACG**TAGCTC**AAC | -197 | 7.44 |
| 198 | all2395 | - | AGTGCtgatttTCTCG | TCATCAATTCCCCATCACCTTGCT**CATTAT**T | -215 | 7.44 |
| 199 | alr3698 | hepB | ATTGAagaaaaTCACA | TTTAGGATGTAGGAAGCCCTAGTAT**TATCTT** | -666 | 7.43 |
| 200 | all3292 all3291 all3290 all3289 all3288 | - - - - - | TGCGAcacaaaTTACA | CCAATAGGTTCTCTATCTCAGGCA**TACTTT**A | -86 | 7.43 |
| 201 | all0432 | - | AGTGTtgtttgACTCA | AGTAAATTTATGTACCTG**TAAGTT**CTGAACA | -117 | 7.43 |
| 202 | alr3285 | - | TTTGAacaaacTCACT | CATCATCTGTTCGCATTCATC**TAAATT**TAAA | -396 | 7.42 |
| 203 | all4333 | - | AGTGGcatttaGCCCA | TCAACTACTTTGATTGAA**TAGTTT**TTTCCAT | -98 | 7.42 |
| 204 | alr3330 alr3331 | - - | AGTGTtttttaACACG | AACAAGATTTGATAAATAG**TTTAAT**CAACGT | -437 | 7.42 |
| 205 | alr2903 | - | TGTGGctgcacTCACC | GCCCACGAGACTGGATCAGG**AATTGT**GGGTG | -494 | 7.42 |
| 206 | alr3140 | - | TGTGAcaatttTGTCA | CAATTTAGGGAAATTTAGAC**AAAGTT**TTTCA | -203 | 7.42 |
| 207 | all3267 | - | TGTGGaactttTCACG | AATGGAGATTGCTATTTTTTCTACA**TAACTG** | -201 | 7.42 |
| 208 | alr1786 | - | TGTGAccaaatGCTCA | CTAACGCGAGATAATAATA**AATCCT**CACCAC | -119 | 7.42 |
| 209 | all0985 | - | GGTGTcacataTCTCA | GGAGTTGAGTCCCTTCTTCCTGCCT**TCTTTT** | -126 | 7.41 |
| 210 | alr0264 | - | CGAGTtatcggTCTCA | ACGAAAAGTATAAACAGGCAACAG**CAAAAT**A | -137 | 7.41 |
| 211 | all2128 all2127 | - - | AGTGAagggtgAAACC | TGAAAACAAAAATCTCAGTTT**TTCAGT**AGTT | -83 | 7.41 |
| 212 | alr1109 | - | TGTGGtttgatCCCCC | TAAATCCCCTTAAAAAAGAGGAC**TTTGAT**TC | -568 | 7.40 |
| 213 | all4460 | - | TTTGActagtaTCACA | ACTCACTCATGATGTTAATA**CACGAT**GCAGA | -141 | 7.40 |
| 214 | all4026 all4025 all4024 all4023 | - - - - | GGTGTttatagTCCCA | GATAGAGATGAGGATATTCTTTGA**CAGATT**G | -118 | 7.40 |
| 215 | all2567 all2566 | - gap1 | TGTGCcaccgaCCACT | GAATCCTCACAACTAACA**AAAACT**CTATCCC | -400 | 7.40 |
| 216 | asr2781 alr2782 alr2783 alr2784 | - - - - | GGTGCaaaattACGCC | AGTTTCTGCGTAGCTGTTAGTC**AAAATT**GAG | -59 | 7.40 |
| 217 | alr1656 | - | GGTGAtttaaaTCCCA | ATTTGATATTACGTGTAGACG**TAGCGC**AAAG | -180 | 7.40 |
| 218 | all3892 | - | GGAGAcagaaaTCACC | AAAACATTTGAGCATGATGC**TTTTTT**AGAAG | -240 | 7.40 |
| 219 | alr0234 | - | GGTGAtgaagaACACA | AATCTCCCAAACTAGAGAATTTTGC**GGCTTT** | -142 | 7.39 |
| 220 | asl4039 all4038 all4037 | - - - | TGTGAaacataCCTCT | TTTCTTACTAGTTTTCAC**TTTTGT**G------ | -26 | 7.39 |
| 221 | all3459 all3458 | - - | TGTGGctatttTCTCA | GCTATTTAGCTAAGATTGTAGC**GAAGTT**ACT | -568 | 7.39 |
| 222 | alr3752 | - | AGCGAtgatatCCGCT | AAAAGAGCGTCGCTGTAACAT**TTCTAT**CACA | -249 | 7.39 |
| 223 | all2260 | - | CGAGAttctagGCGCT | CAATCGCTTTATTTTCATTAAAAG**TAGGTT**C | -329 | 7.39 |
| 224 | alr1112 | - | GGTGAttacacTCTCG | GCAACTAATGCTAGAGTTTCAG**GAAATT**CAT | -55 | 7.39 |
| 225 | alr0092 alr0093 alr0094 | - - - | TGTGAttaggtCAACT | GCCAAAGAATGATAATATTTT**TCTCTT**ACAC | -415 | 7.38 |
| 226 | alr2234 | phoD | TGTGTtagctgTCGCA | ATGCTACAAACTATTTGT**TAGTCA**GGAGTAG | -36 | 7.38 |
| 227 | alr1926 alr1927 alr1928 | - - - | AGTGGtgtaaaACACT | AAATTAAATCACTGTCACTGTTAA**GAGAAT**T | -718 | 7.38 |
| 228 | asl5079 | - | GGCGAtcgccaTCACA | GCATAAATTTAGCTGCAACTG**TGATTT**AGTA | -79 | 7.38 |
| 229 | alr4123 | prk | AGCGAaaatctTCCCA | AATTCGCTGCAAAAAAAATTTA**TACCTT**ATA | -412 | 7.38 |
| 230 | all4989 all4988 | - - | AGCGTcaaggtTCGCT | GATGAAACTGCATCAACGTATC**AACAGT**GAA | -115 | 7.37 |
| 231 | all2910 all2909 | - - | TGTGCaaaagtACTCT | TGAAACTTTGTTAAGTTTGG**TATCTT**TCTGA | -60 | 7.37 |
| 232 | all2528 | - | TGGGAtgctccACACC | ACTTGCTTATTGAGAAGATG**CAAGAT**AGCAG | -352 | 7.37 |
| 233 | all3850 asl3849 | - psaI | TGTGCttatcaACACG | ATAAGACGCGGACACGGA**TGACAT**CTGCCTC | -256 | 7.37 |
| 234 | alr1548 | - | TGTGAcagattCCCCA | AAAGCCATAATTACGGTAATTTTGG**GAACTT** | -161 | 7.37 |
| 235 | alr0599 | - | AGTGCttaattACCCA | TCCTTAGTGAACACTTCT**TAGACT**GCTACTT | -117 | 7.36 |
| 236 | all0441 | - | TGTGTtatgaaTCCCA | TTTCATCCGTTATGCTGTG**TTTGCT**GCCGTG | -78 | 7.36 |
| 237 | all1574 all1573 | - - | CGTGAatagcaTAACG | ATCAGAAAGCAGGTTAGCAATT**TAACAT**AGT | -300 | 7.36 |
| 238 | alr0566 | - | GGTGAcaaaagTCAAA | AAATCATGAGTTTCATCAAAG**AATTTT**GCAA | -342 | 7.36 |
| 239 | alr3993 | ribH | TGTGTtctcctGCTCT | TCAATATTTAGAGAAGAAATG**TATATT**GACA | -61 | 7.36 |
| 240 | alr3737 alr3738 | - - | TGTGTtaccgtTGGCG | GGGCGATGAGCAGCGTCC**TAAATT**ATGAGCT | -75 | 7.35 |
| 241 | all2364 | accD | TGTGActtgtaTCACC | CTCTTAACGGAATAGGACT**TAGGCA**ACGAAA | -539 | 7.35 |
| 242 | asl1777 all1776 | - - | GGTGTaaggctTCCCT | ACCCCTAACTTGAAAAAGCATGG**GAGGCT**GG | -68 | 7.35 |
| 243 | alr4466 | - | AGTGTactgctGCACA | TTTATATATTCAAACTTTATTAATA**AAAAAT** | -460 | 7.35 |
| 244 | alr0730 alr0731 | - - | TATGAccagccTCACA | AGTTCCTCATGTTATAGCTA**TAAATT**TTTGC | -75 | 7.35 |
| 245 | all3237 asl3236 | - - | GGTGAagatttTCAAT | GTTGTGCAAAATGAAAAGAACA**TTGCCT**ATA | -632 | 7.35 |
| 246 | alr1196 alr1197 alr1198 alr1199 | - - - - | AGTGActacttCCGCT | ACTAGCTATAATTTCATTT**TGATTT**ATCTAT | -69 | 7.34 |
| 247 | all4963 | cyaC | AGTGCgaagacTCTCA | GATACTCGGCTAAGACTTCCTTG**TGGGAT**GC | -452 | 7.34 |
| 248 | all3753 | - | AGAGGatttaaGCGCA | CACTAACTAAGTTCGATTGCTGAAA**TTTATT** | -174 | 7.34 |
| 249 | alr5279 | - | TGGGAtggcgtTCACA | TCGCTCTTACTGTCTTTATTT**TGCCTT**GTGC | -134 | 7.34 |

**Table S8**. Predicted CRP binding sites in *Prochlorococcus marinus* MIT9313 genome at *P* < 0.01

| Rank | Transcription Unit | Names | CRP TFBS | Downstream region of the CRP TFBS | CRP site position | Score |
| --- | --- | --- | --- | --- | --- | --- |
| 1 | PMT1007 | - | AGTGAtggtctTCACA | CTATATTTAGGTTTGAATGCATT**TATATT**GA | -773 | 8.33 |
| 2 | PMT1006 PMT1005 | - - | TGTGAgcgagtTCACA | TTGAATTGAGATTAAGACCCC**TAGCTG**AGAG | -631 | 8.11 |
| 3 | PMT2221 | - | AGTGTtggggaTCACT | CCGTCAACGACTCCTTCACTGGG**TACTGT**TT | -296 | 7.95 |
| 4 | PMT1008 | - | TGTGAactcgcTCACA | CATCAAAGCCTGAACACCGGC**TAGAGC**GATG | -565 | 7.92 |
| 5 | PMT0035 | - | GGTGAcgttgaTCACG | ATGGGTCGCTGTCCATCAGCAG**CGCATT**GGA | -648 | 7.86 |
| 6 | PMT2025 PMT2024 PMT2023 PMT2022 PMT2021 PMT2020 PMT2019 PMT2018 PMT2017 PMT2016 PMT2015 | - - - - gltA ndhA ndhI ndhG ndhE ppnK - | TGTGAatcgccTCTCG | CAGTAAAGATCCGCTCAGAT**TGAGCT**GCGGT | -673 | 7.85 |
| 7 | PMT0934 | - | AGTGAatttgtTCACA | AGCAGCAATAACATATCCA**TTTTTT**CTGCAA | -405 | 7.82 |
| 8 | PMT1621 PMT1620 PMT1619 | - - - | TGTGGgatgtaGCACC | GTCTTTGCTGATACCAAGCACCT**TGATGT**CA | -218 | 7.74 |
| 9 | PMT0992 | hli7 | TGTGAggttttATACA | GCATTCTTTTATAGTTTTTCT**TATCTT**TATC | -57 | 7.73 |
| 10 | PMT1039 | - | TGTGCtcgtcaACACG | TTCTCTCGCTAGGTCTCTATGGTCC**TCCACT** | -262 | 7.66 |
| 11 | PMT0265 PMT0266 PMT0267 | - - - | TGTGAgctcgaTCACA | GGGATCAATGATTCCGGTCACAGG**TGTAGT**C | -78 | 7.66 |
| 12 | PMT1047 | - | CGTGAtcttgcTCACA | TGATGGGTTGAACAAGAAGAATG**CAAGGT**CT | -636 | 7.62 |
| 13 | PMT1457 PMT1458 PMT1459 PMT1460 PMT1461 PMT1462 | - - - nadD – ald | GGTGTaatgcaACACG | CAGGCGTTATCGCCGCCGGC**CACGAT**CGATC | -664 | 7.53 |
| 14 | PMT0359 | - | AGTGAatcccgGCACT | ACCCTTGCGCGATTCAAT**TAGGTA**CCCCATC | -54 | 7.47 |
| 15 | PMT0842 | - | TGTGGctgtttTCCCT | TCCGGTCACTAGAGCTTT**TAAACT**GCCAATC | -385 | 7.44 |
| 16 | PMT0916 PMT0915 | - - | TGTGAgcagaaTCACA | GCATATCTTGTCTCTGATCACT**GCTGAT**CAG | -519 | 7.43 |
| 17 | PMT2135 | argJ | AGTGAgcttgaTCACA | CTGATATGCCAAGTAAAACTTTG**TTTTTT**GA | -680 | 7.41 |
| 18 | PMT0796 | - | GGTGAacccctTCACG | CTATTTTTAGAAAAATTCCC**TCCTGT**AGAAG | -578 | 7.40 |
| 19 | PMT1221 | - | TGTGAtcttgaTCACA | GTTGAGTAGTGGCATTCATCT**TCTGTT**TGAT | -559 | 7.37 |
| 20 | PMT1493 | BioA | TGTGAccaacaTCACA | AGCAGCAATGACTGAGATCAA**TGACCT**AGTC | -509 | 7.34 |
| 21 | PMT0939 | - | GGTGAatgcggGCACT | GCACAAGTGAACAGAAAT**TGGATT**CACCATT | -187 | 7.32 |
| 22 | PMT1665 PMT1666 PMT1667 | psbB psbT - | AGCGAcaacctTCTCC | ACACTTAATGGTGCACGTCT**TACGAT**CTGCG | -80 | 7.30 |
| 23 | PMT2080 | - | AGTGAtctctaTCTCA | TCATCTCTTGCCATGCAGCAACG**GATTGT**TC | -488 | 7.28 |
| 24 | PMT1492 | - | TGTGAtgttggTCACA | GCTTTTAATAGCAATTACCACAA**TAAAGT**AT | -331 | 7.27 |
| 25 | PMT0037 | - | AGTGGttgcttTCGCG | CTTGCTAGTAAGGCAATGGGGCG**TAAATT**AG | -48 | 7.27 |
| 26 | PMT1561 | - | GGTGActaatcTCAGT | ATTTCAAAACTCTCGAAACCATT**TATTAT**TG | -231 | 7.25 |
| 27 | PMT0294 | - | AGTGAagtcaaTCTCA | GATGTGTTCCATTAAGGCGT**TCTGGT**TGCTA | -73 | 7.24 |
| 28 | PMT1940 PMT1941 | - - | TGTGAgcaagaTCACA | GCAATATGTAAGGCAGAACATA**TACCCT**TCA | -95 | 7.24 |
| 29 | PMT2110 | - | GGTGAcgccttCCACC | AGTTCCCGTACTTCAGCGCCGAAA**TAATCT**T | -411 | 7.23 |
| 30 | PMT1569 | phoN | AGTGAtctgaaGCACA | GCAGTATCTGATCTTTCTCACGGTC**TCTGCT** | -334 | 7.21 |
| 31 | PMT2077 | - | GGGGAaagcgaTCACC | TCTAATCAAAAGAGCATCG**TAAACT**TGCTTA | -461 | 7.21 |
| 32 | PMT2138 | - | TGTGAttaactTCACA | GACCAGTGTGAGCAAAGCCAACA**AGACGT**AG | -97 | 7.21 |
| 33 | PMT2193 | - | GGTGAaaaacaCCACT | CTCCTCCCATGCGCACTGCCAGC**GACTCT**CC | -639 | 7.18 |
| 34 | PMT0911 PMT0910 | - - | CGTGAatgcgaACTCT | CTACATCAGTGAACAGTGCGAAT**CACCAT**GC | -116 | 7.18 |
| 35 | PMT1656 | - | AATGTtgtcagTCACG | TGAAAGTGGCTTTGATGCC**TAAATT**GTCTTC | -37 | 7.17 |
| 36 | PMT0152 PMT0151 PMT0150 PMT0149 | - - - - | GGTGCttgggcGCACA | AGTGGGGATCAGTAAAACCG**TTTCCT**----- | -27 | 7.17 |
| 37 | PMT1578 | - | TGTGAtcttgcTCACA | AGCACCTCTATGTTTTTCGTTCGC**AGACCT**G | -686 | 7.12 |
| 38 | PMT0514 | MTG10.17 | AGCGAggatctTCACT | GTTTAGATGCTGACGTAAG**TGCAGT**ATTAGA | -325 | 7.09 |
| 39 | PMT0652 PMT0651 PMT0650 PMT0649 PMT0648 PMT0647 | - - - - gatA dnaE | CGTGGtgcggaTCACT | CAGTATCACTACACTTCG**TGCCAT**TGCGTCC | -112 | 7.09 |
| 40 | PMT0383 | - | AGTGAcaatggTCAAC | ATGAGGCTATTAATATGGACA**TTTTTT**GTAT | -180 | 7.07 |
| 41 | PMT0986 | - | GGTGCtctgggTGACG | TTGGCAGACACCAAGTATCTGCCTA**TCGAGT** | -114 | 7.01 |
| 42 | PMT0941 | - | AGTGCccgcatTCACC | TGCGCATAAAGGTCTGCTG**TTGCTT**CTGGGG | -551 | 7.00 |
| 43 | PMT0193 | - | TGTGAtcaccaCCACC | CTCCAAAGCAAGACTAAAAGGACC**AACCTT**T | -139 | 7.00 |
| 44 | PMT0845 | - | AGTGTtcagatGCTCA | CAGTCCACACAAGTTCTTGT**TCAGGT**CTGTA | -107 | 7.00 |
| 45 | PMT0622 | - | AGTGGctcaagGCGCT | GTTCAGGAGCAAGAGGTTCAAT**CAAGAT**GAC | -645 | 6.99 |
| 46 | PMT0785 | - | AGTGAttttctGCACA | GTCTTTTGAGGTGATAATTTTCTC**AATGCT**G | -154 | 6.98 |
| 47 | PMT0213 PMT0212 PMT0211 PMT0210 PMT0209 | lrtA - - - - | GGTGAgtcaccTCACC | ACCCCGATCAATGCGATGCAACGCA**GCAGGT** | -288 | 6.93 |
| 48 | PMT0487 PMT0488 | - - | TGTGAtcaaggTCAAG | TGTCTTCGATGTATGACTGTGATT**TACCAT**G | -305 | 6.93 |
| 49 | PMT0328 | - | TGTGAagcaagTCACA | AGCCGAGCAACCTAATTAAGCGA**TTGTTT**GT | -78 | 6.93 |
| 50 | PMT2054 | - | TGTGAttgtggTTGCT | AGGCAAGACCACCACAATGGTTT**TGATTT**GG | -229 | 6.92 |
| 51 | PMT2045 PMT2046 | - - | GGTGCtgcagcTCACT | GACTCCGTGTGAACGTTGAGG**TCATAT**GACC | -302 | 6.92 |
| 52 | PMT1570 PMT1571 PMT1572 PMT1573 PMT1574 PMT1575 | - - - - - - | TGTGCttcagaTCACT | GATAGTTAGCTTGATTCAGGG**AATTAT**---- | -28 | 6.92 |
| 53 | PMT1524 | - | AGTGAcctgccTCCCG | AAGGAGGTACCCGCCAGGCATCAA**GACAAT**C | -201 | 6.90 |
| 54 | PMT0884 | - | GGGGCtaggtaTCACC | TCCTGAACGTCTCGAATACGGT**TTGCGT**CAA | -298 | 6.90 |
| 55 | PMT0983 | - | CGTGAtgtctaTCGCC | AACCGGCCCTGGTTCTCACGACCTT**TTTGGT** | -84 | 6.90 |
| 56 | PMT0943 | - | AGTGAttaactTCACA | GCAGGCGTTGACCTGGTTCA**TCTCAT**GGCCT | -92 | 6.89 |
| 57 | PMT0964 | - | TGTGCtcaaacTCACT | GTTGGCATTGAGCCATGTCAC**TGCGTT**TTTA | -472 | 6.87 |
| 58 | PMT2081 PMT2082 | - - | CTTGAaagtaaTCACC | CCATTTCCAAGCGACCCACCTCA**TACTTT**GA | -110 | 6.86 |
| 59 | PMT0797 | - | AGTGAtttcatCCACC | TCTATGCAAAGGCACAGTTTGCTGA**TAAAAG** | -147 | 6.84 |
| 60 | PMT2076 | - | AGTGAttttgaTGACT | GATCACTGCTGAGGCACCAAAGTGA**TCAAAT** | -295 | 6.84 |
| 61 | PMT1917 | uvrD | GGTGCattcctTCCCT | GTTTTGGTAATACCGAATT**TAAGGT**GAGCCC | -45 | 6.84 |
| 62 | PMT1223 | - | TGTGAtcaagaTCACA | GGATTAGTTGAGACGGGTCACAAC**CAGATT**G | -179 | 6.83 |
| 63 | PMT0879 PMT0880 | - - | TGTGAgacaggGCACC | GGCATCTTCACCAGCAATGCCA**TAGACC**CCT | -142 | 6.83 |

**Table S9**. Predicted CRP binding sites in *Prochlorococcus marinus* MIT9303 genome at *P* < 0.01

| Rank | Transcription Unit | Names | CRP TFBS | Downstream region of the CRP TFBS | CRP site position | Score |
| --- | --- | --- | --- | --- | --- | --- |
| 1 | P9303_10971 P9303_10981 | - - | TGTGAgctcgtTCACA | TTGAATTGAGATCAAGCCCCC**TAGCTG**AGAG | -595 | 8.09 |
| 2 | P9303_29661 | - | AGTGTtggggaTCACT | CCGTCAACGACTCCTTCACTGGG**AACTGT**TT | -296 | 7.98 |
| 3 | P9303_10911 | - | TGTGAacgagcTCACA | CATCAGAGCCTGAACATCTGACAG**AGCGAT**G | -559 | 7.84 |
| 4 | P9303_00351 | - | GGTGAcgttgaTCACG | ATGGGTCGCTGTCCATCAGCAG**CGCATT**GGA | -648 | 7.84 |
| 5 | P9303_26951 P9303_26941 P9303_26931 P9303_26921 P9303_26911 P9303_26901 | - - sixA gltA ndhA - | TGTGAatcgccTCTCG | CAGTAAAGATCCGCTCAGAT**TGAGCT**GCGGT | -725 | 7.83 |
| 6 | P9303_02621 P9303_02631 P9303_02641 | - - - | TGTGGgatgtaGCACC | GTCTTTGCTGATACCAAGCACCT**TGATGT**CA | -220 | 7.72 |
| 7 | P9303_24381 P9303_24391 P9303_24401 P9303_24411 | - - - - | GGTGAttttgcTCACA | GCTCCTCTATCGCATCAAAGGCAA**TAGGGT**C | -713 | 7.71 |
| 8 | P9303_11191 | - | TGTGAggttttATACA | GCATTCTTTTATAGTTTTTCT**TATCTT**TCTC | -43 | 7.71 |
| 9 | P9303_13021 | - | GGTGAgagagtTCACT | GCACAAGTGAACTGAACT**TGGATT**ACACCAT | -189 | 7.67 |
| 10 | P9303_10221 | - | TGTGCtcgtcaACACG | TTCTATCGCTAGGCCTCTATGGTCC**TTCACT** | -264 | 7.64 |
| 11 | P9303_28501 | - | GGTGAatgaatTCACA | CTAATTGTAGCTACATCCA**TAACAC**AAACAG | -159 | 7.63 |
| 12 | P9303_12331 P9303_12341 | - - | TGTGAcaactaGCACG | CTAATCATAAAGAAAAGTCATGA**TAGATT**CA | -471 | 7.54 |
| 13 | P9303_04941 P9303_04931 P9303_04921 | - - - | GGTGTaatgcaACACG | CAGGCGTTATCGCCGCCGGC**CACGAT**CGATC | -614 | 7.50 |
| 14 | P9303_19431 | - | AGTGAatcccgGCACT | CCCCTTGCGCGATTCAAT**TAGGTA**CCCCATC | -56 | 7.45 |
| 15 | P9303_20811 | - | TGTGAgcttaaTCACA | GGGATGAATGATCTAGGCCA**CAGCGT**TAGTC | -103 | 7.45 |
| 16 | P9303_12481 P9303_12471 | - - | TGTGAgcagaaTCACA | GTATATCTTGTCTCTGATCACA**ACTGAT**CAG | -392 | 7.42 |
| 17 | P9303_15451 | - | GGTGAtagcaaTCACA | ACTCCCCAGCTGCGAAAACAA**TGAAGT**CAAG | -263 | 7.32 |
| 18 | P9303_17661 P9303_17671 | - potA | TGTGAtaaatgTCACA | CATAAAGCAAGCATCGATCCGTT**CCTGAT**GA | -61 | 7.32 |
| 19 | P9303_27691 | - | AGTGAtttctaTCTCA | TAGTCTCTTGCCATGCAGCAACG**GATTTT**TC | -489 | 7.29 |
| 20 | P9303_04481 P9303_04491 | fer - | TGTGAcaatcaACACG | TCAGCTCTTGAAGATGGAGGA**TATTAT**TTGG | -664 | 7.27 |
| 21 | P9303_22121 P9303_22131 P9303_22141 P9303_22151 | psbB psbT - - | AGCGAcaacctTCTCC | ACACTTAATGGGGCACGTCT**TACGAT**CTGCG | -80 | 7.27 |
| 22 | P9303_00371 | - | AGTGGttgcttTCGCT | CTTGCTAGTAAGGCAATGGGGCG**TAAATT**AG | -47 | 7.24 |
| 23 | P9303_06191 | - | TGTGAtcctgcTCACT | CGCTATCTAGCCACTTCTTGATTC**TATTTT**C | -359 | 7.22 |
| 24 | P9303_12401 P9303_12391 P9303_12381 P9303_12371 | - - - - | CGTGAatgcgaACTCT | CTACATCAGTGAACAGTGCA**AATCAT**CATGC | -117 | 7.21 |
| 25 | P9303_21991 | - | AATGTtgtcagTCACG | TGAAAGTGGCTTTGATGCC**TAAATT**GCCTTC | -37 | 7.17 |
| 26 | P9303_29161 | - | GGTGAaaagcaCCACT | CTCCTCCCATGCGCACTGCCAGC**GACTCT**CC | -639 | 7.15 |
| 27 | P9303_01891 P9303_01881 P9303_01871 P9303_01861 | - - - - | GGTGCttgggcGCACA | AGTGGGGATCAGTAAAACCG**TTTCCT**----- | -27 | 7.14 |
| 28 | P9303_10961 | - | GGTGCcagctgTCAGA | TCTCAGCTGCCGCCAATTCTT**TATCCT**TCTC | -375 | 7.12 |
| 29 | P9303_13921 | - | GGTGAttaataTCACT | CCTGATTCATTTAGCGATGGTGGC**AATTAT**C | -529 | 7.12 |
| 30 | P9303_11231 P9303_11221 | - - | GGTGGtcaaccTCTCG | GCGACAAGACTAAAGTCTCCCT**TAGATT**ATG | -449 | 7.09 |
| 31 | P9303_11751 P9303_11741 P9303_11731 | - - - | GGAGAtagacaTCACC | CCCTGAACGGCTCGAATACGGT**TTGCGT**CAA | -53 | 7.06 |
| 32 | P9303_15781 P9303_15791 P9303_15801 P9303_15811 P9303_15821 P9303_15831 | - - - - gatA dnaE | CGTGGtgcggaTCACT | CAGTATCACTACACTTCG**TGCCAT**TGCGTCC | -57 | 7.06 |
| 33 | P9303_02441 P9303_02431 P9303_02421 P9303_02411 | - - - hrpB | TGTGAccaaatACGCC | TGAGCTCGTCTTCATGAAGC**TTCATT**CCCCC | -248 | 7.05 |
| 34 | P9303_14011 | - | CGTGTgcaggtTCACT | GGGAGTTGCCAATTGATTACCCA**TTGCCT**CG | -82 | 7.03 |
| 35 | P9303_04171 | - | AGTGAcctgccTCCCG | AAGGAGGTACCCGCCAGGCATCAA**GACAAT**C | -201 | 7.02 |
| 36 | P9303_01771 | dedA | TGTGCtactggTCACC | CCAGCTTTGTGATGAGCAAT**TCCTTT**ACCAA | -737 | 7.02 |
| 37 | P9303_19111 | - | AGTGAcaatggTCAAC | ATGAGGCTATTAATATGGACAGT**TTTTGT**AT | -182 | 7.01 |
| 38 | P9303_02951 P9303_02941 | - - | TGCGAtgtctaACACA | ACAAAGTCTGGCTAGCCAAG**TAAATT**TTCTC | -153 | 7.01 |
| 39 | P9303_11401 | - | GGTGCtctgggTGACG | TTGGCAGACAGCAAGTATCTGCCTA**TCGAGT** | -116 | 6.99 |
| 40 | P9303_16161 | - | AGTGGttcaagGCGCT | GTTCAGGTGCAAGAGGTTCAAT**CAAGAT**GAC | -643 | 6.97 |
| 41 | P9303_13281 P9303_13271 P9303_13261 | - - - | AGTGCtgtaggTCACC | ACAGTTCTCGGTCGCATAAG**TTCCAT**AGAAA | -781 | 6.96 |
| 42 | P9303_25761 | icd | AGTGAgggcttGCACA | ACTGGTGCACTGAGCAAACGAAAA**TCTCCT**G | -680 | 6.96 |
| 43 | P9303_03591 | - | GGTGAccactcTCAGC | ATTTCAAAAGTCTCAGAGCCA**TTTTTT**ACTG | -40 | 6.95 |
| 44 | P9303_12311 | - | CGTGCtattgaGCCCA | GCCCAGCAGATGTGGCACCATCAT**TAAGAT**A | -44 | 6.92 |
| 45 | P9303_22711 P9303_22721 P9303_22731 P9303_22741 P9303_22751 | psaD - - ppc - | AGTGCtactttTCACC | AGTAACGGTATGCCGATTCTC**GAGGTT**ACTC | -33 | 6.91 |
| 46 | P9303_27271 P9303_27261 | - - | TGTGAttgtggTTGCT | GGGCAAGACCATCACAATGGTTT**TGATTT**GG | -243 | 6.90 |
| 47 | P9303_17831 P9303_17821 | - - | ATTGAttcactTCACT | CAATAGAGTTCAGCCTTCCCATTGA**TAACTT** | -491 | 6.90 |
| 48 | P9303_12671 | - | TGTGAtcaacaTCACA | ACCACCCATGACACAGCTCATCAAC**CCATCT** | -703 | 6.89 |
| 49 | P9303_29361 P9303_29351 | - - | GGTGAtcaagtCCACC | CTGACCTCTTGGGGCACAA**TTGTCT**CTAGGG | -481 | 6.88 |
| 50 | P9303_06491 | - | TGTGAtctacaTCACA | TCTCTAAGTCTTCTGCAGAGCCT**GGCGAT**CA | -738 | 6.86 |
| 51 | P9303_27701 P9303_27711 | - - | CTTGAaagtgaTCACC | CCATTTCCAAGCGACCCATCTCA**TACTTT**GA | -110 | 6.85 |
| 52 | P9303_03421 P9303_03431 | - - | GGTGAcacccgCCACG | GCGGGGTCGAAGCGCAGCCG**TTGAAT**CCGAT | -465 | 6.84 |
| 53 | P9303_04111 | - | TGTGAtcaatcTCACA | ATCGGGTTTGACTCAGAACAATG**CATAGT**CG | -75 | 6.83 |
| 54 | P9303_12031 | - | CGTGAtcttgcTCACA | TGCCCCTAGCCGCAACCCATA**TCCGTT**GAGC | -245 | 6.82 |
| 55 | P9303_00661 | - | TGTGAggaaaaGCACA | GTATTTCATGATCAAGAA**CAATAT**CTAACTA | -93 | 6.82 |

**Table S10**. Predicted CRP binding sites in *Synechococcus sp.* CC9311 genome at *P* < 0.01

| Rank | Transcription Unit | Names | CRP TFBS | Downstream region of the CRP TFBS | CRP site position | Score |
| --- | --- | --- | --- | --- | --- | --- |
| 1 | sync_1260 | - | TGTGAcgaagaTCACA | CACTAATTTGATGAGCATC**AACACT**AAACAA | -98 | 7.32 |
| 2 | sync_2863 | - | AGTGAggagccTCCCC | ACATCGCCAAATCCACGTTGATGG**TGTGAT**G | -239 | 7.26 |
| 3 | sync_0351 sync_0352 sync_0353 | - coaBC psbO | TGTGAttttgcTCACT | ACGTACAAGAGAAGATTCGATGA**TCTGCT**CT | -291 | 7.23 |
| 4 | sync_2054 | - | GGTGAgcatccTCCCA | AGAACGACAGCCTCTAGTTCAT**TGCCAT**CGG | -142 | 7.08 |
| 5 | sync_1414 | - | GGTGAgcgctcTCACA | GAGAGCTGCACATCGGGGAAATTCA**CCCCCT** | -457 | 7.06 |
| 6 | sync_1258 | - | TGTGAagttatTCACT | GCTGGCTCCTGGATTTTTTCTTC**TAGCAT**CT | -606 | 7.04 |
| 7 | sync_1504 | - | GGTGAtaagtcGCACG | TCAAGAGAATACCGATAGACT**TCAGTT**TCGG | -127 | 6.97 |
| 8 | sync_0219 sync_0220 sync_0221 sync_0222 sync_0223 | - - - - - | TGTGAtattggCCTCA | TTTATTCAGGGGCTTATGGA**TATTTT**TTTAT | -41 | 6.93 |
| 9 | sync_1496 sync_1495 sync_1494 sync_1493 | - - - - | AGTGAttcaacTCTCA | CTTTGAAACAACGTGCTTTGGTCAT**CACCCT** | -89 | 6.92 |
| 10 | sync_0922 | - | GGTGAtccagaTCTCA | AGACGAAACGTTTGATCGCCCAACT**CAGGAT** | -236 | 6.90 |
| 11 | sync_1014 | - | GGTGAaggagtACACC | CACCCGCCGATCCCTTTCGCTTC**TGGAGT**GA | -64 | 6.89 |
| 12 | sync_0038 | - | TGTGGgggccaGCGCA | CTACGGAGCTGTGTAGAGGAC**TAGCGT**CTGA | -266 | 6.87 |
| 13 | sync_0931 | - | TGTGAtaaatcTCACA | GTTCATGTTGATTGAAGCCGGAACA**TCTGAT** | -46 | 6.87 |
| 14 | sync_1291 | - | TGTGTatgccaTCACG | AGTACTTGATTTGATCTTATGATTT**TATAAT** | -294 | 6.86 |
| 15 | sync_0797 | - | AGTGGcgggcaGCACG | CACTGAGTCCTGAGCACTAA**TTCTAT**ACAAC | -274 | 6.86 |
| 16 | sync_1615 | - | GGTGAtaaccaCCACG | GATGGGCGGTTCTACCTA**TCGCAT**CTGGCTC | -266 | 6.84 |
| 17 | sync_2526 sync_2527 | - hrpB | AGTGAgaatccTCCCT | GAGCTCAGCTAAACATGAA**TTCAAT**CCGGCC | -47 | 6.79 |
| 18 | sync_1529 sync_1530 sync_1531 | glgA - - | CGTGAaagcgaTCACA | ACGCGGTAATGCGTCTACAGTCTCG**CGGCTT** | -49 | 6.79 |
| 19 | sync_0798 sync_0799 | - - | CGTGCtgcccgCCACT | GCAATTTCAAAAATATGAATCTCA**GAGACT**T | -133 | 6.79 |
| 20 | sync_0277 | - | GGTGAccacatCCTCA | AGAACGGTGACGAGCGCGCCC**TGCTCT**GGCA | -349 | 6.78 |
| 21 | sync_2270 sync_2269 sync_2268 | - - - | AGTGAtccagaTCCCT | TCCTCCAAGGGCGATCCCGGCT**TTCCCT**AAG | -777 | 6.77 |
| 22 | sync_1232 sync_1233 sync_1234 | - envZ - | AGTGAcccattTCTCC | ATTACAGAAGACTATGGAG**TGTGAT**CGGTGA | -176 | 6.77 |
| 23 | sync_2605 | - | GGTGCgagccaTCACA | ACGGTCAAGCGAGTCCCTGTG**AACCCT**CCCG | -535 | 6.76 |
| 24 | sync_2604 sync_2603 sync_2602 | - - - | GGTGAgagcaaTCGCG | TTGATTCGATAGTCACGTCGCA**TGAGAT**CAG | -351 | 6.73 |
| 25 | sync_2749 | - | TGTGAtgttgaTCACA | GTGTTCGGAAAACTTGTTATGA**CAGGAT**TTG | -77 | 6.71 |
| 26 | sync_1931 | - | TGTGAattgacTCGCC | AATCATCCCTGAGCTCTATTGGCA**TCGATT**G | -87 | 6.71 |
| 27 | sync_0060 sync_0061 | - - | GGTGCgatccaCCACA | GCCTTGGTCTTCAAATGACGCT**TCTGTT**TCG | -89 | 6.70 |
| 28 | sync_2837 | - | AGTGAgaacccTCACT | TCAACGCCACCGTTGAGATCA**TTTTCT**AGCT | -136 | 6.70 |
| 29 | sync_0208 sync_0209 | - - | GGTGAtcggcaCCACC | TTCCTGGCACCGGTGCTGC**TCAGGT**TGGTGA | -487 | 6.69 |
| 30 | sync_0159 | rfbB | TGTGAtccgcaGCCCT | AGTTTGTTTTTTAAAGAGCAA**TACATT**CCAG | -98 | 6.69 |
| 31 | sync_0450 | - | AGTGCcatggaTCACC | TGACGTTGCAGGTTGGAGGGCTCAA**CAAGGT** | -785 | 6.69 |
| 32 | sync_1512 | - | TGTGTcagtagTCCCA | GACAATGACGGCCACCGTTCCAACA**AACCGT** | -383 | 6.68 |
| 33 | sync_0911 sync_0910 | - trxB | TGTGCgaaatcACACA | CGATGTTGGTCCTCCAACAGGCAA**TGTGGT**C | -49 | 6.67 |

**Table S11**. Predicted CRP binding sites in *Synechococcus sp.* CC9605 genome at *P* < 0.01

| Rank | Transcription Unit | Names | CRP TFBS | Downstream region of the CRP TFBS | CRP site position | Score |
| --- | --- | --- | --- | --- | --- | --- |
| 1 | Syncc9605_1714 | - | TGTGAtcaagaTCACA | AGCAGCACTGTATGGGGTCA**TCAACT**GACCG | -104 | 7.36 |
| 2 | Syncc9605_0559 Syncc9605_0560 | - - | TGTGAtcaagaTCACA | GGTGTTGCATTAGTTAACCGC**GATCAT**TGGG | -200 | 7.34 |
| 3 | Syncc9605_1000 Syncc9605_0999 Syncc9605_0998 Syncc9605_0997 Syncc9605_0996 | - - - - - | AGTGAaatagcGCACA | AAGTATTAATTCGGGAGTCTT**TATTAT**GACC | -78 | 7.27 |
| 4 | Syncc9605_2504 Syncc9605_2505 Syncc9605_2506 | - - - | CGTGAcccacaTCACC | GCGACAGGGTCATAAAGGGTAGA**GAATTT**AA | -50 | 7.17 |
| 5 | Syncc9605_1249 | - | AGTGAaaaaacTCACT | ACAATGAATACAACAAAAAACAA**TAAGTT**GA | -38 | 6.90 |
| 6 | Syncc9605_1301 | - | TGTGAtggactTCGCT | TCCGCTGCGATAATTGTACAGCA**TGCTTT**TC | -530 | 6.88 |
| 7 | Syncc9605_2489 | argJ | TGTGAgcttgaTCACA | GCTCTGCTGTGGGGCTCATCTTCA**GCAGTT**C | -633 | 6.77 |
| 8 | Syncc9605_0534 | - | CGTGTtcgcccTCACA | GACAAGCTGTCTGGTCTGTGGG**AATGTT**CGT | -32 | 6.75 |
| 9 | Syncc9605_0955 | - | TGTGAccaacaTCACA | CGAGCGGATAACTGGCGTCA**TCAGTT**GAGTC | -76 | 6.73 |
| 10 | Syncc9605_2507 | - | TGTGAtcaacaTCACA | GACCCCCTTGATCAACATCAACACA**TCACTT** | -711 | 6.73 |
| 11 | Syncc9605_2607 | - | GGTGAtcaagaCCACA | TCGCGGCCATCCCAGCGCTCCG**TGGGTT**CTG | -196 | 6.73 |
| 12 | Syncc9605_0452 Syncc9605_0453 Syncc9605_0454 Syncc9605_0455 Syncc9605_0456 Syncc9605_0457 | - - - - - - | TGTGAgcaaacTCACA | GCCCTGCATGAGGCAGGTCA**TCAACT**GGATC | -683 | 6.71 |
| 13 | Syncc9605_0647 Syncc9605_0648 Syncc9605_0649 | - - - | GGTGTtgacccACACT | TCCTTCGGGCTGACCTGCGGAGA**TGCTCT**GC | -427 | 6.69 |
| 14 | Syncc9605_2093 Syncc9605_2092 Syncc9605_2091 | - - - | GGTGAagccccCCACG | TGCAGCTCGTAGATCACCG**TGCGTT**GCCAGC | -529 | 6.66 |
| 15 | Syncc9605_1034 Syncc9605_1033 Syncc9605_1032 Syncc9605_1031 Syncc9605_1030 Syncc9605_1029 | - - - - - - | CGTGAagcgctACACC | AGCGCCGCTGCCGGGGCTTA**TCAATT**TCTGC | -659 | 6.66 |
| 16 | Syncc9605_1764 Syncc9605_1765 | - - | AGTGGtccgcgTCACC | TCCGACGCACCGACATCACATGAGG**TGATGT** | -295 | 6.65 |
| 17 | Syncc9605_0769 | - | AGTGAaaggcaTCGCT | CCGCTCAGGCTCACCGAACTG**TGGCCT**AATC | -270 | 6.64 |
| 18 | Syncc9605_2608 | - | AGTGTtgagagACACT | CAAATTGACTAATTTCTCCATCCGG**CAATCT** | -288 | 6.64 |
| 19 | Syncc9605_2479 | - | AGTGCtatggcTCTCT | GTTGTTTGCGGCTAAAAATTCGG**TTTGAT**TG | -313 | 6.64 |
| 20 | Syncc9605_2259 | - | CGTGGtcgactTCACC | GCTCGGGTGTTGTCGAGGACA**GATCAT**ACCG | -106 | 6.63 |
| 21 | Syncc9605_1001 Syncc9605_1002 | - - | TGTGCgctattTCACT | CTTTCTTCCTGTTGGTGAAGTG**AATCTT**TCA | -85 | 6.63 |
| 22 | Syncc9605_1587 | - | GGTGGgggcaaTCACC | ATTGGGCTCTGATAACACCACTGAC**TCCCTT** | -477 | 6.63 |
| 23 | Syncc9605_1050 | - | GGTGAgcaaggTCACA | GTCCCTTGTGAGCTCTGTCAACGG**TTCAGT**A | -47 | 6.62 |
| 24 | Syncc9605_2332 Syncc9605_2331 Syncc9605_2330 Syncc9605_2329 | uppP - - - | TGTGAagaaagTCACG | GAACCTTAGAAGACATCT**CCAAGT**GACGCGG | -86 | 6.59 |
| 25 | Syncc9605_0525 | - | GGTGAtggggaTTACT | TGGCCTAATCCTCCGTTCAACG**GAGGTT**TTT | -270 | 6.59 |
| 26 | Syncc9605_2358 Syncc9605_2359 Syncc9605_2360 | - - - | TGTGAgcaaagCCACG | TGGGAATCTGAGTAGAGACCAC**TGCCCT**CTC | -37 | 6.58 |
| 27 | Syncc9605_0218 Syncc9605_0217 | - - | GGTGAgttcgcGCACC | TTGGCCTCAAAACGGCCCT**TGTCAT**CAGGCA | -235 | 6.57 |
| 28 | Syncc9605_0529 | - | CGTGAtgaacaGCGCA | ACCGGGCTTGGGTGCCCTATATT**TAGTTT**GT | -470 | 6.57 |
| 29 | Syncc9605_0406 | - | AGTGTggccaaTCACC | TCCACAGCAAGTCTCAAGAC**TCTCCT**CTTGA | -77 | 6.57 |
| 30 | Syncc9605_1878 | - | CGTGAccttcaGCACC | GGCTGGGTCATCACATCAGCCACCG**TGAGCT** | -72 | 6.56 |
| 31 | Syncc9605_2683 Syncc9605_2684 | - - | AGTGAttcagaTCACC | AATGAGATTGAACTTTGTTC**ACTCCT**CACAA | -234 | 6.55 |
| 32 | Syncc9605_0073 | - | AGTGAgggtggCCACG | ATGCGCAGAGCTGGTTCCCCTGC**TTCGAT**CA | -727 | 6.54 |
| 33 | Syncc9605_2249 | - | TGTGCtccaggTCACC | ACTCCTGTCAGAATGACAGGGTC**CGGCTT**CG | -543 | 6.54 |
| 34 | Syncc9605_1709 | - | AGTGAaagactGCACT | CCTCGACTTGAGACGCCTTA**TAAATT**TCAAT | -110 | 6.54 |
| 35 | Syncc9605_0843 | - | GGTGCtgttgaTCACC | AACCGCCGCCCGTAGGTTTCCCGCA**GATCCT** | -730 | 6.53 |
| 36 | Syncc9605_0271 Syncc9605_0272 | - - | GGTGGtgtcaaTCCCC | TTGTGGAATGTGATCTCGCCGC**TACATT**CAA | -69 | 6.52 |
| 37 | Syncc9605_2284 Syncc9605_2285 Syncc9605_2286 Syncc9605_2287 | - - - - | TGTGAccctcaACACG | GCCGACACGCCGAGGCAATTCGG**TGGCAT**CG | -55 | 6.52 |
| 38 | Syncc9605_1179 Syncc9605_1178 | - - | GGTGAtctcgaTCCCA | CCGGTGGTGAACCGCTTGGTGC**TGTTCT**CCC | -587 | 6.52 |
| 39 | Syncc9605_1251 | - | TGTGAtccacgTCACT | GATTGGCGTAATGGTATTCC**TGCGGT**GGTTG | -178 | 6.51 |
| 40 | Syncc9605_1734 Syncc9605_1733 Syncc9605_1732 | - - - | GGTGAatatcaTCACG | CCGACAGGTTACTCGACTCAAAT**TCGATT**GA | -558 | 6.50 |
| 41 | Syncc9605_0505 Syncc9605_0504 | - - | TGTGGgtccagTCTCA | CCGCAACAGGGCTTGCGTTG**TTGCTT**CTGCA | -89 | 6.49 |
| 42 | Syncc9605_1794 | - | GGTGTgcagaaCCACA | GCCGACTCAGGCCTTGAGCT**GACTCT**CGATC | -395 | 6.47 |
| 43 | Syncc9605_0488 Syncc9605_0487 | - - | CGTGAaaacacTCTCG | TTGCAGCGAATTGCTTCTTCGCAAC**GAAATT** | -112 | 6.46 |
| 44 | Syncc9605_0155 Syncc9605_0156 | - - | GGTGAtcggcaCCACG | TTCCTGGCACCGGTTTTGCTGCGCT**TGGTGT** | -483 | 6.46 |
| 45 | Syncc9605_1804 Syncc9605_1805 | - - | GGTGActgatgACTCG | GCCCGAGACCTAATCTGACG**TCCGAT**CGGCC | -48 | 6.45 |
| 46 | Syncc9605_2283 Syncc9605_2282 Syncc9605_2281 Syncc9605_2280 Syncc9605_2279 | - - - - - | CGTGTtgagggTCACA | ATGGGGAGCCCGCTGACCGGAGG**CCTGTT**GG | -89 | 6.44 |
| 47 | Syncc9605_2395 Syncc9605_2394 | - - | TGTGAacaggcGCACG | TGGCGGGTGGTGCACTTGAG**AAGGGT**GTCAG | -123 | 6.43 |
| 48 | Syncc9605_1456 Syncc9605_1457 Syncc9605_1458 | - - - | GGTGAaaccccCCACC | AGCACTGCAGGGACGCGCACCCAC**AACCGT**C | -370 | 6.42 |
| 49 | Syncc9605_1535 Syncc9605_1534 Syncc9605_1533 | - - - | GGTGCcgctgaTCGCA | CCCAATTGGCGGTAATGGCGT**TGCTTT**ACGG | -276 | 6.41 |
| 50 | Syncc9605_1513 Syncc9605_1512 | - - | CGTGAtaagtcCCACC | AGACGGCCACTTCCACTTCATCCA**TCTCAT**T | -188 | 6.41 |
| 51 | Syncc9605_0232 | - | GGTGAgaccagCCACG | GCCCGCGCATCGGCCTCCAC**GTGGGT**GAGCA | -240 | 6.41 |
| 52 | Syncc9605_2519 Syncc9605_2520 | - - | CGTGGcgcaggTCGCT | CCTGCTGGCCGAGGAACAC**TGGCTT**GAACGG | -226 | 6.39 |
| 53 | Syncc9605_1884 Syncc9605_1885 | - - | CGTGGccttcaCCACC | CAAGACCTGCTCATCGCCTTCGG**CAGCTT**CG | -396 | 6.37 |
| 54 | Syncc9605_0261 | - | CGTGAcgagcaACACT | GAAACCGACCCTGTGATCAGGGT**TCCGGT**CA | -312 | 6.36 |
| 55 | Syncc9605_1700 Syncc9605_1701 | - - | TGTGAgcaagaCCACA | GCTGCTGGTGAGGCAGGTCG**CCAGGT**GAACC | -34 | 6.36 |
| 56 | Syncc9605_2205 | - | CGTGGcggctgTTACA | GAACGCTGCCGAAGCTTGACTTCC**TAGGGT**C | -59 | 6.34 |
| 57 | Syncc9605_0695 Syncc9605_0696 Syncc9605_0697 | - - - | TGTGGctgtgaTCACG | GATCGAACCTTCCCTTCTCCGCG**ACTGAT**CC | -51 | 6.31 |
| 58 | Syncc9605_0521 | - | TGTGTgttgatTCACG | AACGGATAAACAATCTACTTCTTGC**TTATTT** | -72 | 6.30 |
| 59 | Syncc9605_1575 | - | AGTGAcaaaaaGCACA | AAATCGCGAGTCATCTGAT**CCGAGT**CAAGGC | -207 | 6.29 |
| 60 | Syncc9605_1640 | - | GGTGTccccttCCACG | CTGGGGGTGAGGGCCAGGA**TCACCT**CACTGA | -528 | 6.29 |
| 61 | Syncc9605_1451 | - | GGTGCgttcttCCACA | TCCGCCAGGGATGCCTCCAAC**TGGCTT**CTCT | -189 | 6.29 |
| 62 | Syncc9605_2642 Syncc9605_2643 | - - | GGTGAattgatTAACT | GCGCGGGTCAAACGGATTC**TCTTTT**CGACCG | -91 | 6.28 |
| 63 | Syncc9605_0533 | - | TGTGAgggcgaACACG | GAGGGATGGAACGCTGCAG**GCCCAT**CCCTCA | -355 | 6.28 |
| 64 | Syncc9605_1634 | - | GGTGAtgatcaGCGCC | AGGAAAAGGATTTCCATCGCGACG**TGCAGT**C | -59 | 6.27 |
| 65 | Syncc9605_0295 Syncc9605_0294 | - - | TGTGGttctcaGCACC | GTGGCACGACCGTCAGCCCTGC**TCACCT**TCA | -128 | 6.27 |
| 66 | Syncc9605_1621 Syncc9605_1622 Syncc9605_1623 | - - - | GGTGGtggtcaACACC | CGGGTGGAGCTGAGCATTCGCTC**CACCTT**CG | -756 | 6.26 |
| 67 | Syncc9605_0956 | - | TGTGAtgttggTCACA | GGGGTCACGCCGCCGCGGTGCCGC**CGGTTT**C | -126 | 6.26 |
| 68 | Syncc9605_1486 | - | GGTGTcttcctTCACC | CTCGACTTCTGCTTCACAAA**GCCGAT**GGCGG | -294 | 6.25 |
| 69 | Syncc9605_2416 Syncc9605_2417 | - - | CGTGAgcagagCCACG | CCTTCACCGAGCTCAGCCACCT**GACGCT**GAA | -747 | 6.25 |
| 70 | Syncc9605_0494 Syncc9605_0495 | - - | GGTGAtcgccaTCGCT | GGGCATAAGGAGATGCAATTG**GAGCGT**ATCT | -39 | 6.24 |
| 71 | Syncc9605_0527 | - | GGTGCcttgagCCACT | GACGCCAGGGTCGCGACTCCCTA**TCACCT**GA | -47 | 6.24 |
| 72 | Syncc9605_1503 | - | TGTCTccattgTCACG | GCACGCCCGATGTTCGTTCGT**TAAATT**CTGT | -50 | 6.22 |
| 73 | Syncc9605_0976 | - | GGTGAtcatcaTCACG | ATCTCGCGGCGTTGTGATCG**TTTGAG**GCGCA | -193 | 6.22 |
| 74 | Syncc9605_1505 | - | CATGAagccgaTCACA | AGAAGCGCAACAGCAACTGTTGA**GGCAAT**GG | -429 | 6.22 |
| 75 | Syncc9605_0776 Syncc9605_0777 | - - | AGTGAggagaaCCGCC | ATTCCAGGATCGCAAACCCGCCCA**CACAGT**T | -396 | 6.22 |
| 76 | Syncc9605_0750 | - | TGTGAtcacatCCCCA | CAAGTCGTTGTCGACGGTGGT**GAGGCT**CCAT | -149 | 6.21 |
| 77 | Syncc9605_0975 Syncc9605_0974 | - - | CGTGAtgatgaTCACC | AGCGACGACATGAAGCTGCGGC**TCAAAC**AGC | -97 | 6.21 |
| 78 | Syncc9605_1550 | - | CGTTAcaacgcTCACA | CTTCGACAATGTCATTCGCTC**AAGCTT**CTTG | -122 | 6.20 |
| 79 | Syncc9605_0023 Syncc9605_0024 Syncc9605_0025 Syncc9605_0026 | - - - - | CGTGCttgctgTCTCT | GCCGGAGCAGGGTTGCTTAAC**TCTTCT**GTGG | -493 | 6.19 |
| 80 | Syncc9605_1067 Syncc9605_1066 | - - | GCTGAcgatgaTCACT | GTGTCAAGCAGTGAACGTGGATCGA**TCCAAT** | -61 | 6.19 |
| 81 | Syncc9605_1905 | - | GGTGAcgaggaCCTCT | GGTGGATCCAGCGTCGAGGCTTC**TGGTTT**TT | -486 | 6.18 |
| 82 | Syncc9605_1235 | - | GGTGTatcgctCCACC | AATGGCTGGATGTCCTTGTC**TACCTT**GCGTG | -140 | 6.18 |
| 83 | Syncc9605_0045 Syncc9605_0046 | - guaA | GGTGGtgcgccGCACG | ATGGCCTGGAACTCCCCGC**TGCGGT**GGCCAA | -153 | 6.18 |
| 84 | Syncc9605_0950 Syncc9605_0949 | - - | TGTGActgactTCTCA | TAAGTTTGGATGATGTGGGTCA**TGGGTT**GTG | -307 | 6.17 |
| 85 | Syncc9605_1883 | - | GGTGGtgaaggCCACG | GCACTCATTACAAAACTTTGTTTAC**TAATGT** | -284 | 6.16 |
| 86 | Syncc9605_2485 | - | AGTGCaatcgcGCGCT | TCATGTGGTCAGCCCTTAGAG**GAGTTT**TCAA | -218 | 6.16 |
| 87 | Syncc9605_1422 | - | AGTGGtgcccgTCACT | GATGCGCCCTCAGGAACTTTACGCA**TTGCCT** | -635 | 6.16 |
| 88 | Syncc9605_1209 | - | TGTGGtgatggGCACT | GGCATGGGTTGAGAGATC**AAGGCT**CCCCCCC | -444 | 6.15 |
| 89 | Syncc9605_0485 Syncc9605_0486 | - - | AGTGTtcgaaaTCCCA | TGCGCCGCAGCCAGTCTGAAA**GATCGT**TAAC | -112 | 6.15 |
| 90 | Syncc9605_2028 Syncc9605_2027 Syncc9605_2026 Syncc9605_2025 Syncc9605_2024 Syncc9605_2023 | - - - - - - | GCTGAccggaaTCACA | CGAACCACCTCCTGATCGC**TGTTTT**AGACGG | -213 | 6.15 |

**Table S12**. Predicted CRP binding sites in *Synechocystis sp.* PCC6803 genome at *P* < 0.01

| Rank | Transcription Unit | Names | CRP TFBS | Downstream region of the CRP TFBS | CRP site position | Score |
| --- | --- | --- | --- | --- | --- | --- |
| 1 | ssr2333 slr1392 | - feoB | AGTGAttatacTCACA | ATTATTCCAGTGATTTTTGATATT**TATTCT**G | -86 | 8.65 |
| 2 | sll1543 | - | TGTGAcccagaTCACA | ACTGTTGTGTATCTAGATCAAGA**TAGGGT**TT | -181 | 8.30 |
| 3 | sll1247 | - | TGTGAtctagaTCACC | TCCCATGGGTGCTTGCATCACT**TACATT**GGA | -151 | 8.13 |
| 4 | **slr2015 slr2016 slr2017 slr2018** | - - - - | GGTGTttattgTCACA | GGTTTTGGTGAGCTGGCTAACAA**TCATTT**GT | -346 | 7.98 |
| 5 | slr1351 | murF | GGTGAtctagaTCACA | GATAAAAATTGCAAATGACT**TAACAT**TCCTG | -87 | 7.94 |
| 6 | slr1926 slr1927 slr1928 slr1929 | - - - - | CGTGAcagcccCCACG | AAGGAAGCGACGATCGCCGTTTC**TAGGGT**CA | -574 | 7.79 |
| 7 | sll0536 sll0537 | - amt3 | AGTGTccttcgCCACA | ATTTTCTAAATTAGGGTTGA**TAAACT**GCCTA | -89 | 7.52 |
| 8 | sll1520 sll1521 sll1522 | recN - pgsA | TGTGAtctggaTCACA | GCCCTCCAACCATTCATTA**AACCCT**AAGCCT | -561 | 7.52 |
| 9 | sll1577 sll1578 sll1579 sll1580 ssl3093 | cpcB cpcA cpcC cpcC cpcD | GGTGAtttagaTCACA | GACAAACCACAGGGACGTTGCT**TGGGCT**AAA | -702 | 7.49 |
| 10 | sll1941 sll1942 | gyrA - | TGTGAccaaaaTCACT | GGTAGGCTGGCCGTGGCAGGG**TCTTGT**TTGA | -272 | 7.47 |
| 11 | sll1708 ssl3291 sll1709 sll1710 | - - gdh - | CGTGAtagtaaTCACC | GATGAAGTACAAAGAATTGCTAAG**CACTGT**A | -61 | 7.46 |
| 12 | **slr1667 slr1668 ssr2786** | - - - | TGTGAtctgggTCACA | ACCATTGAGTGATTAGGGACAA**TACATT**TCT | -245 | 7.45 |
| 13 | slr1732 | - | GGTGAttctaaTCACA | GGCGATCGGTGCCAGCTTT**AAAAGT**CTCCCT | -317 | 7.45 |
| 14 | **slr0442** | - | TGTGAtccagaTCACA | TACGTGGGTTAACCGGGATAA**TAAAAT**ACAG | -189 | 7.45 |
| 15 | sll1268 | - | TGTGAtctagaTCACA | GAGGGCCACGGCCTGGATCTC**TACAAT**GGTC | -146 | 7.43 |
| 16 | sll1874 sll1875 | AT103 ho2 | TGTGAgaataaTCACA | GGCAGTTTTTTTGTTACT**TCGATT**CAGAATC | -387 | 7.43 |
| 17 | **sll1371 sll1372 sll1373** | - - - | AGTGAaaaaacTCACT | TTCTCTGGAGTGGGGCACAAC**CAATTT**AAGG | -143 | 7.41 |
| 18 | sll1261 | tsf | GGTGTtgaccaTCACT | TTTTTCCCTTAGCCCAATAAT**TACCGT**GCAA | -61 | 7.40 |
| 19 | slr0637 slr0638 slr0639 | - glyQ - | TGTGAtcattgCCGCA | GGTTTGTTCGTTTTTGCT**TAACAT**TTAACTC | -80 | 7.36 |
| 20 | slr0869 slr0870 slr0871 | - - - | TGTGActacaaCCACA | AGCTCGACAACGGTAATTA**TCCCTT**CGGAAA | -196 | 7.36 |
| 21 | slr1400 | - | AGTGAaattttTCTCA | AGATGGCATCTTAGGAGGGAA**TGAATT**GTTC | -199 | 7.32 |
| 22 | slr1805 | - | GGTGAttactaTCACG | GTAAGGGTGCCGATGCAAAAAG**GGGGTT**CGC | -95 | 7.26 |
| 23 | sll1537 | - | GGTGAgatcaaCCTCA | AACCATTGCCCTAAAAAC**TGTTCT**GCATCGA | -193 | 7.23 |
| 24 | sll0236 ssl0438 sll0237 sll0238 sll0240 sll0241 sll0242 sll0243 | - - - - - - - - | TGAGAaaatttTCACA | CCGTTGCTCTATCGTTGCCCCCG**GAATAT**GA | -517 | 7.23 |
| 25 | sll1033 | - | AGTGAggtaatACCCA | CAAAAGTAGAGTTAGTTGAGG**TAGTGT**CTAA | -233 | 7.22 |
| 26 | ssl2069 sll1060 sll1061 sll1062 | - - - - | TGTGGttagttTCTCC | AAGGAAAGTTTGAGCAAGACAT**TAGTTT**TTT | -252 | 7.20 |
| 27 | slr2079 ssr3532 slr2080 slr2081 slr2082 slr2083 slr2084 | - - - tyrA ctaD ctaE - | GGTGGcgatcgCCACC | CAAATGTCCCGGCCGGCA**TAGCTT**TGGCCAA | -176 | 7.19 |
| 28 | sll0330 | fabG | AGTGCatttaaCCACG | CTGAAACAATTTTAGGAGGCGTT**TACCAT**GC | -410 | 7.19 |
| 29 | slr1109 slr1110 slr1772 | ank - - | GGTGGgggcgaTCGCC | GTAAATAAGGGATGTAAGCC**TAGTTT**AAAGC | -245 | 7.18 |
| 30 | slr1452 ssr2439 slr1453 slr1454 slr1455 slr1456 slr1457 slr1459 | sbpA - cysT cysW cysA - chrA apcF | AGTGCcccagtTCACG | GTCGGGCAAACCCCAGCTC**AATCAT**GACACA | -194 | 7.17 |
| 31 | slr0484 slr0487 slr0488 slr0489 slr0491 slr0492 | - - - - - menE | TGTGGgatagtTCTCC | CTTCATTGGTATTCTAGGAGT**TAGTTT**TTCG | -62 | 7.14 |
| 32 | slr1739 | - | GGAGAcatgggCCACA | GCGCAAACCCCTCCGCTCCTTGGG**TAAACT**C | -139 | 7.12 |
| 33 | slr1609 | - | AGTGCcccccaCCACG | CATTCGCTTAGCACTAAG**TAACTT**TCGGCAT | -420 | 7.11 |
| 34 | sll1094 ssl2153 | - rpiB | TGTGAaaatgaGCTCA | AGCAATATGCGCCAGAAAGATTGAC**TTATAT** | -176 | 7.10 |
| 35 | sll0634 | btpA | GGTGAaaccggCCCCC | GGTCAATTTCCCTGGTAGGG**TACAAT**TCTTA | -119 | 7.08 |
| 36 | sll1924 | - | TGTGAcaataaACACC | AAAACAGTTTACTTGTTTC**TATAAT**TTTCCC | -90 | 7.07 |
| 37 | slr0509 slr0510 | - - | GGTGAgttaccCCTCG | CCCCAGGGGAAATGGCAATGGGTT**TATGCT**C | -133 | 7.07 |
| 38 | sll1376 | - | TGTGGatgctcTCACG | GGTAATCAGGTCAATGTCTCGATC**AAAAGT**G | -405 | 7.03 |
| 39 | slr0292 | - | CGTGAtccccgTCCCC | AGATCGCCCTCGATCGCC**TGACTT**ACCTAG- | -31 | 7.03 |
| 40 | sll0321 sll0322 | - hypF | GGTGAgcttgaGCTCT | GTTTGCCGTGCTTATGCATGGCCA**TGGTTT**A | -89 | 7.02 |
| 41 | ssl2245 sll1130 | - - | GGTGActattcCCACG | AATTGTTTACTTTTCTGC**TAATAT**GTCTTCT | -154 | 7.02 |
| 42 | sll0083 sll0084 sll0085 sll0086 | gmhA hisB - - | AGGGAaaatcgTCCCC | AGTGACCAAACGCTGACCCCTT**TAACAT**TAG | -164 | 7.01 |
| 43 | **sll0443 sll0444 sll0445 sll0446 sll0447 sll0448 sll0449** | - - - - - - - | GGTGAttaagtTCCCA | TTTTATCTGGTTATCCTGGAG**GCAATT**CAAA | -371 | 7.01 |
| 44 | slr0545 | - | TGTGAttggttCCGCT | GTTTTCGAACGATCAATTAAACAAT**TAAAAT** | -155 | 7.00 |
| 45 | sll0564 sll0565 | - - | GGTGCttgatcACACA | TTTCCGGGCGGAGGGCATGTGGG**TGTCCT**GG | -503 | 6.98 |
| 46 | slr1395 | - | GGTGAgaatttCCACC | CAGCCGGCGGTGGCCACTTCCAC**CAAAAT**GG | -332 | 6.98 |
| 47 | slr1906 slr1907 | - - | TGTGAgtttctACTCA | GTCAGTGCTCAACTGGATTAGG**GATTTT**GGC | -600 | 6.97 |
| 48 | slr1838 slr1839 slr1840 | ccmK ccmK - | CGTGGtcaacaGCACT | AGGACCTTGCAGCTTGATTTGCCCG**TGCCAT** | -125 | 6.97 |
| 49 | sll1570 sll1571 sll1572 | - - dnaE | AGGGCgaaaaaTCACT | TAAATTATCAAATTCCCGGTGAA**TTTCTT**GC | -680 | 6.96 |
| 50 | slr1484 slr1485 slr1488 slr1489 | - - - pchR | GGTGTttaatgTCGCA | ACTAAACGTCTATAATGCTATAGC**TATTAA**T | -109 | 6.96 |
| 51 | ssr2848 | - | TGTGAtctaaaTCACC | TGGTTAATGTGATAGTTTC**TCTGGT**GGAAAG | -88 | 6.96 |
| 52 | slr0316 | - | GGCGCttattcTCACG | GTAGGCTGTAACGCTGTCGGAC**AAACTT**CTG | -232 | 6.94 |
| 53 | slr0417 slr0418 | gyrA - | TGTGGaggctcCCACC | CTGGCCAATGGTAGAATAGAGGC**TGTTTT**GA | -106 | 6.93 |
| 54 | slr2058 slr2059 slr2060 | topA - - | TGTGAcaaaagTCCCC | TTTTTAAGCCGGAGCTTTAGTGAG**GAGATT**T | -255 | 6.92 |
| 55 | sll0271 sll0272 sll0273 sll0274 | nusB - - - | GGTGTtgggcaTCACG | AGGGTATTGCCCTGGGCTTGC**AACGGT**CCGA | -473 | 6.90 |
| 56 | slr1950 slr1951 slr0989 slr0990 slr0992 | - - - - - | TGTGCatttttCCACA | ACCATCGTCACTACGGCGAATTG**CAGGCT**GG | -227 | 6.88 |
| 57 | slr1349 slr1350 | pgi desA | GGTGGttaaccACACA | CTGTCGCCAAACTTGCCCCAT**TTTGCT**CCGA | -199 | 6.88 |
| 58 | sll1880 | - | TGTGGctacttACTCA | ATTTACTTAGAATAATCC**TAATTT**ACCTAAA | -69 | 6.87 |
| 59 | slr2127 | - | AGTGGtgctgaACACC | TAGAGCTATTAAATATTCGGGA**TCAAAT**TGG | -380 | 6.83 |

**Table S13**. Predicted CRP binding sites in *Trichodesmium erythraeum* IMS101 genome at *P* < 0.01

| Rank | Transcription Unit | Names | CRP TFBS | Downstream region of the CRP TFBS | CRP site position | Score |
| --- | --- | --- | --- | --- | --- | --- |
| 1 | Tery_3081 Tery_3082 Tery_3084 | - - - | GGTGGcctcatTCACA | CTCTAGTAATTTACGCCATGTCC**TACGTT**TA | -312 | 8.71 |
| 2 | Tery_0754 Tery_0755 | - - | TGCGCcttaaaCCACT | CGGCCACCTCTCCAGAAAACAA**TATTAT**ATA | -488 | 8.62 |
| 3 | Tery_1627 | - | CGTGAgactatTCACT | CTTTTCTTCTACTTCTATTTC**TAATCT**GATT | -349 | 8.59 |
| 4 | Tery_2346 Tery_2345 | - - | TGTGAtgactaTCCCC | TTAATTTCAATGGATTGAG**TATAAT**AAGGTG | -85 | 8.57 |
| 5 | Tery_2879 Tery_2878 | - - | GGTGTaaaaaaTCTCA | AAGTACCGTTAATAAAATACTT**TGTGTT**TAA | -128 | 8.53 |
| 6 | Tery_4209 | - | CGTGAgcatttCCACA | CTTTTTTGACGCACTACCAGT**TATGGT**TTGG | -634 | 8.43 |
| 7 | Tery_3519 | - | GGTGAagcttcTCTCC | AACCTTCGATATTTTTTTGTTG**TATTTT**TAC | -476 | 8.39 |
| 8 | Tery_4643 | - | TGTGCtaacatACACT | TACTAAATATGCACATTAACA**TATAGT**TCGG | -361 | 8.38 |
| 9 | Tery_4029 Tery_4028 | - - | GGTGAacattaCCACC | GAATGCTATTTTTGTTTCAA**TAGTTT**TTGAA | -440 | 8.37 |
| 10 | Tery_2502 | - | AGTGCgatcgcTCACT | TTTTTGTTTTGGATTATATCAGA**TAATTT**GA | -302 | 8.34 |
| 11 | Tery_3916 | - | TGTGTttgataTCACA | GTCTAATGCTCCTTGTTTCACAGCA**TTTATT** | -102 | 8.31 |
| 12 | Tery_2602 Tery_2601 Tery_2599 | - - - | TGAGAagacccTCACT | ACCCTAGCGACTGCTCTATTTT**TAACTT**TTT | -158 | 8.30 |
| 13 | Tery_0412 | - | GGTGTtaggttTCACT | ATAATTATACCTAAGTTAAA**TTTTTT**AGGTG | -66 | 8.30 |
| 14 | Tery_2476 Tery_2475 | - - | GGTGTataattTCACA | TATTTGGGTAAGTTCTGA**TTTATT**TATGCCT | -212 | 8.27 |
| 15 | Tery_0785 | - | TGTGTtattaaTCTCA | AGTTTTTATCATTTTGGGCT**TAGGCT**TATGT | -337 | 8.27 |
| 16 | Tery_4894 | - | TGTGAtttactACACT | GATATATCAACTTGATCAAT**TATTCT**GGCAA | -159 | 8.27 |
| 17 | Tery_1754 | - | GGTGTcggttaTCGCT | GACTTGAATTGGCGACAATAGT**TATGAT**ATG | -771 | 8.26 |
| 18 | Tery_3484 Tery_3485 | - - | AGTGCttttttCCACA | GAACGAAAAGTAATAATTAGGG**TAACTT**GAC | -69 | 8.21 |
| 19 | Tery_0457 | - | GGTGAacttttGCTCA | GATATTATTTATAATTGTTATA**TATGAT**AGA | -83 | 8.20 |
| 20 | Tery_3155 | - | TGTGTtatcttTCACC | TTGGCATCGGGTAGAAAAC**TTACCT**AAGAAC | -701 | 8.19 |
| 21 | Tery_4301 | - | TGTGGgtctgcTCACG | TTATTGATATACTACTAGAGTGG**TGACTT**CG | -166 | 8.17 |
| 22 | Tery_0957 | - | TGTGTttcaatTCCCA | CTTAGCATTAATGGTAAATTATACA**TATACT** | -293 | 8.17 |
| 23 | Tery_1155 Tery_1154 | - - | TGTGAacaaatTCTCG | ATCATTTTTCTAAATCTAG**TATTTT**AGGATA | -526 | 8.17 |
| 24 | Tery_3036 | - | TGTGGtgctaaACACG | AACGTGATCAAAATGCAGCAGTAGA**TATATT** | -423 | 8.16 |
| 25 | Tery_4047 | - | TGTGCtgcaaaACACG | ACCGTGATGAAAATGAAGAAATAAA**TATATT** | -514 | 8.14 |
| 26 | Tery_0283 | - | GGTGGggcgagGCACC | CCAATGATTTTGATCAAGTTTTACA**TAAGTT** | -96 | 8.14 |
| 27 | Tery_2090 | - | AGTGCtccaaaTCCCA | CTATTAAGCTATCGTACATC**TAAATT**TTATA | -240 | 8.13 |
| 28 | Tery_3175 Tery_3177 | - - | TGTGGttttgtTCGCA | ATTAATTAGGGAAAAGGTTCACGAC**TAAAAT** | -256 | 8.13 |
| 29 | Tery_2530 | - | AGTGAtttgaaTCACA | TTAAAATCTTATAAGGGGTAACC**TACTCT**AT | -133 | 8.12 |
| 30 | Tery_4669 Tery_4668 | - - | AGTGTgatgttGCACT | CTAGCAAAAAGCAGTTACAATCTG**TAAACT**T | -120 | 8.12 |
| 31 | Tery_1886 | - | GGTGAtacccaTCACT | TACCGTAATAAGATATTTAGAGA**TATCAT**AG | -144 | 8.12 |
| 32 | Tery_1324 | - | CGTGAgttcacACACT | GTTATTTTGAAGTGATACCAAT**TTTTAT**AAG | -589 | 8.11 |
| 33 | Tery_3906 Tery_3907 | - - | TGTGAaaaataCCACA | ATTAAAGCAAGTCTATTCTCTGAGA**TCTAAT** | -570 | 8.10 |
| 34 | Tery_0595 | - | AGTGGcaattcTCTCC | AATTCAATGTCCCCAAATTCC**TAGATT**TAGA | -728 | 8.09 |
| 35 | Tery_0925 | - | CGTGAtcccaaTCACG | AAAGGGATTCATTTGGTAG**TAGATT**TTACAG | -376 | 8.09 |
| 36 | Tery_2748 | - | AGTGTtttcggCCACT | CATAAATTTGTTGGAATAATC**TAATTT**ATAG | -291 | 8.08 |
| 37 | Tery_1557 Tery_1556 | - - | AGTGAtgatttCCCCA | GTTAACCGTGGCACTATAGTCTA**TAGACT**TT | -146 | 8.08 |
| 38 | Tery_1893 Tery_1892 Tery_1891 Tery_1890 | - - - - | CGTGTttttacTCACT | TTCTAAATGTGTAGTGCTATA**TCTGTT**CTTG | -349 | 8.07 |
| 39 | Tery_1813 | - | AGTGAcacacaTCACT | AATAAACATAAACATAATCACAT**TAAACT**TA | -105 | 8.04 |
| 40 | Tery_0339 | - | AGTGTcaccatTCTCA | GAAAGCCAAGAATTAAAAATT**TAATCT**CAAA | -186 | 8.04 |
| 41 | Tery_3156 | - | AGTGAgattatACACC | AATAAATATAGAAGAGTCCA**TCGAAT**CAGTA | -159 | 8.04 |
| 42 | Tery_1085 | - | GGTGGcctcatTCACA | CTCTAGTAATTTACGCCATGTCC**TACGTT**TA | -310 | 8.03 |
| 43 | Tery_4986 Tery_4987 | - - | AGGGAatggggTCTCA | GGTCTAGGTCTTACTAGAATT**TAAGCT**AAGA | -261 | 8.02 |
| 44 | Tery_2055 | - | TGTGCataattTCACC | AATACTAACTTTAGGTTA**TATTAT**ATATAGT | -428 | 8.02 |
| 45 | Tery_4732 | - | AGTGAtgcctaTCCCA | CAGTCACCTTCTGTCAATTTAT**TCATGT**GAC | -757 | 8.02 |
| 46 | Tery_4942 | - | GGTGAtaaagcCCTCA | CAATTAGACAAATGTACT**TATGAT**AATAAAT | -440 | 8.01 |
| 47 | Tery_1727 | - | TGTGAgtttccCCCCC | AGCACTCTTCTGTGCCAGAAC**TGGCAT**ATGG | -128 | 7.99 |
| 48 | Tery_3577 | - | AGTGTtaggttTCACT | CTTAAACCCAACCTACGCAATT**TCCTTT**TTG | -241 | 7.98 |
| 49 | Tery_2254 | - | AGTGCtggcgaTCGCT | GATTATTAGAAAATAGTTTTAC**TATCTT**AAA | -266 | 7.98 |
| 50 | Tery_1044 | - | TGTGTcattgcTCACA | AGTTATCTCATACTAAGTAA**TAACAT**TTTGA | -130 | 7.98 |
| 51 | Tery_2466 | - | AGTGTatatcaTCACA | ACTGGCCACTTAGAAATA**TATCAT**TTGGTGG | -174 | 7.98 |
| 52 | Tery_3423 | - | CGTGGgagtatTCACG | AAATTCATCTATAATCAATCGAG**CAGAAT**TA | -96 | 7.98 |
| 53 | Tery_2433 Tery_2432 | - - | GGTGActaggtCCACT | GTACTAAGAATTGTCCCC**TGATTT**TTCGGTC | -780 | 7.97 |
| 54 | Tery_3369 Tery_3368 Tery_3367 Tery_3366 Tery_3365 | - - - - - | CGTGAatcccaTCTCA | ACCTCAGATAACAGAGAG**GAGAAT**TAGAGAG | -32 | 7.97 |
| 55 | Tery_3422 | - | CGTGAatactcCCACG | GTAACTTTACAATATGACGTGGGC**TTTTTT**G | -663 | 7.96 |
| 56 | Tery_2549 | - | TGTGTgcaaggTCCCT | ACAGAGGAATGGGGAATTGA**TAGAGG**AGGTA | -449 | 7.95 |
| 57 | Tery_4012 Tery_4013 | - - | AGTGAggccttCCTCA | AAATACCAAAGAATTTGACACAA**TAGAAT**TA | -61 | 7.94 |
| 58 | Tery_1570 Tery_1569 | - - | AGTGAttacttACCCC | TTTGTTAAAAAATTACTCTTAT**AACACT**AAG | -467 | 7.94 |
| 59 | Tery_0929 | - | AGTGAaataaaGCACA | TTAAATAAACAATAACCCTCAG**GACGTT**GAA | -534 | 7.94 |
| 60 | Tery_3065 | - | GGTGAgtatctCCACA | ATGCATCGCGCCGTAGAAAAAT**TAGGCT**TAC | -32 | 7.93 |
| 61 | Tery_3028 | - | GGTGAgtatctCCACA | ATGCATCGCGCCGTAGAAAAAT**TAGGCT**TAC | -671 | 7.93 |
| 61 | Tery_3101 | - | GGTGAgtatctCCACA | ATGCATCGCGCCGTAGAAAAAT**TAGGCT**TAC | -408 | 7.93 |
| 61 | Tery_4857 | - | GGTGAgtatctCCACA | ATGCATCGCGCCGTAGAAAAAT**TAGGCT**TAC | -34 | 7.93 |
| 61 | Tery_3858 | - | GGCGAcaaatgTCACC | AAATGTACCCATTACCAAA**TAATTT**GGTTAT | -468 | 7.93 |
| 62 | Tery_4095 Tery_4094 | - - | GGTGAggaataACACA | AAAATTAATTAAATGCTAAACTCAA**AAATTT** | -377 | 7.92 |
| 63 | Tery_1286 Tery_1287 Tery_1288 | - - - | TGTGAgctaacGCTCC | ACTAAAAATAGTTAGTATAAGCGT**TTGAGT**A | -693 | 7.92 |
| 64 | Tery_0143 Tery_0144 | - - | TGTGGaataatACCCA | AAAATACAATCAAACAAATTTCG**TATATT**AT | -102 | 7.91 |
| 65 | Tery_2051 | - | TGTGTtaggttTCACT | TCCTTGTTGAGGAGCCTACTT**TAATAT**TGCC | -170 | 7.91 |
| 66 | Tery_1943 | - | AGTGCttttgtTCCCT | TATGAGGTATAGTAACAGTAATGAG**TAAATT** | -383 | 7.91 |
| 67 | Tery_4648 | - | TGTGGgatataACCCA | GTTATTACTCATAAGGCT**TATAAT**ATTTATT | -225 | 7.91 |
| 68 | Tery_2056 | - | GGTGAaattatGCACA | CCAAGGTTATTTCATGGAACTTTG**AAAATT**G | -191 | 7.90 |
| 69 | Tery_3936 | - | GGTGAaacctaACACA | GAATTTATTTTGGTTGGGT**TACTCT**TGAGGT | -274 | 7.90 |
| 70 | Tery_4080 Tery_4081 Tery_4082 | - - - | TGAGAactaaaACACT | CTCAAAAAGCGAAAGTAGG**TATAAT**GAGGTT | -389 | 7.90 |
| 71 | Tery_1822 | - | TGTGCgtaataTCACT | TAAAAAATAAAACGCTTGATAACTA**TAGCAT** | -285 | 7.89 |
| 72 | Tery_3213 | - | TGGGGtgtatcTCACC | AAGGTGGAATTCGCTGTATGT**TTAAAT**AATC | -245 | 7.89 |
| 73 | Tery_3151 Tery_3150 Tery_3149 Tery_3148 Tery_3147 Tery_3146 Tery_3145 | - - - - - - - | AGTGGttaataGCTCA | GTATCGCAAAGGTTACTAGGC**TATTGT**TTAG | -156 | 7.88 |
| 74 | Tery_2827 | - | TGTGCttgcttACCCT | CCCAATATCCTAACCTTTTTCA**TCAACT**ATT | -405 | 7.88 |
| 75 | Tery_3303 | - | AGTGTatttatACACA | ATACAATTAGGAATAGATAAAT**TTTAAT**AAC | -39 | 7.88 |
| 76 | Tery_4264 Tery_4265 | - - | GGTGAatatttTCACT | TGAGTCTATAAGTTGGAAGCGAGT**GGTGAT**C | -628 | 7.87 |
| 77 | Tery_4937 | - | AGTGTacctcaTCACG | TGGGGAAAGTGCTGTATA**TTAGTT**GTTAAGT | -491 | 7.87 |
| 78 | Tery_3093 Tery_3094 | - - | GGTGAgtatctCCACC | ATGCATCGCGCCGTAGAAAAAT**TAGGCT**TAC | -34 | 7.87 |
| 79 | Tery_3619 | - | TGTGAtccttaACACA | TTATAATTTTTTTTGACAAAAGAG**AATACT**A | -182 | 7.87 |
| 80 | Tery_4208 | - | TGTGGaaatgcTCACG | AGGTGACAAGCTATCTGAA**AACTAT**ATATAG | -598 | 7.85 |
| 81 | Tery_2227 | - | TGTGTttctttACCCT | TAGTTTCCACCTCTAGGCGTATCGA**TTTTTT** | -643 | 7.85 |
| 82 | Tery_4817 Tery_4818 | - - | GGTGGcagtaaTCTCA | TTCTTTAGCTATAGCTTGGTA**TCAATT**CAAG | -277 | 7.85 |
| 83 | Tery_3381 | - | GGTGAtgattaTCTCA | GCGCCAAAAGTAACGATGCTCATT**TAATAT**T | -218 | 7.84 |
| 84 | Tery_2566 | - | GGGGTatttttTCACA | GAATAAGATACTCTCTACTC**TTTTTT**CCATT | -392 | 7.83 |
| 85 | Tery_5008 | - | GGTGAtggcgaTCGCA | AACCTGATTTCAGGCAGT**TGCTAT**TTGTGCC | -505 | 7.82 |
| 86 | Tery_3659 | - | AGTGAtaaataTCTCA | AATTAAATTAATCTAGAATCA**TAATTT**AGGA | -41 | 7.82 |
| 87 | Tery_4839 | - | TGTGGatgagtCCCCA | TGGTTGGTACAAGGAGTAA**AAGAAT**GGA--- | -29 | 7.82 |
| 88 | Tery_3985 | - | TGTGAcaaaaaTCCCG | TTAATGATCAAGAATTATAAA**TTTAGT**TCCT | -137 | 7.81 |
| 89 | Tery_4087 | - | GGAGAtgaaatGCACC | TATCACATCTTTGAACAAA**TAATCT**CTATTA | -684 | 7.80 |
| 90 | Tery_3038 | - | AGTGCttatatTCTCG | GATATTGGACAGATAGTACCA**TTGACT**AGGG | -515 | 7.80 |
| 91 | Tery_3991 | - | GGAGTcaggagTCACA | ATTTTCAAGTTTTTTAGT**TAAAAT**TAGTTAT | -224 | 7.79 |
| 92 | Tery_4328 Tery_4329 | - - | TGAGAaggctaTCACC | TAGGCTTGAAGAAACCCTCTGGAGG**TTTTTT** | -315 | 7.79 |
| 93 | Tery_0199 | - | GGTGCagatgtACCCA | TTCAACCTAATAATCTCTGTGAT**TAGCCT**GT | -770 | 7.78 |
| 94 | Tery_4050 | - | AGTGGtaaaccCCGCC | CTGCTTGTCCAGTCAGGCC**TAATAT**CTAAGT | -61 | 7.78 |
| 95 | Tery_3152 | - | GGTGAcaagctTCACA | GTTTTTGCACATAATCTTAGGAG**GATGTT**CC | -532 | 7.78 |
| 96 | Tery_0728 Tery_0727 | - - | TGTGAttcaaaTCTCC | AGACCTTGATAGACTGTA**TGGGAT**GAAAAAC | -298 | 7.77 |
| 97 | Tery_4675 | - | AGTGCttgatcACACA | TCGAACAGAATATGTAAT**TAATTT**TGCTTAT | -339 | 7.76 |
| 98 | Tery_3712 Tery_3713 Tery_3714 | - - - | GGTGAgagcgaTCGCG | ATGATCTAAAAACATCCT**TACTCT**AAAAGTA | -254 | 7.76 |
| 99 | Tery_0081 Tery_0080 | - - | AGTGAaactttGCTCA | GGTTGAATCAACTATTTGTCTAAA**CATATT**A | -255 | 7.76 |
| 100 | Tery_3954 Tery_3955 | - - | TGTGCtgctagCCACC | TTTTGACAAGGGCTGTATCTAAA**AATAAT**GG | -731 | 7.76 |
| 101 | Tery_0486 | - | AATGAcgaataTCACT | TTCTCGTATAGTATTGAATCACCTA**TAAGGT** | -84 | 7.75 |
| 102 | Tery_2915 | - | GGTGGcttttcTCTCA | AATTATGTAATTATATTTATGA**TGTATT**TGT | -602 | 7.75 |
| 103 | Tery_3932 | - | TGAGGttttcaCCACT | CTTTACTTTGGTTACTAAAGC**TAAATT**TAAA | -565 | 7.75 |
| 104 | Tery_0122 Tery_0121 Tery_0120 | - - - | GGTGCtagttaTCCCA | TTAGTATAAGTTATTATCC**AATAAT**AAGACT | -701 | 7.74 |
| 105 | Tery_0882 | - | TGTGTttttgcCCTCC | TGTGTTGGTTCTGATAGGGAC**TATTGT**TGGG | -251 | 7.74 |
| 106 | Tery_3216 | - | GGTGAaaagccCCTCA | AAACTATTGTTGGATGAGGGTT**GAGACT**GAA | -626 | 7.73 |
| 107 | Tery_4912 Tery_4913 | - - | CGTGAgctctcTCACA | CGTGATCGACTCAAACAAAAAGA**AAATTT**GT | -467 | 7.73 |
| 108 | Tery_2268 Tery_2267 | - - | GGTGAcggtcaACACT | ATATGTGCACAAAAAAATGCGTAAG**TCCTGT** | -267 | 7.73 |
| 109 | Tery_4011 Tery_4010 Tery_4007 | - - - | TGAGGaaggccTCACT | ATCTCAATATATTGACTA**TAAATT**GGCCTTT | -263 | 7.73 |
| 110 | Tery_1113 | - | TGTGTtggaaaGCGCT | CATACAGGTTCAGTTGTACCTC**TACTTT**TTG | -363 | 7.73 |
| 111 | Tery_3227 | - | TGTGGatgtcaACGCT | CAAGACTCAGAGGGTTCAACTGCT**TGAATT**G | -60 | 7.73 |
| 112 | Tery_0435 Tery_0434 | - - | CGTGCcttgtgTCTCA | TTACAAAAGTTAACTTTTTGACAA**AATACT**A | -539 | 7.72 |
| 113 | Tery_1481 Tery_1480 Tery_1479 | - - - | CGAGAaaacaaGCCCA | ACCGTGAATCATCTGCCATAA**TACTGT**AAAG | -496 | 7.72 |
| 114 | Tery_2982 | - | GGTGTaaaaaaACTCA | GTCTATCTCCAAATTAGCTTGAAAA**TAACAT** | -139 | 7.71 |
| 115 | Tery_3304 | - | TGTGTataaatACACT | AGGTCGTATGTAGTAATCT**TATTTT**ATTCTC | -516 | 7.71 |
| 116 | Tery_0198 | - | TGGGTacatctGCACC | TAACTCACTCTAAATCAAGATT**TAATAT**TTG | -117 | 7.71 |
| 117 | Tery_1191 Tery_1190 Tery_1189 | - - - | AGTGGccatgcCCTCT | TGTTTCCATCGATGGTGCGA**TCGCTT**TAACA | -550 | 7.71 |
| 118 | Tery_4419 Tery_4420 | - - | AGTGAtcgctaTCACT | TAACATTAACTGAAAACAGTT**TTTCAT**TATT | -405 | 7.70 |
| 119 | Tery_0273 | - | TGTGTtgggttTCGCC | GGCTTAAGCTTACCTACGGGAGGGG**GAGCTT** | -730 | 7.70 |
| 120 | Tery_4492 | - | TGTGCttaactGCTCC | CTAATATGATAGTGTATA**TACTGT**TTCGTCT | -316 | 7.70 |
| 121 | Tery_2327 Tery_2328 Tery_2329 Tery_2330 Tery_2331 Tery_2332 Tery_2334 Tery_2335 | - - - - - - - - | TGTGCttctttACTCT | ATCTTCGACTATACATCTAAGTT**TAGAAT**TG | -414 | 7.70 |
| 122 | Tery_2779 | - | TGAGAtctactTCACG | ATAGTGTATTCTTTTTTTTAGAGG**TAGTAT**A | -761 | 7.70 |
| 123 | Tery_4825 | - | AGAGGcagctcTCACT | AAACTAAGCTACCGTATTATAGGC**TAAATT**C | -742 | 7.70 |
| 124 | Tery_4381 | - | GGTGTttgtatCCCCG | TACCCGTTAGCCCAAACCTA**TAAACT**AACAT | -729 | 7.70 |
| 125 | Tery_1039 | - | TGTGTatagcgGCACT | GCGTAAGCCCTGAATGTTTTTTTTC**TATTGT** | -199 | 7.69 |
| 126 | Tery_4418 | - | AGAGAcatttaTCACA | AAATGTAAAATTAATGCATCA**AAAAAT**CCCT | -334 | 7.69 |
| 127 | Tery_1779 Tery_1778 Tery_1777 | - - - | TGTGGacctatTCTCA | TATACTACTTATAATCAATCGCT**TCAAAT**AA | -558 | 7.69 |
| 128 | Tery_4786 Tery_4787 | - - | TGTGCagctagACCCT | TGCCATTGTGCTCAAGCTGACGGC**TAAAGT**A | -131 | 7.69 |
| 129 | Tery_3232 Tery_3231 Tery_3230 | - - - | GGAGCgatcgcTCACC | TTGAAGATAGTTAATTTG**TACAGT**TTTATGG | -724 | 7.69 |

**Table S14**. Predicted CRP binding sites in *Thermosynechococcus_elongatu* BP1 genome at *P* < 0.01

| Rank | Transcription Unit | Names | CRP TFBS | Downstream region of the CRP TFBS | CRP site position | Score |
| --- | --- | --- | --- | --- | --- | --- |
| 1 | tll2194 tll2193 tll2192 | - - glyS | TGTGAcgatgaTCACA | ACTTAGCCGCCTTTGATT**GCGTTT**CGCCAAA | -59 | 7.73 |
| 2 | tll0332 tll0331 tll0330 tll0329 tll0328 tll0327 | - pdxH ureB - - - | TGTGActcagaTCACC | GAAAATAAAAATTATTCTAAAGGA**TAAAAT**T | -200 | 7.70 |
| 3 | tlr0333 | - | GGTGAtctgagTCACA | AAAACATTCGAAAATTAAAAACAG**TTTATT**A | -303 | 7.53 |
| 4 | tlr1736 | - | GGTGAgattggGCACA | CTGATTATCTTAGGCGCTGTC**TAAGTT**GGTG | -56 | 7.49 |
| 5 | tll0251 | - | GGTGTgccagtTCACA | CACCTATCCCCGATCAAG**TATGCT**GAGCACG | -64 | 7.47 |
| 6 | tll1264 | - | CGTGGcacattTCACA | TGGACCGGGTTGCTACTGTC**GAGAGT**GAGGA | -676 | 7.32 |
| 7 | tlr0738 tlr0739 tlr0740 tlr0741 tlr0742 tlr0743 | sigF - ycf45 hemE - - | TGTGGtcaagaTCACA | GGGAAATGAGGGATATCTGAG**CATACT**AGGT | -160 | 7.21 |
| 8 | tsr2195 tlr2196 | - menA | TGTGAtcatcgTCACA | GTCATTCTATCGGGGGATAGGT**CATACT**AGA | -338 | 7.21 |
| 9 | tll0142 tll0141 | - - | CGTGCcagcatTCACT | ATTTTGGCAGTCAATTTT**CACAAT**GGCGGAA | -112 | 7.12 |
| 10 | tlr0334 tlr0335 | thiC - | GGTGTctgcggTCACA | GCAGGCTGAGAGCAAACCCTTA**GAACCT**GAA | -80 | 7.04 |
| 11 | tlr0651 tlr0652 tlr0653 tlr0654 tlr0655 tlr0656 | - - - - - - | AGTGGgtagccTCACA | GGGCGATCGCTCCCAATTCGA**TCACCT**CTCC | -43 | 7.03 |
| 12 | tlr2337 tlr2338 | - - | AGTGGcgaatcTCTCG | CTGAGGGGCAAGTCCACGCCTTA**TCCAGT**TG | -213 | 7.02 |
| 13 | tlr1546 tlr1547 tlr1548 tlr1549 | - purH - - | AGTGTtgccaaTCACC | TGCCAAAAGTCTGGACTTAGGAGC**AACTGT**T | -147 | 7.00 |
| 14 | tsl1138 | - | CGTGAtgaagtCCACT | AAGGAGCTTGACAACGGCA**CAATTT**GGCGTA | -100 | 6.98 |
| 15 | tlr1168 tlr1169 tlr1170 tlr1171 | ycf20 - - - | GGTGCtgtccaTCACG | CCAGCAAAAAGATGACTGGCCTGTT**TCACTT** | -490 | 6.92 |
| 16 | tll1043 | - | GGTGAagatacGCCCA | AATGCCTCCATCAAAAAGACCATCA**TACTTT** | -407 | 6.91 |
| 17 | tlr0967 tsr0968 tlr0969 | - - - | AGTGCcctacaTCACC | CCTGATCCAAGAGATAGAGCACAG**TCTTCT**G | -106 | 6.88 |
| 18 | tll2024 tll2023 | - - | TGTGTattcaaTCTCA | CCAACTGCTGAGGTCTATTC**TACAGT**TAGAG | -111 | 6.88 |
| 19 | tll2476 tll2475 tll2474 | - trpB - | CGTGTcctgtaCCACT | GTATTTCCCGTATTCGCTAGAT**TACAGT**CAG | -57 | 6.87 |
| 20 | tlr1890 | - | AGTGCtttaagCCACT | CCTCTAAGCCATTTATTAAAGCAAG**TAAAGT** | -198 | 6.84 |
| 21 | tll1403 | - | GGTGAgtcgctGCTCA | AAAAGCATTGGAGACCTTGACCCC**TATAAT**G | -35 | 6.80 |
| 22 | tlr2203 | - | GGTGAtctattTAACA | AATCTTGAATGAGGGTGAGACTTC**TATTGT**G | -221 | 6.79 |
| 23 | tll0507 tll0506 | - gltX | TGTGCgcgatcGCACA | AATTATAATTTTAGCGCAGATT**TCCCCT**GCT | -202 | 6.78 |
| 24 | tll0439 | trpA | TGAGAtgccccTCACT | GCCACCTGCCACTATAGGTATCTCA**TATTCT** | -89 | 6.74 |
| 25 | tsr1131 | - | TGTGCgtcaatCCACA | AACTGCCTACTTGTCCCTT**GACAGT**CCTCCC | -104 | 6.73 |
| 26 | tll2233 tsl2232 tll2231 tll2230 tll2229 tll2228 tll2227 tsl2226 | - - - - - - - - | CGTGGgtctttTGACT | GCGCGCTGGTAACTTTTTCGT**TATATT**GCTA | -367 | 6.72 |
| 27 | tll0032 | - | CGTGAacgattTCACG | AAATTCTTGTTCAGTTTC**ATTAAT**GAGACCT | -74 | 6.70 |
| 28 | tll0113 | ftrC | TGTGGggaaacTCACA | AGTCCGGTTCTTAGGGGGCGGGGAT**GGCAGT** | -185 | 6.70 |
| 29 | tsr0033 | nblA | CGTGAaatcgtTCACG | CTGAACATTTTATCCTTTGCGCAGG**TGATAT** | -114 | 6.68 |
