# Supplementary figures and images for: Computational prediction of cAMP receptor protein (CRP) binding sites in cyanobacterial genomes
Source: BMC Genomics. 2009 Jan 15;10:23. doi: 10.1186/1471-2164-10-23 (PMC2633013; doi:10.1186/1471-2164-10-23)

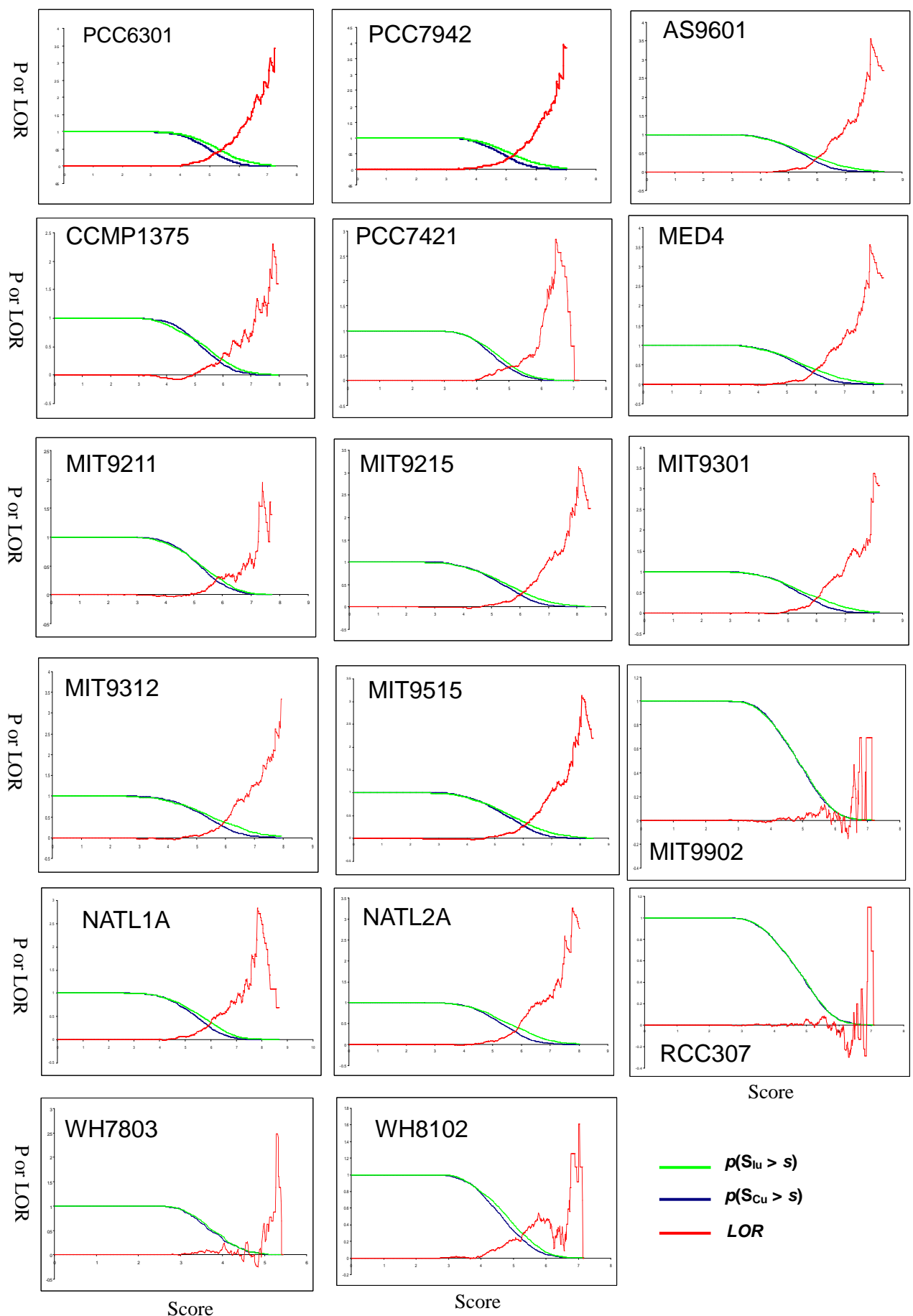

Supplement: Additional File 3 — Figure S2. Genome-wide scanning for CRP-like binding sites in the 17 genomes that do not encode a CRP protein. [file 1471-2164-10-23-S3.pdf]
